# Safety and Allele-Specific Immunogenicity of a Malaria Vaccine in Malian Adults: Results of a Phase I Randomized Trial

**DOI:** 10.1371/journal.pctr.0010034

**Published:** 2006-11-24

**Authors:** Mahamadou A Thera, Ogobara K Doumbo, Drissa Coulibaly, Dapa A Diallo, Issaka Sagara, Alassane Dicko, David J Diemert, D. Gray Heppner, V. Ann Stewart, Evelina Angov, Lorraine Soisson, Amanda Leach, Kathryn Tucker, Kirsten E Lyke, Christopher V Plowe, for the Mali FMP1 Working Group

**Affiliations:** 1 Malaria Research and Training Center, University of Bamako, Bamako, Mali; 2 Malaria Vaccine Development Branch, National Institute of Allergy and Infectious Diseases, National Institutes of Health, Bethesda, Maryland, United States of America; 3 Department of Immunology, Walter Reed Army Institute of Research, Silver Spring, Maryland, United States of America; 4 United States Agency for International Development, Washington, D. C., United States of America; 5 GlaxoSmithKline Biologicals, Rixensart, Belgium; 6 Statistics Collaborative, Washington, D. C., United States of America; 7 Center for Vaccine Development, University of Maryland School of Medicine, Baltimore, Maryland, United States of America

## Abstract

**Objectives:**

The objectives were to evaluate the safety, reactogenicity, and allele-specific immunogenicity of the blood-stage malaria vaccine FMP1/AS02A in adults exposed to seasonal malaria and the impact of natural infection on vaccine-induced antibody levels.

**Design:**

We conducted a randomized, double-blind, controlled phase I clinical trial.

**Setting:**

Bandiagara, Mali, West Africa, is a rural town with intense seasonal transmission of Plasmodium falciparum malaria.

**Participants:**

Forty healthy, malaria-experienced Malian adults aged 18–55 y were enrolled.

**Interventions:**

The FMP1/AS02A malaria vaccine is a 42-kDa recombinant protein based on the carboxy-terminal end of merozoite surface protein-1 (MSP-1_42_) from the 3D7 clone of *P. falciparum,* adjuvanted with AS02A. The control vaccine was a killed rabies virus vaccine (Imovax). Participants were randomized to receive either FMP1/AS02A or rabies vaccine at 0, 1, and 2 mo and were followed for 1 y.

**Outcome Measures:**

Solicited and unsolicited adverse events and allele-specific antibody responses to recombinant MSP-1_42_ and its subunits derived from P. falciparum strains homologous and heterologous to the 3D7 vaccine strain were measured.

**Results:**

Transient local pain and swelling were more common in the malaria vaccine group than in the control group (11/20 versus 3/20 and 10/20 versus 6/20, respectively). MSP-1_42_ antibody levels rose during the malaria transmission season in the control group, but were significantly higher in malaria vaccine recipients after the second immunization and remained higher after the third immunization relative both to baseline and to the control group. Immunization with the malaria vaccine was followed by significant increases in antibodies recognizing three diverse MSP-1_42_ alleles and their subunits.

**Conclusions:**

FMP1/AS02A was well tolerated and highly immunogenic in adults exposed to intense seasonal malaria transmission and elicited immune responses to genetically diverse parasite clones. Anti-MSP-1_42_ antibody levels followed a seasonal pattern that was significantly augmented and prolonged by the malaria vaccine.

## Editorial Commentary

**Background:** In sub-Saharan Africa the burden of death and disease from malaria is particularly severe. Most affected are young children under the age of five, in whom natural immunity against the malaria parasite has not yet developed. There are not yet any approved vaccines that would reduce this burden, although many research groups are currently developing potential vaccines. One such candidate vaccine is FMP1/AS02A. This vaccine is designed to trigger an immune response against a protein (merozoite surface protein-1, or MSP-1) found on the surface of the infectious, blood-stage form of the malaria parasite. Early-stage clinical trials have already been performed in healthy people in the United States, who were not exposed to clinical malaria, and in Kenyan adults who are exposed to malaria throughout the year. These studies did not identify any safety concerns regarding the candidate vaccine, which meant that it could progress further in clinical testing. As part of this next stage, a group of researchers wanted to examine the safety and ability of the vaccine to boost immune responses in an area of sub-Saharan Africa where people are not exposed to malaria throughout the year, but rather only in the wet season. The trial reported here was carried out in northeast Mali, in which 40 adults received either the FMP1/AS02A vaccine or a rabies vaccine for comparison, just at the start of the malaria transmission season. The researchers primarily looked at safety outcomes, collecting data on certain specific signs or symptoms up to 8 d after immunization, other reported symptoms up to 31 d after immunization, and any serious adverse events during a follow-up period of 364 d after immunization. The researchers also examined antibody levels in the participants' blood against the MSP-1 protein.

**What this trial shows:** The researchers found that participants receiving the FMP1/AS02A vaccine had more immediate symptoms at the injection site (for example, pain or swelling) than the comparison group did. Other general symptoms, both solicited and unsolicited, such as headache, muscle aches, fever, and infections, were also more common in the malaria vaccine group than in the group receiving the rabies vaccine. There were two serious adverse events in the vaccine group, but these were not judged to be related to the vaccination. Antibody levels against the MSP-1 protein increased in both study groups through the course of the rainy season (when individuals would be likely exposed to bites from malaria-infected mosquitoes) and subsequently fell after the end of the malaria transmission season. However, participants receiving the vaccine had higher antibody responses at all timepoints measured; the differences were statistically significant at some timepoints, but not at others. Finally, the researchers looked at antibody reactions against three different variants of the MSP-1 protein in sera from participants receiving the candidate vaccine and found that the sera reacted similarly to all three variants.

**Strengths and limitations:** The study protocol followed established procedures for phase I clinical trials of this type, which allows the data to be compared across studies. Randomization procedures were appropriate, and steps were taken to blind participants in the trial, as well as those assessing outcomes, to the intervention participants received. A limitation of this study, which can apply to other phase I studies in general, is that small numbers of participants were recruited. Therefore, the trial was not powered to detect statistically significant differences between participant groups. It is also not clear whether the higher antibody levels seen in the participants receiving the FMP1/AS02A vaccine would be biologically significant (that is, act to prevent clinical malaria cases), a question that would need to be addressed in further trials.

**Contribution to the evidence:** The safety results from this study are similar to those from other trials and confirm that no safety concerns have thus far been identified regarding the FMP1/AS02A vaccine, which has now progressed to efficacy testing. This study was also conducted in a population exposed to seasonal malaria, whereas previous trials had been done among people exposed to malaria year-round. Finally, results from the trial also suggest that this vaccine induces antibodies that recognize genetically diverse forms of the vaccine antigen.

## INTRODUCTION

An effective vaccine directed against the blood stages of Plasmodium falciparum malaria would prevent severe disease and death in African children and other at-risk populations. Merozoite surface protein-1 (MSP-1) is a 195-kDa antigen found on the surface of P. falciparum merozoites. It is essential for merozoite invasion of erythrocytes, after first being processed to yield a carboxy-terminal 42 kDa fragment (MSP-1_42_) that is further cleaved into the MSP-1_33_ and MSP-1_19_ fragments, as reviewed in [1]. MSP-1 has promise as a blood-stage malaria vaccine [2], but genetic polymorphism of the antigen [3,4] could limit vaccine efficacy if the protection conferred is allele specific.

Falciparum malaria protein 1 (FMP1) consists of recombinant MSP-1_42_ from the 3D7 clone of P. falciparum produced in and purified from Escherichia coli [5]*.* Reconstituted with the AS02A adjuvant, an oil-in-water formulation containing the immunostimulants monophosphoryl lipid A and QS21, it constitutes the FMP1/AS02A malaria vaccine. At the time of this study, this vaccine had been evaluated in two clinical trials evaluating a total of 60 malaria-naïve North American adults ([6] and D. G. Heppner, unpublished data), and in a further trial in 20 Kenyan adults exposed to intense year-round malaria transmission [7]. In these trials FMP1/AS02A was well tolerated and no safety concerns were identified.

The vaccine was highly immunogenic in malaria-naïve volunteers, inducing antibodies that recognized parasites by indirect fluorescent antibody (IFA), were reactive against recombinant fragments of MSP-1_42_ (including subdomains) by enzyme-linked immunosorbent assay (ELISA), and measurably inhibited parasite growth. Kenyan adults exposed to intense year-round malaria transmission had high and variable background levels of anti-MSP-1_42_ antibodies, and differences in antibody levels between malaria vaccine and control groups were not significant at any post-immunization timepoint; however, significant differences in antibody levels were measured when a longitudinal model was applied [7].

The safety, immunogenicity, and efficacy of malaria vaccines may be affected by the intensity and pattern of local malaria transmission, which determines levels of pre-existing natural immunity and potential natural “boosting” of the immune response to vaccines, and may affect the prevalence of different allelic parasite types. Western Kenya and Bandiagara, Mali, represent two sites with very different transmission patterns, both representative of other malaria-endemic areas where a vaccine would eventually be offered. We assessed the FMP1/AS02A malaria vaccine compared to rabies vaccine in adults living in Bandiagara, Mali, where malaria is highly seasonal and naturally acquired immunity is lower than in western Kenya. To investigate the specificity of vaccine-induced antibody responses, antibodies to diverse MSP-1_42_ alleles and subunits were measured.

## METHODS

### Study Setting

The Bandiagara Malaria Project research clinic is adjacent to the district hospital in Bandiagara, a rural town of 13,634 inhabitants in the Dogon Country in northeast Mali. It is relatively dry, with a mean annual rainfall of 600 mm in 2002. Anopheles gambiae is the principal malaria vector. Malaria transmission is strictly seasonal, with virtually undetectable transmission at the height of the dry season in March, less than one infected bite per person per month at the start and end of the transmission season in June and December, respectively, and peaks of up to 40–60 infected mosquito bites per person per month in August or September [8]. P. falciparum represents 97% of malaria infections, with 3% due to P. malariae and rare infections with P. ovale. Despite the seasonal transmission pattern, the malaria burden is heavy: each year, children aged under 10 y old have an average of two clinical malaria episodes every transmission season [8], and severe malaria afflicts 1 in 50 children aged under 6 y old [9]. Older children and adults are relatively protected against malaria disease, but remain susceptible to malaria infection.

### Participants

After obtaining community permission as described by Diallo et al. [10], the trial was publicized by local radio broadcast. Adults of both genders, aged 18–55 y, were invited to the research clinic to be screened for eligibility. Participants were included if they had resided in Bandiagara for at least 12 mo, gave written informed consent, and, if female, declared their intent not to become pregnant during the first 4 mo of the study. Exclusion criteria included current illness, previous immunization with a rabies vaccine, recent use of immunosuppressants, receipt of blood products during the previous 6 mo, pregnancy or breast-feeding, alcohol or drug abuse, and allergy to substances present in the vaccines.

### Interventions

The FMP1 antigen consists of the 42-kDa carboxy-terminal 392 amino acids of MSP-1 based on the sequence of the 3D7 clone of P. falciparum and 17 non-MSP-1 amino acids encoding a 6-histidine tag plus linking sequence fused to its amino terminus*.* The antigen was expressed as a fusion protein in E. coli with the 6-histidine tag to facilitate purification. The clinical grade antigen was manufactured according to current Good Manufacturing Practices (cGMP) at the Walter Reed Army Institute of Research Pilot Bioproduction facility (Silver Spring, Maryland, United States). The FMP1 protein met all purity, identity, and safety standards set for product release. After purification, less than 2 ng of host cell protein was detected per 50-μg dose. FMP1 protein was immunoreactive with several disulfide-dependent conformational monoclonal antibodies (mAbs), including growth inhibitory mAbs 12.10 and 12.8, implying correct structural conformation [5].

The AS02A adjuvant is composed of an oil-in-water emulsion and two immunostimulants, 3-deacylated monophosphoryl lipid A and QS21, a saponin agent derived from the soap bark tree, Quillaja saponaria [11,12]. AS02A was manufactured by GlaxoSmithKline Biologicals (Rixensart, Belgium) according to cGMP and provided in prefilled syringes. A single dose of FMP1/AS02A contained 50 μg of antigen dissolved in 0.5 mL of AS02A adjuvant immediately before injection.

The Imovax rabies vaccine (Aventis Pasteur, Swiftwater, Pennsylvania, United States) is a sterile preparation of killed rabies virus supplied in single-dose vials containing lyophilized antigen to which 1 mL of sterile water is added as a diluent before injection. Vaccines were administered by intramuscular injection in the left deltoid muscle.

Forty adults were randomized in a 1:1 ratio to receive either FMP1/AS02A or the control rabies vaccine. Vaccines were given on a 0-, 1-, and 2-mo schedule. The first immunization was given in early July 2003, just as malaria transmission began; the second dose was given at the end of July, as transmission was increasing; and the third dose was given in late August, near the peak of transmission intensity. Study day 90 was in October, shortly after transmission crests and when severe and uncomplicated malaria episodes peak; study day 180 was at the end of the malaria season; and study day 272 was in the middle of the dry season. The final study follow-up on day 364 coincided with the beginning of the 2004 malaria season. Interim safety reports were reviewed by an independent safety monitoring committee before the second and third immunizations.

### Objectives

The primary objective was to evaluate the safety and reactogenicity of the FMP1/AS02A malaria vaccine in malaria-experienced Malian adults. Secondary objectives were to evaluate the humoral immune response of the vaccine in malaria-experienced Malian adults in a setting of intense seasonal malaria transmission, to determine the impact of natural infection on malaria vaccine-induced antibody responses, and to measure antibody responses to genetically diverse forms of MSP-1_42_ and its subunits.

### Outcomes

The primary outcome was safety, measured as (1) occurrence of solicited symptoms during an 8-d follow-up period after immunization (day of immunization and days 1, 2, 3, and 7 after immunization); (2) occurrence of unsolicited symptoms during a 31-d follow-up period after immunization (day of immunization and 30 subsequent days); (3) occurrence of laboratory toxicities during the study period; and (4) occurrence of serious adverse events during the study period. Secondary outcomes were anti-MSP-1 antibody titers measured against recombinant MSP-1_42_ 3D7 and its subunits and two genetically different parasite strains (P. falciparum FVO and Camp/FUP), at baseline and at specified times during and after immunization.

#### Assessment of safety and tolerability.

Following each immunization, participants were directly observed for 30 min, then evaluated at the study clinic 1, 2, 3, 7, and 14 d after immunization and on study days 90, 180, 272, and 364. Starting on day 180, monthly home visits were made to check the health status of participants and to encourage them to come to the research clinic if they felt ill. Study physicians were available at all times throughout the 12-mo study to assess and treat adverse events.

Clinical evaluations consisted of measurement of vital signs and assessment for local injection site and general solicited signs or symptoms. Local signs and solicited symptoms included pain, swelling, erythema at the injection site, and limitation of arm abduction at the shoulder. General signs and solicited symptoms included fever (oral temperature ≥37.5 °C), chills, nausea, headache, malaise, myalgia, and joint pain. Any other signs or symptoms were considered to be unsolicited, as were signs or symptoms that occurred more than 7 d after immunization. Solicited symptoms were considered to be related to the study vaccines. Unsolicited signs and symptoms were recorded during the 30 d after each immunization, whereas serious adverse events were monitored throughout the 12-mo study.

Blood was collected at screening, on immunization days, 14 d after each immunization, and on study days 90, 180, 272, and 364 to determine complete blood count, alanine aminotransferase (ALT), and serum creatinine.

Adverse events were graded by severity and judged for relatedness to study vaccines. Mild adverse events were easily tolerated, causing minimal discomfort and not interfering with daily activities. Moderate adverse events were sufficiently discomforting to interfere with normal activities. Severe adverse events prevented normal daily activities. Swelling, erythema, fever, and limitation of arm motion had specific definitions not based on interference with daily activities. Injection site swelling and erythema were graded based on their widest dimension: mild, >0–20 mm; moderate, >20–50 mm; and severe, >50 mm. Fever was classified as severe if the oral temperature was ≥39 °C , whereas severe limitation of arm motion was classified as abduction limited to 30°. For laboratory tests, toxicity grading was adapted to normal reference ranges determined for the local adult population.

#### Antibody responses to MSP-1_42_.

Antibody responses to MSP-1_42_ were measured by ELISA [6]. Briefly, cGMP-purified bulk MSP-1_42_ [3] was used as plate antigen, and serial dilutions of each sample, along with positive and negative controls, were made to yield a linear range of dilutions that could be analyzed with curve-fitting software (SoftMax Pro v4.1, Molecular Devices, Sunnyvale, California, United States) to calculate the theoretical dilution that would give an optical density of 1.0 in the endpoint assay; the reciprocals of these calculated dilutions were reported as the sample titers. To compare antibody responses to different alleles of MSP-1_42_, recombinant MSP-1_42_ of the 3D7 and FVO alleles were prepared as described previously [6,13]. Preparation of recombinant MSP-1_42_ of the Camp/FUP allele will be described elsewhere.

#### Antibody responses to fragments of MSP-1_42_.

MSP-1 fragment-specific antibody responses were assessed by a standard ELISA [6]. Fragments of MSP-1_42_ corresponding to MSP-1_19_ (3D7 and FVO alleles) and the two epidermal growth factor (EGF)-like domains that comprise MSP-1_19,_ EGF1 (3D7-Camp/FUP [14] and FVO alleles) and EGF2 (3D7 and FVO-Camp/FUP alleles), were expressed as glutathione S-transferase fusion proteins and purified to homogeneity [13].

### Sample Size

This phase I trial was not powered to detect differences between groups. A sample size of 20 malaria vaccine recipients was based on general acceptance of this size for initial assessment of safety, tolerability, and immunogenicity of investigational vaccines. Inclusion of a comparator vaccine group of 20 permitted broad estimates of the incidence of local and general side effects and immune responses to natural infection.

### Randomization

Randomization was done in blocks of four without stratification. Opaque, sealed randomization envelopes containing sequential codes linked to vaccine assignment were prepared by Statistics Collaborative (Washington, D. C., United States). Codes were assigned in the order that participants arrived at the clinic on the day of first immunization.

### Blinding

The only people at the study site with access to the randomization codes during the study were two study pharmacists, who had no contact with study participants and did not reveal vaccine assignments to anyone else. Study participants and investigators who assessed outcomes were blinded to vaccine assignment. Vaccines were prepared in a secure room communicating with the vaccine administration room through a small window with a sliding door. Reconstituted FMP1/AS02A is off-white and Imovax is pink. To reduce potential bias, syringes containing the vaccines were wrapped with opaque tape to conceal their contents from participants and vaccinators. Vaccines were administered by physicians who did not participate in assessing outcomes.

### Ethical Compliance

The protocol was approved by institutional review boards of the University of Bamako Faculty of Medicine, the University of Maryland, the United States Army Surgeon General, and the National Institute of Allergy and Infectious Disease. Separate written informed consent was obtained for screening and for enrollment. Consent of illiterate participants was documented by their thumbprints and by signatures of independent witnesses. Permission to conduct the study was granted by the Republic of Mali Ministry of Health, and the trial was monitored by the United States Army Medical Materiel Development Activity and the World Health Organization.

### Statistical Methods

Adverse event rates were analyzed using SAS version 8.2 (SAS Institute, Cary, North Carolina, United States). Fisher's exact test was used to compare rates between vaccine groups. Confidence intervals (CIs) for geometric mean MSP-1_42_ antibody titers were estimated by using log_10_-transformed values, calculating the 95% CI based on the normal distribution, and then converting the limits to the original scale for presentation. All tests were two-sided, and no correction of *p*-values was made for additional analyses. MSP-1_42_ fragment-specific antibody responses were measured longitudinally and are reported as the median slope of the regression lines for responses for the malaria vaccine and control groups. Comparisons between groups were made using Mann–Whitney tests or Student's t test of log_10_-transformed antibody titers.

## RESULTS

### Participant Flow and Baseline Data

**Figure 1:**
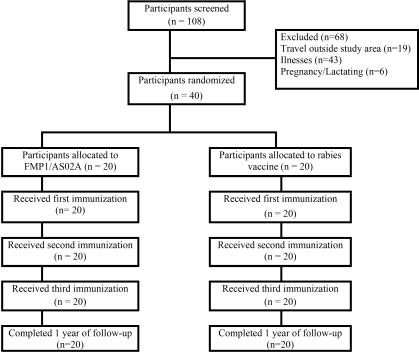
Trial Profile

Of 108 screened adults, 40 were deemed eligible and enrolled in July 2003 (Figure 1). The main reasons for exclusion were concurrent illnesses and intent to travel during the trial. The two vaccine groups did not differ significantly at enrollment with regard to sex, age, or laboratory parameters (Table 1). Seven participants were female. The mean age was 39 y. All participants received all three immunizations according to the 0-, 1-, and 2-mo schedule. All participants completed all scheduled visits and were included in the analysis.

**Table 1:**
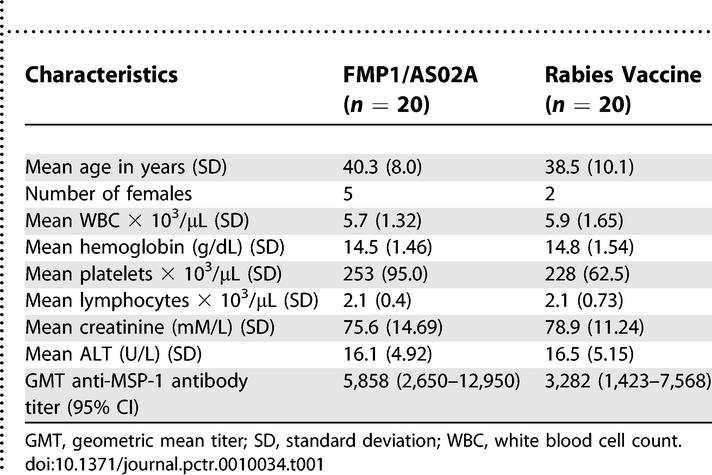
Baseline Characteristics of FMP1/AS02A Malaria Vaccine and Rabies Vaccine Groups

### Safety and Reactogenicity

#### Local solicited adverse events.

After each immunization, the proportion of participants who had at least one local injection site reaction during the 8-d post-immunization periods was not significantly different between vaccine groups. Pain and swelling at the injection site were the most common local reactions for both groups and tended to be more common in the FMP1/AS02A group and after the first immunization (Table 2).

**Table 2:**
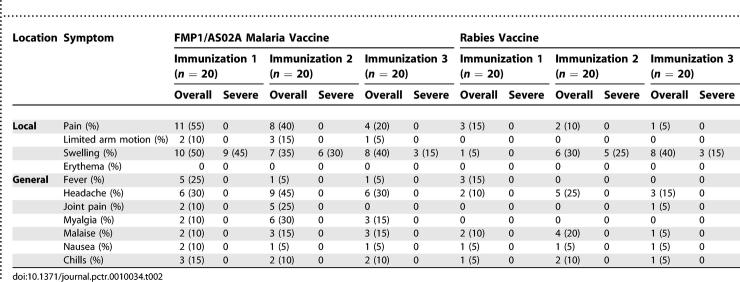
Signs and Solicited Symptoms during the 8-d Follow-Up Periods after Each Immunization

After the first immunization, nine recipients of FMP1/AS02A and none of the rabies vaccine recipients had at least one instance of severe injection site swelling (*p* = 0.001). No other severe reactions were observed. Severe local swelling was less common after the second and third immunizations and was similar in frequency between groups. Severe injection site swelling occurred in 13 FMP1/AS02A recipients across all immunizations (in nine, six, and three participants after the first, second, and third immunization, respectively) and in eight rabies vaccine recipients across all immunizations (in zero, five, and three participants after the first, second, and third immunizations, respectively) (Table 2). The swelling was typically unnoticed by the participant and detected only on physical examination and did not interfere with normal daily activities. No other severe local adverse events occurred. Six episodes of arm motion limitation were reported among FMP1/AS02A recipients: one mild episode after the first immunization; and two moderate and one mild episode after the second immunization. All local solicited symptoms resolved without sequelae during the 8-d post-immunization periods.

#### General solicited adverse events.

The FMP1/AS02A group had a higher proportion of participants who had at least one general solicited sign or symptom during the 8-d post-immunization periods (19/20 FMP1/AS02A recipients versus 12/20 rabies vaccine recipients; *p* = 0.02). Headache was the most common general adverse event in FMP1/AS02A recipients after each immunization, followed by myalgia and malaise (Table 2). All general solicited adverse events were of mild or moderate intensity and ended during the 8-d follow-up period.

#### Unsolicited adverse events.

The most common nonserious, unsolicited adverse events were subjective fever, infections, and rhinitis. During the three 31-d post-immunization periods, 13 participants in the FMP1/AS02A group and seven in the control group had subjective fever at least once. One or more genitourinary and lower respiratory infections occurred in nine FMP1/AS02A recipients and in five rabies vaccine recipients in the same periods. Rhinitis was reported by eight participants in the FMP1/AS02A group and by six in the rabies group. Only one severe unsolicited adverse event, a urinary tract infection in a female FMP1/AS02A recipient, occurred during the 31-d post-immunization periods. These unsolicited events were not temporally associated with immunization and were not considered to be related to vaccination. The incidence of unsolicited adverse events was not significantly different between the two study groups.

#### Serious adverse events.

Two serious adverse events occurred during the study, both in the FMP1/AS02A group. One participant developed sinusitis requiring hospitalization between the second and third immunizations, and the other had a tubal ectopic pregnancy a month after her third immunization. Both participants recovered fully, and both events were judged to be unrelated to immunization.

#### Laboratory safety tests.

Mildly elevated serum creatinine levels were detected in seven participants (three in the FMP1/AS02A group and four in the control group). These were not associated with other clinical abnormalities or illness and resolved without intervention. Two participants in the control group had elevated ALT levels. One was a moderate elevation to 131 U/L detected during an acute malaria episode on day 272 that returned to normal by day 364. The second was a severe elevation to 359 U/L unaccompanied by symptoms or signs of hepatitis or other illness that was detected on day 74 and resolved by day 272. Serological tests for hepatitis A, B, and C were negative. The participant had been treated with erythromycin for a respiratory infection and was given a diagnosis of drug-induced hepatitis.

Hemoglobin levels remained within or slightly above the normal range for all participants throughout the study (11.7–17.3 g/dL for males; 10.0–14.4 g/dL for females). Mild abnormalities in white blood cell and platelet counts were infrequent and balanced by group.

### Immunogenicity

**Figure 2:**
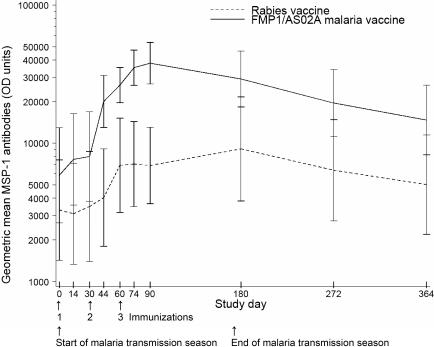
Anti-MSP-1_42_ Antibody Titers Geometric mean antibody titers to homologous recombinant MSP-1_42_ for FMP1/AS02A vaccine (solid line) and control rabies vaccine (dotted line) recipients. Times of each of three immunizations and the start and end of the malaria transmission season are indicated by arrows. Bars represent 95% CI.

In the rabies vaccine group, anti-MSP-1_42_ antibodies rose over the course of the malaria season, increasing more than 2-fold by day 60 and peaking at nearly 3-fold above baseline responses on day 180 as the malaria season ended (Figure 2; Table 3). Titers then decreased throughout the dry season and returned to near baseline levels by the start of the subsequent rainy season. In the FMP1/AS02A vaccine recipients, although the shape of the curve is similar, the magnitude of the response was significantly greater, with a 6.5-fold increase between day 0 and day 90, 30 d after the third immunization. At day 90, the geometric mean titers were 37,923 in the FMP1/AS02A recipients and 6,892 in the comparator group (*p* < 0.001), and at day 180, they were 29,150 and 9,089 (*p* = 0.016). By the final follow-up timepoint 1 y after immunization, although titers had waned in both groups, they remained significantly higher in the FMP1/AS02A group (14,693 compared to 5,015 in the control group; *p* = 0.032).

**Table 3:**
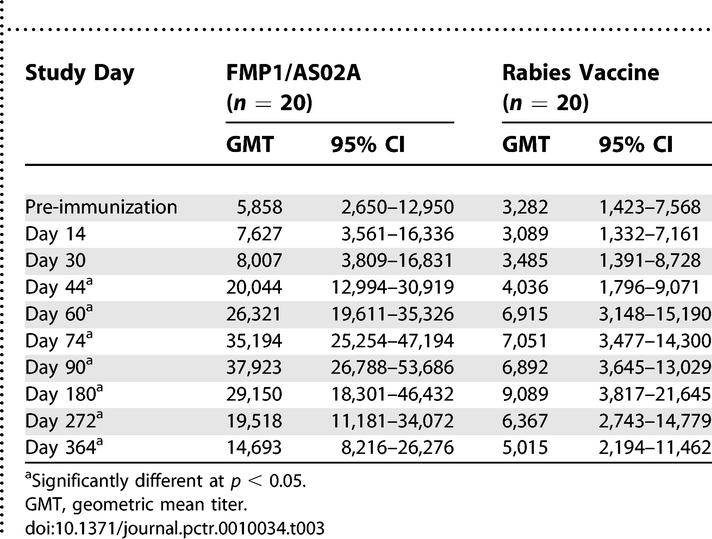
Geometric Mean Anti-MSP-142 Antibody Titers

Individual responses to the vaccine varied. Of the 20 participants who received FMP1/AS02A, six developed very high antibody titers, with an 8-fold or greater rise in day-90 antibody titers compared to baseline; six had a 4- to 7-fold rise; four had a 2- to 3-fold rise; and four had a <2-fold rise. Much of this individual variation can be explained by the variation in background immunity at the start of the malaria transmission season: the baseline geometric mean MSP-1_42_ titer was <3,000 in the six participants with an 8-fold or greater rise in titer, but >20,000 in those with a <2-fold rise.

### Fragment- and Allele-Specific Anti-MSP-1_42_ Antibody Responses

**Figure 3:**
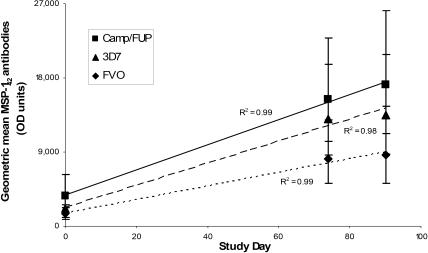
Allele-Specific Anti-MSP-1_42_ Antibody Titers Linear regression of point-wise geometric mean titers against the three allelic forms of MSP-1_42_ measured at study days 0, 74, and 90. Bars represent 95% CI.

Timepoints for measurement of the rates of acquisition of MSP1 fragment-specific antibodies in this adult population with significant baseline antibodies were chosen from the observed maxima for antibody to the entire MSP-1_42_, i.e., days 74 and 90. Sera collected on study day 0 were analyzed to provide paired baseline data. Responses were measured by ELISA against three alleles of MSP-1_42_ (3D7, Camp/FUP, and FVO) and subunit fragments of MSP-1_42_ from both 3D7 and FVO (MSP-1_42_, MSP-1_19_, EGF-1, and EGF-2). Antibody responses induced by FMP1/AS02A against the three alleles did not differ significantly (Figure 3), although there was a tendency toward greater recognition of the 3D7 and Camp/FUP alleles compared to the FVO allele (*p* = 0.08 and 0.064, respectively). For all comparisons, slopes for anti-MSP-1_42_ and subunit antibodies were significantly higher in the FMP1/AS02A group than in the rabies control group (Table 4).

**Table 4:**
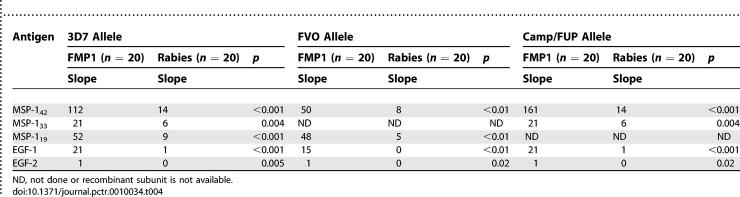
Median Slope of the Regression Line for Day 0, 74, and 90 Antibody Responses to 3D7 (Homologous to the FMP1 Vaccine Antigen) and FVO Allelic Types of MSP-1 42-kDa, 33-kDa, 19-kDa, EGF-1, and EGF-2 Subunits

## DISCUSSION

### Interpretation

The P. falciparum blood-stage vaccine FMP1/AS02A was safe, well tolerated, and highly immunogenic against diverse parasite clones in adults with lifelong exposure to intense seasonal malaria transmission in Bandiagara, Mali.

As in two previous phase I trials of FMP1/AS02A [6,7], the vaccine had acceptable tolerability, with most reactogenicity occurring after the first of the three immunizations and decreasing reactogenicity after the second and third immunizations. In general, we observed more solicited adverse events in the FMP1/AS02A group when compared to the rabies group, with the most common reactions being short-lived swelling and mild pain at the injection site. Although swelling was often classified as severe based on the size of the reaction, these episodes were well tolerated and were usually unnoticed by participants. No participants were withdrawn from the study because of adverse events. No serious adverse event related to the vaccine was observed. No clinically significant laboratory abnormality related to the vaccine occurred. Hemoglobin levels remained at or above normal ranges for all participants during the study period.

Immunization with FMP1/AS02A led to increased antibody titers against MSP-1_42_, which peaked with a 6.5-fold rise over baseline 1 mo after the third immunization. A boosting effect was seen after each of three immunizations, in contrast to a trial of this vaccine in malaria-naïve North Americans [6], in which the third immunization did not lead to a significant additional rise in titer.

### Generalizability

While cellular immune responses, antibody avidity assays, growth inhibition assays, and other potential alternative surrogate markers may eventually prove more predictive of clinical protection than MSP-1_42_ antibody titers, the validity of these assays as endpoints for clinical trials will need to be demonstrated in appropriate target populations (e.g., infants and children in malaria-endemic areas) in which protective efficacy is demonstrated. For this small phase I trial in malaria-experienced adults, we chose to use MSP-1_42_ antibody titers as the immunogenicity endpoint to inform clinical development decisions.

### Overall Evidence

Immunizing at the start of the of the malaria transmission season permitted observation of the effect of natural boosting on MSP-1_42_ antibody titers in the control group, which rose nearly 3-fold over the malaria season. The difference in MSP-1_42_ antibody levels between the FMP1/AS02A and rabies vaccine groups was much greater in this trial than in a virtually identical trial conducted in an area of intense year-round malaria transmission in Kenya [7]. The same reference laboratory measured antibody titers in both trials using the same methods, ruling out methodological reasons for the different immunogenicity results. Most of this difference can be attributed to the lower baseline antibody levels at the beginning of the malaria transmission season in Mali: in the FMP1/AS02A vaccine group, antibody titers rose from <6,000 to a peak of nearly 38,000 in Mali, compared to an increase from 17,000 to 46,000 in Kenya [7]. This conclusion is supported by the observation that the rates of increase in vaccine-induced antibody responses to the 3D7 alleles of MSP-1_42_ and MSP-1_19_, as measured by the slopes of the regression lines fitted using data from day 0, 74, and 90, were not different between the two study sites (data not shown).

The vaccine induced responses against all alleles tested: 3D7 (homologous to the vaccine antigen), Camp/FUP (different in three amino acids in the second EFG-like domain), and FVO (different in four amino acids in MSP-1_19_, and the most divergent in MSP-1_33_). Longitudinal antibody responses showed a tendency toward stronger reactivity against the 3D7 and Camp/FUP alleles than against the FVO allele, which is consistent with the greater degree of homology between 3D7 and Camp/FUP than between 3D7 and FVO. Whether these in vitro allele-specific analyses predict in vivo efficacy against diverse parasites is not yet known.

The dramatically different immune responses to FMP1/AS02A seen in Mali and Kenya highlight the need to assess malaria vaccines in different transmission settings early in their development. Conceivably, high background immunity could obscure vaccine-induced immunity to the extent that a potentially efficacious vaccine tested only in high transmission settings would be prematurely abandoned. Settings with seasonal transmission offer the advantages of relatively lower background immunity yet high clinical attack rates [8] that make them particularly suitable for phase I and II malaria vaccine trials.

In a recent trial in Mozambique, the pre-erythrocytic malaria vaccine RTS,S/AS02A provided significant, sustained protection against both severe and uncomplicated malaria [15,16]. Presently, a randomized, placebo-controlled efficacy trial of FMP1/AS02A is underway in 400 Kenyan children 1–4 y of age. If clinical protection is demonstrated, FMP1/AS02A may have the potential for development as a vaccine and/or to enhance the protection conferred by RTS,S in a multi-antigen, multistage malaria vaccine [17].

## SUPPORTING INFORMATION

CONSORT Checklist Click here for additional data file. (49 KB DOC)

Trial Protocol Click here for additional data file. (376 KB PDF)

## Abbreviations

ALT: alanine aminotransferase
cGMP: current Good Manufacturing Practices
CI: confidence interval
EGF: epidermal growth factor
ELISA: enzyme-linked immunosorbent assay
FMP1: falciparum malaria protein-1
IFA: indirect fluorescent antibody
mAb: monoclonal antibody
MSP-1: merozoite surface protein-1
