# Supplementary material for: Safety and Allele-Specific Immunogenicity of a Malaria Vaccine in Malian Adults: Results of a Phase I Randomized Trial
Source: PLoS Clin Trials. 2006 Nov 24;1(7):e34. doi: 10.1371/journal.pctr.0010034 (PMC1851722; doi:10.1371/journal.pctr.0010034)
Supplement: Trial Protocol [file pctr.0010034.sd002.pdf]

TITLE OF PROJECT: Double blind randomized controlled Phase I trial to evaluate the safety and immunogenicity of WRAIR's MSP1 candidate malaria vaccine (FMP1) adjuvanted in GlaxoSmithKline Biologicals' AS02A vs. Rabies vaccine in semi-immune adults in Bandiagara, Mali

**Study vaccine(s)**	Walter Reed Army Institute of Research' candidate *Plasmodium falciparum* malaria MSP1 vaccine adjuvanted with GlaxoSmithKline Biologicals' AS02A
**Trial site**	Bandiagara, Mali, Tel: 223-2-420-495
**DMID Protocol No.**	02-184
**UMB IRB Protocol No.**	0303311
**HSRRB Study Log No.**	1029
**NIAID IRB No.**	03-I-N206
**MMVDU Protocol No.**	Malaria 001
**GSKBiologicals Protocol No.**	CPMS 257049/032
**IND No.**	9202
**Version**	XIX, June 2, 2003
**Title**	Double blind randomized controlled Phase I trial to evaluate the safety and immunogenicity of WRAIR's MSP1 candidate malaria vaccine (FMP1) adjuvanted in GlaxoSmithKline Biologicals' AS02A vs. Rabies vaccine in semi-immune adults in Bandiagara, Mali

IND Sponsor:

Office of the Surgeon General, U.S. Army
The U.S. Army Medical Materiel Development Activity
Product Manager: Dr. Charles English
622 Neiman Street
Fort Detrick, Maryland 21702-5009
Tel: 301- 619-2136

Study Co-Sponsor:

Division of Microbiology & Infectious Diseases, NIAID, NIH

Contact : Abdi Naficy M.D., M.P.H.
Medical Officer
Parasitology & International Programs Branch
Division of Microbiology & Infectious Diseases
National Institute of Allergy & Infectious Diseases
National Institutes of Health
6610 Rockledge Drive, MSC 6603

Bethesda, MD 20892, USA
Tel: (301) 435 2866
Fax: (301) 402 0659
E-mail: anaficy@niaid.nih.gov

Principal Investigator:
Mahamadou A. Thera M.D., M.P.H.
Malaria Research and Training Center
Department of Epidemiology of Parasitic Diseases
Faculty of Medicine, Pharmacy and Dentistry, University of Mali
BP 1805, Point G
Bamako, Mali, West Africa
Tel/Fax 223-222-8109
E-mail: mthera@mrtcbko.org

Co-principal Investigator:
Christopher V. Plowe M.D. M.P.H.
Center for Vaccine Development
University of Maryland School of Medicine
685 West Baltimore Street, HSF 480
Baltimore, Maryland 21201 USA
Tel 410-706-3082
Fax 410-706-6205
E-mail: cplowe@medicine.umaryland.edu

Co-Investigators:
MRTC Mali
Ogobara K. Doumbo M.D., Ph.D. (Senior co-investigator)
Dapa A. Diallo M.D.
Alassane Dicko M.D., M.Sc.
Drissa Coulibaly M.D.
Abdoulaye Koné M.D.
Ando Guindo M.D.
Mohammed B. Niambélé M.D.
Moussa Sogoba M.D.
Mounirou Baby Pharm.D.
Karim Traoré, M.D.
Modibo Daou, Pharm.D.Issa Diarra, Pharm.D.
Yacouba Cissoko, M.D.
Amagana Dolo, Pharm.D., Ph.D.
Malaria Research and Training Center
Department of Epidemiology of Parasitic Diseases
Faculty of Medicine, Pharmacy and Dentistry, University of Mali
BP 1805, Point G
Bamako, Mali, West Africa
Tel/Fax: + 223-222 81 09; 221 63 61; 222 49 87

CVD Maryland
Kirsten E. Lyke M.D.
Marcelo Sztein M.D.
Aric Gregson M.D.
Karen Kotloff, M.D.
Robert Edelman (Consultant)

Myron M. Levine, M.D. (Consultant)
Center for Vaccine Development
University of Maryland School of Medicine
685 West Baltimore Street, HSF 480
Baltimore, Maryland 21201 USA
Tel 410-706-5328
Fax 410-706-6205

MVDU NIH

David Diemert M.D.
Elissa Malkin, D.O., M.P.H.
Louis H. Miller M.D. (Consultant)
Malaria Vaccine Development Unit
National Institute of Allergy and Infectious Diseases
National Institutes of Health
5640 Fishers Lane
Twinbrook I, Room 1125
Rockville, Maryland 20852 USA
Tel 301-443-3915
Fax 301-435-6725

WRAIR

D. Gray Heppner M.D.
Christian F. Ockenhouse, M.D., Ph.D.
Kurt Schaecher, Ph.D.
Kent E. Kester, M.D.
Jeff Lyon, Ph.D.
Evelina Angov, Ph.D.

Department of Immunology
Walter Reed Army Institute of Research
Tel 301-319-9414
Fax 301-319-7358

USAID

Carter Diggs, M.D., Ph.D.
Tel 202-712-5728
Lorraine Soisson, Ph.D
Tel 908-369-7704

GSKBio

Amanda Leach, M.S.c, MRCPCH
Clinical Development Manager
GSK Biologicals
Tel: +32-2-656-7788
Fax: +32-2-656-6160
Alfred Tiono, M.D.
WHO/GSK Biologicals Trainee
GSK Biologicals
Tel: +32-2-656-6535
Fax: +32-2-656-6160

**Local Medical Monitor (Sponsor's Medical Expert)**	Prof. Hamar Alassane Traoré<br>Hopital Point G, BP 1805, Bamako, Mali<br>Tel 223 222 5002
**Clinical Trial Coordinator**	Drissa Coulibaly, M.D.<br>Malaria Research and Training Center<br>Department of Epidemiology of Parasitic Diseases<br>Faculty of Medicine, Pharmacy and Dentistry, University of Mali<br>BP 1805, Point G<br>Bamako, Mali, West Africa<br>Tel/Fax: + 223-222 81 09; 221 63 61
**Data Quality Manager**	Issaka Sagara, M.D., M.Sc.<br>Malaria Research and Training Center<br>Department of Epidemiology of Parasitic Diseases<br>Faculty of Medicine, Pharmacy and Dentistry, University of Mali<br>BP 1805, Point G<br>Bamako, Mali, West Africa<br>Tel/Fax: + 223-222 81 09; 221 63 61; 222 49 87
**Regulatory Affairs Specialist:**	Linda L. Rosendorf, M.S,<br>Center for Vaccine Development<br>University of Maryland<br>685 West Baltimore Ave.<br>Baltimore MD 21201<br>phone: 410-706-0431<br>fax: 410-706-4171<br>e-mail:lrosendo@medicine.umaryland.edu
**Statistical Consultant:**	Janet Wittes, Ph.D.<br>Statistics Collaborative<br>1710 Rhode Island Avenue NW, Suite 200<br>Washington DC 20036 USA<br>Tel 202-429-9267<br>Fax 202-429-9268
**Clinical laboratory to be used**	Bandiagara Malaria Project clinical laboratory
**Study Monitors:**	Quality Assurance Office, USAMMDA<br>Division of Microbiology and Infectious Diseases<br>GlaxoSmithKline<br>World Health Organization/Tropical Disease Research

APPROVED AT MALI MALARIA VACCINE DEVELOPMENT UNIT/MALARIA RESEARCH AND TRAINING CENTER BY:

Ogobara Doumbo, M.D., Ph.D.
Director of MRTC

I, the undersigned, have reviewed this protocol, including Appendices and I will conduct the clinical study as described and will adhere to the ICH/cGCP and applicable Regulatory requirements. I have read and understood the contents of the Investigator Brochure provided by WRAIR.

Dr. Mahamadou A. Thera	____________________	______________
Principal Investigator	Signature	Date

TITLE OF PROJECT: Double blind randomized controlled Phase I trial to evaluate the safety and immunogenicity of WRAIR's MSP1 candidate malaria vaccine (FMP1) adjuvanted in GlaxoSmithKline Biologicals' AS02A vs. Rabies vaccine in semi-immune adults in Bandiagara, Mali 1
1 LIST OF ABBREVIATIONS 9
2 GLOSSARY OF TERMS 10
3 SUMMARY 11
4 INTRODUCTION AND BACKGROUND 12
4.1 MALARIA PARASITE LIFE CYCLE 12
4.2 DISEASE BURDEN 12
4.2.1 Malaria in Mali 13
4.3 RATIONALE FOR A MALARIA VACCINE: 14
4.3.1 Clinical Experience with the AS02A Adjuvant 15
4.3.2 The FMP1 Vaccine 17
4.3.3 Pre-clinical Toxicity, Safety and Reactogenicity of the FMP1/AS02 Malaria Vaccine 17
4.3.4 Clinical Experience with FMP1/AS02A 17
4.3.5 Justification of 0, 1, 2 Month Schedule 19
4.3.6 Comparison Vaccine 19
4.3.7 Safety of Imovax® Rabies vaccine 20
4.3.8 Rationale for double blind controlled design 21
4.3.9 Justification for conducting FMP1 trial in Mali 21
5 OBJECTIVES 21
5.1 PRIMARY OBJECTIVE 21
5.2 SECONDARY OBJECTIVE 21
6 STUDY DESIGN AND METHODOLOGY 22
6.1 OVERVIEW 22
6.2 NUMBER OF PARTICIPANTS/CENTER 22
6.3 ROLES AND RESPONSIBILITIES OF KEY STUDY PERSONNEL 23
6.4 INCLUSION CRITERIA 25
6.5 EXCLUSION CRITERIA 25
6.5.1 Justification for the Exclusion of Children 26
6.5.2 Rationale for using clinical assessment of immunosuppression 26
6.6 TREATMENTS THAT COULD POTENTIALLY INTERFERE WITH THE VACCINE-INDUCED IMMUNITY 27
6.7 CONTRAINDICATIONS TO VACCINATION 27
6.8 INDICATIONS FOR DEFERRAL OF IMMUNIZATION 27
7 CONDUCT OF THE STUDY 28
7.1 GENERAL STUDY ASPECTS 28
7.1.1 Screening and inclusion process 28
7.1.2 Vaccination process 28
7.1.3 Procedures to be followed for post-immunization evaluations 29
7.1.4 Competencies of staff 29
7.1.5 Detailed description of study visits 30
7.1.6 Outline of study procedures 34
7.2 DEFINITION AND MANAGEMENT OF CLINICAL MALARIA 34
7.2.1 Definition of clinical malaria 34
7.2.2 Treatment of malaria clinical episodes during the study 35
8 SAMPLE HANDLING AND ANALYSIS 36

8.1 OVERVIEW OF COLLECTION TIME POINTS 36
8.2 HANDLING OF BIOLOGICAL SAMPLES COLLECTED BY THE INVESTIGATORS 36
8.2.1 Instructions for handling of serum samples 36
8.2.2 Labeling 37
8.3 LABORATORY ASSAYS 37
Additional assays 38
9 STUDY VACCINES/MEDICATIONS AND ADMINISTRATION 38
9.1 STUDY VACCINES 38
9.1.1 FMP1 vaccine 38
9.1.2 Imovax® Rabies vaccine 39
9.2 DOSAGE AND ADMINISTRATION 39
9.2.1 Reconstitution of FMP1 vaccine 39
9.2.2 Reconstitution of Imovax ® Rabies vaccine 39
9.2.3 Administration of vaccines 39
9.3 STORAGE 40
9.4 TREATMENT ALLOCATION AND RANDOMIZATION 40
9.5 METHODS OF BLINDING AND BREAKING THE STUDY BLIND 40
9.5.1 Blinding 40
9.5.2 Breaking the study blind 41
9.6 REPLACEMENT OF UNUSABLE VACCINE DOSES 42
9.7 VACCINE ACCOUNTABILITY 42
9.8 CONCOMITANT MEDICATION/TREATMENT 42
9.8.1 Drugs to treat anaphylaxis 42
9.8.2 Drugs to treat malaria 43
10 ADVERSE EVENTS 43
10.1 ELICITING AND DOCUMENTING ADVERSE EVENTS 43
10.1.1 Adverse event definition 43
10.1.2 Surveillance period for occurrence of adverse events 44
10.1.3 Recording adverse events 44
10.1.4 Solicited adverse events 44
10.1.5 Unsolicited adverse events 45
10.2 ASSESSMENT OF INTENSITY 45
10.3 ASSESSMENT OF CAUSALITY 46
10.4 FOLLOWING-UP OF ADVERSE EVENTS AND ASSESSMENT OF OUTCOME 47
10.5 SERIOUS ADVERSE EVENTS 48
10.5.1 Definition of a serious adverse event 48
10.5.2 Reporting serious adverse events 48
10.6 PREGNANCY 50
10.7 TREATMENT OF ADVERSE EVENTS 50
11 PARTICIPANT COMPLETION AND DROP-OUT 50
11.1 DEFINITION 50
11.2 PROCEDURE FOR HANDLING DROP-OUTS 51
11.3 REASONS FOR DROP-OUT 51
12 DATA MANAGEMENT AND ANALYSIS 51
12.1 PRIMARY ENDPOINTS 51
12.2 SECONDARY ENDPOINTS 51
12.3 STUDY COHORTS/DATASETS TO BE EVALUATED 52
12.3.1 Total cohort 52
12.3.2 Safety cohort 52
12.3.3 Immunogenicity cohort 52

12.4 ESTIMATED SAMPLE SIZE 52
12.5 FINAL ANALYSIS 53
12.5.1 Analysis of demographics 53
12.5.2 Analysis of immunogenicity 53
12.5.3 Analysis of safety 53
12.6 PRELIMINARY ANALYSIS 54
12.7 ADMINISTRATIVE MATTERS 54
13 TIME FRAME 54
14 ETHICAL CONSIDERATIONS 54
14.1 STATEMENT OF COMPLIANCE AND ETHICAL REVIEWS 54
14.1.1 Institutional Review Boards (IRB) 55
14.2 INFORMED CONSENT 55
14.3 ROLE OF LOCAL MEDICAL MONITOR (LOCAL SAFETY MONITOR) 55
14.4 SAFETY MONITORING COMMITTEE (SMC) 56
14.5 DATA SAFETY MONITORING BOARD (DSMB) 57
14.5.1 Composition of the DSMB 57
14.5.2 Role of the DSMB 57
14.6 NIAID DSMB 58
14.7 RISKS AND POTENTIAL BENEFITS TO THE PARTICIPANTS 59
14.7.1 Vaccination 59
14.7.2 Medical treatment for participants 59
14.7.3 Rabies vaccination 59
14.7.4 Risks associated with malaria treatment 60
14.7.5 Pregnancy 60
14.7.6 Benefits 60
14.8 PRECAUTIONS TO MINIMIZE RISKS 61
14.8.1 Vaccination 61
14.8.2 Malaria treatment 61
14.8.3 Protection of study staff 61
14.9 PROCEDURES FOR MAINTAINING CONFIDENTIALITY 61
14.10 COMPENSATION 61
14.11 FINANCING AND INSURANCE 62
15 REFERENCES 62
16 APPENDICES 68
16.1 SCREENING AND STUDY INFORMED CONSENT 68
16.2 SCREENING RECRUITMENT RADIO ANNOUNCEMENT TEXT 69
16.3 USAMMDA REPORTING SCHEME OF SAEs 70
16.4 ADMINISTRATIVE MATTERS 71
16.5 USAMRMC SPECIFIC ADMINISTRATIVE PROCEDURES 73

1 LIST OF ABBREVIATIONS

®:	Registered trademark
3D7:	Clone of *Plasmodium falciparum*
ALT:	Alanine aminotransferase
AST:	Aspartate aminotransferase
AS02:	Adjuvant System 2 of Glaxo SmithKline with thiomersal
AS02A:	Adjuvant System 2 of Glaxo SmithKline without thiomersal
CBER:	Center for Biologics Evaluation and Research
CBC:	Complete blood count
CMI:	Cell mediated immunity
CRF:	Case Report Form
CS gene:	Circumsporozoite gene of *P. falciparum*
CSP:	Circumsporozoite protein
CTL:	Cytotoxic T lymphocyte
CVD:	Center for Vaccine Development, University of Maryland Baltimore
DMID:	Division of Microbiology & Infectious Diseases, U.S. National Institutes of Health
DSMB:	Data Safety and Monitoring Board
DNA:	Deoxyribonucleic acid
EGF:	Epidermal Growth Factor
EIR:	Entomologic inoculation rate
ELISA:	Enzyme linked immunosorbent assay
Elispot:	Method for the detection of antibody/cytokine-secreting cells
FMP1:	Falciparum Malaria Protein 1
GSK:	Glaxo SmithKline
GMT:	Geometric Mean Titer
HBsAg:	Hepatitis B surface antigen
HLA:	Human leukocyte antigen
HSRRB:	Human Subjects Research Review Board
ICH:	International Conference on Harmonization
ID:	Identification
IEC:	Institutional Ethical Committee
IFA:	Indirect fluorescent antibody
IM:	Intramuscular
IRB:	Institutional Review Board (Ethical Review Committee)
MPL:	Monophosphoryl Lipid A
3D-MPL	3-deacylated Monophosphoryl Lipid A
MRTC:	Malaria Research and Training Center
MMVDU:	Mali Malaria Vaccine Development Unit
MVDU:	Malaria Vaccine Development Unit, U.S. National Institutes of Health
NIAID:	National Institute of Allergy and Infectious Diseases
NANP:	Repeat epitopes of the circumsporozoite protein
PBMC:	Peripheral blood mononuclear cells
PCR:	Polymerase chain reaction
PID	Participant Identification Number
QS21:	*Quillaja saponaria* 21 (saponin derivative)
RIA	Radioimmunoassay
RTS:	Fusion protein between circumsporozoite protein based antigen and HBsAg

S:	226 amino acid polypeptide corresponding to the surface antigen of hepatitis B virus (adw serotype)
SBAS2:	SmithKline Beecham Adjuvant System 2
SMC:	Safety Monitoring Committee
SOP:	Standard Operating Procedure
TRAP:	Thrombospondin adhesion protein
UMB:	University of Maryland Baltimore
USAID:	U.S. Agency for International Development
USAMMDA:	U.S. Army Medical Materiel Development Activity
WB:	Western Blot
WHO:	World Health Organization
WRAIR:	Walter Reed Army Institute of Research

2 GLOSSARY OF TERMS

Participant(s): Term used throughout the protocol to denote the enrolled individual(s).

Subject(s): Term equivalent to participant in the protocol.

Medical Monitor: An individual medically qualified to assure the responsibilities of the sponsor especially as regards the ethics, clinical safety of a study and the assessment of adverse events.

Study Monitor: An individual who is responsible for assuring proper conduct of a clinical study at one or more investigational sites.

Eligible: Qualified for enrolment into the study based upon strict adherence to inclusion/exclusion criteria.

Evaluable: Meeting all eligibility criteria, complying with the procedures defined in the protocol, and, therefore, included in analysis (see Section 12.3 for details on criteria for evaluability).

Reactogenicity: refers to the both the expected and unexpected symptoms and signs that are associated with the administration of a vaccine. These include local reactions such as erythema, induration, and tenderness, as well as systemic reactions such as fever, malaise, myalgias, and arthralgias.

Solicited symptoms: Symptoms that are identified by direct questioning/observation about specific symptoms.

Unsolicited symptoms: Symptoms that are mentioned spontaneously by participants or in response to general, open-ended questions such as “How have you been feeling?”

3 SUMMARY

**Title**	Double blind randomized controlled Phase I trial to evaluate the safety and immunogenicity of WRAIR's MSP1 candidate malaria vaccine (FMP1) adjuvanted in GlaxoSmithKline Biologicals' AS02A vs. Rabies vaccine in semi-immune adults in Bandiagara, Mali
**Indication/ Study Population**	MALARIA-EXPERIENCED ADULTS AGED 18-55 YEARS
**Principal Investigator**	MAHAMADOU A THERA, M.D., M.P.H.
**Rationale**	In clinical studies in malaria-naïve adults the FMP1/AS02A was safe and highly immunogenic. A study to evaluate its safety and immunogenicity in malaria-experienced individuals is currently being conducted in Kenya, an area where malaria transmission is intense and perennial. This will be the second study in an endemic area and will evaluate the safety and reactogenicity of the FMP1/AS02A in a population exposed to more limited and seasonal malaria transmission pressure.
**Objectives**	**Primary**<br>To evaluate the safety and reactogenicity of WRAIR's MSP1 malaria vaccine (FMP1) adjuvanted in GlaxoSmithKline Biologicals' AS02A in malaria-experienced Malian adults.<br>**Secondary**<br>To evaluate the humoral immune response of WRAIR's MSP1 malaria vaccine (FMP1) adjuvanted in GlaxoSmithKline Biologicals' AS02A in malaria-experienced Malian adults.
**Study design**	• Double blind, randomized, controlled (Imovax® Rabies vaccine)<br>• Phase I<br>• One study center<br>• Study duration: approximately 12 months per subject<br>• Immunization schedule: Study days 0, 30 and 60<br>• Route: IM in deltoid muscle
**Number of subjects**	40 subjects (two groups of 20 each)
**Co-primary endpoints**	• Occurrence of solicited symptoms after each vaccination during an 8-day follow-up period (day of vaccination and days 1, 2, 3 and 7 after vaccination).<br>• Occurrence of unsolicited symptoms after each vaccination during a 31-day follow-up period (day of vaccination and 30 subsequent days).<br>• Occurrence of serious adverse events throughout the study period.
**Secondary endpoint**	• Titers of MSP1 antibody at each time point where serology samples are analyzed.

4 INTRODUCTION AND BACKGROUND

4.1 Malaria parasite life cycle

Among the four species of Plasmodium that cause human malaria, *P. falciparum* is responsible for most disease and death from malaria. Its life cycle is complex. Disease occurs as a result of the asexual blood stage when parasites invade and grow inside red blood cells. *P. falciparum* virulence is partially explained by its ability to use various receptor pathways to invade red blood cells of all ages. Red blood cells infected with *P. falciparum* bind to endothelium or placenta allowing the parasite to avoid spleen-dependent killing mechanisms but contributing much to pathogenesis[1].

Anopheline mosquitoes inject sporozoites into the subcutaneous tissue and less frequently directly into the blood stream. The sporozoites then travel to the liver, where they invade hepatocytes. About 6-10 days later, each infected hepatocyte releases 20,000-40,000 merozoites into the bloodstream. Despite the destruction of liver cells, no disease results from the infection during parasite development within hepatocytes. Only the blood stages of the infection produce clinical symptoms and malaria disease.

Red blood cells are invaded through a determined sequence. *P. falciparum* must engage receptors on red cells for binding[2] and undergo apical reorientation, junction formation, and signaling[3,4]. The parasite then induces a vacuole derived from the red cell plasma membrane and enters the vacuole by a moving junction. Within this parasitophorous vacuole *P. falciparum* develops over 48 hours producing around 17-32 merozoites, each able to invade other red cells. Particular to *P. falciparum* is its ability to modify the surface of the red blood cell in a way that the infected cells can adhere to the vascular endothelium and other tissues, where they may cause disease. Parasite sequestration in various organs (brain, heart, liver, kidney, placenta) contributes to the pathogenesis of malarial disease.

Following a number of intra-erythrocytic cycles, a small proportion of asexual parasites convert to gametocytes that are critical for the transmission of the infection to others through female Anopheline mosquitoes. Gametocytes cause no disease and there is no known induced natural immune response to this intracellular sexual form of the parasite.

4.2 Disease burden

Malaria is a major threat to the health of two billion people living mainly in sub-Saharan Africa, Tropical Asia, Latin America and Oceania. Less developed areas in the world, specifically in Africa, bear the heaviest burden of malaria. The most dangerous parasite species, *P. falciparum*, is responsible for more than one million deaths worldwide each year. More than 90% of these deaths occur among sub-Saharan African children under 5 years old. In area of stable malaria transmission 25% of all-cause mortality in 0-4 year old children has been directly attributed to malaria[5]. Evidence from impregnated bed net trials in West Africa indicates that malaria could account directly and indirectly for as much as 60% of all-cause mortality in children aged less than 5 years old[6-8]. A testament to its effect in school-age children, up to 50% of medically related school absences are attributable to malaria[9]. The overall impact of malaria on human capital development in African children remains unexplored, but it is probably substantial[10]. The impact of malaria on the productivity of adults living in endemic areas has implicated malaria as a major obstacle to development and a contributing cause of poverty. Furthermore, malaria constitutes an

increasing hurdle to foreign investment and trade. In areas of intense malaria transmission, the disease generates a complex set of biologic and behavioral responses with long-term effect on economic growth and development[10].

4.2.1 Malaria in Mali

In Mali, malaria is a leading cause of morbidity in the general population and of mortality in children aged less than 10 years. Malaria transmission is seasonal, and varies from very high levels of transmission in the wet holoendemic southern areas of Sikasso to malaria epidemic-prone areas at the fringe of the Sahara desert in the dry northern areas of Tombouctou, Gao and Kidal, where malaria transmission is limited to a short rainy season in August.

Between these extremes, in the region of Mopti in the central part of Mali, malaria transmission is high with marked seasonal variations. Transmission is by *A. gambiae s.l.* from July through November. In 1995, entomologic inoculation rates (EIR) peaked at 348 infective bites/person/month in September in the flooded rural areas of Mopti and were undetectable in April-May (MD Thesis, A. Dicko, University of Mali 1995). Epidemiologic surveys conducted during the rainy seasons of 1993 and 1994 found that 40-80% of children aged less than 10 years were carrying blood-stage parasites.

Bandiagara, (pop. 12,500) located 700 km northeast of Bamako in the heart of the Mopti region on the Dogon plateau, will be the site for this study. Although it is in the Mopti region, it is 75 km from the Mopti flood plain and its malaria epidemiology is quite different, with lower EIRs. Studies conducted from 1999 through 2001 have assessed year-to-year variations in the incidence of malaria disease, stratified by age. Children aged 0-5 years had a similar incidence of at least one clinical episode of uncomplicated malaria compared to older children aged 6-10 years: 86.2% (n=87) vs. 85.5% (n=69), respectively. No marked yearly variation in the incidence of malaria has been observed from 1999 through 2001. The average number of clinical episodes of malaria per child and per transmission season was 1.92, with a few children experiencing a maximum of four clinical episodes[11].

Adults living in highly malaria endemic regions such as most of subSaharan Africa are generally considered to be semi-immune; they have experienced several episodes of malaria and are susceptible to infection but protected against malaria disease. Our studies in this area since 1994 show that the annual incidence of malaria infection is well over 100%. Thus any adult resident of Bandiagara town can be considered to be "malaria experienced."

In this setting, 63% of the population are Dogon, an ethnic group in which the prevalence of the hemoglobin C (HbC) gene is 5 times higher than the prevalence of hemoglobin S with heterozygosity of 15-20%[12,13]. Case-control studies conducted in this population in 1997-98 showed that carriage of HbC among the Dogon was associated with a protective efficacy of 80% in the reduction of the risk of severe malaria[13].

Chloroquine is the first-line antimalarial therapy recommended by the Malian National Malaria Control Program and remains effective in Bandiagara[14]. Clinical efficacy rates of chloroquine were above 90% when repeatedly assessed by the WHO *in vivo* test protocol in 1997 through 2000[15]. For the treatment of uncomplicated malaria, the second-line drug sulfadoxine-pyrimethamine had an adequate clinical response rate of 95.6% (n=253) in 1999.

Census data designed to determine the population at risk showed the incidence of severe malaria among children aged six years or less was 2.5% (n=2284) in 2000. Hyperparasitemia (more than 500,000 asexual parasites/µl) and cerebral malaria were the most common forms of severe malaria, present in 59% and 40% of severe cases, respectively (with multiple diagnoses possible). Severe anemia, defined as hemoglobin < 5g/dl, was

present in 18.6% of severe malaria cases (unpublished data). The features of severe malaria in Bandiagara are therefore quite different from what was observed in western Kenya (the site of the ongoing Phase I trial of FPM-1/AS02A), where severe anemia represented 70% of severe malaria cases[16].

4.3 Rationale for a malaria vaccine:

A safe and effective malaria vaccine in the context of the spread of resistance to antimalarial drugs would be a major contribution to existing control tools. The objective of developing an effective malaria vaccine has been a focus of malaria research for many years. However, developing an effective malaria vaccine has been an elusive goal, and the few malaria vaccine candidates that have progressed to clinical trials have so far shown limited or no efficacy. Two critical steps of the malaria life cycle have been closely examined as potential targets for vaccine-induced immunity, the invasion of liver cells by sporozoites and the invasion of red blood cells by merozoites.

The sporozoite stage of the malaria parasite first attracted the attention of researchers as the most logical target for vaccine-induced immunity. If invasion of the liver by the relatively few sporozoites that are injected by a mosquito could be prevented, the host would have sterile immunity. The identification and cloning of the circumsporozoite protein (CS), which coats the surface of the sporozoite, was the first major step towards this objective[17]. This protein is critical for the invasion of liver cells and contains a peptide sequence that binds to the surface of liver cells[18]. The CS gene of *P. falciparum* is composed of a central repeating sequence (NANP) flanked by two non-repeat sequences. The first experimental malaria vaccines tested were based on this repeat sequence; they were poorly immunogenic and only protected a small proportion of the participants immunized[19,20]. Although the efficacy was poor, these studies served as proof-of-concept that sterile immunity against malaria can be induced by immunization with a synthetic subunit vaccine.

Subsequent efforts concentrated on the development of formulations with enhanced immunogenicity. Modifications such as the addition of *Pseudomonas aeruginosa* toxin A[21], encapsulation in liposomes containing monophosphoryl lipid A (MPL)[22,23], and inclusion of a mixture of MPL, mycobacterial cell wall skeleton and squalene[24] and the fusion to hepatitis B[25,26] generally resulted in higher antibody levels but, contrary to expectations, did not significantly improve the efficacy. Most recently, GSK and the WRAIR developed a recombinant molecule that contains important T helper epitopes from CS as well as the $(NANP)_{19}$ repeat fused to hepatitis B[25]. In the initial trials when this molecule was adjuvanted with SBAS2, containing MPL, the saponin QS21[27] and a proprietary oil-in-water emulsion, 6 of 7 participants were protected[28]. However, the protection was of short duration[29]. Subsequent studies have found the estimated efficacy of the RTS,S/SBAS2 vaccine to range from 40 to 50%[30]. One field trial in The Gambia has also corroborated these results[31].

The merozoite stage of the parasite, like the sporozoite, is a logical target for a malaria vaccine since blockade of erythrocyte invasion will prevent clinical disease. Therefore, the identification of the molecular mechanisms of merozoite invasion of red blood cells has been an active area of investigation in the field of malaria vaccine research. Several antigens have been identified that are involved in merozoite invasion of red cells. One of the most studied of these antigens and a promising blood stage vaccine candidate is the merozoite surface protein 1 (MSP1). MSP1, a 195 kDa antigen found on the surface of merozoites undergoes processing by proteolytic cleavage to a 42 kDa fragment and further to a 19 kDa fragment that has been implicated in the invasion of erythrocytes by the merozoite[32]. Several lines of evidence lead to the conclusion that MSP1 is a promising vaccine candidate.

Antibodies directed against portions of MSP1, in particular against the 19 kDa C-terminal fragment, inhibit erythrocyte invasion[32,33]. At least one field study has demonstrated an association between the existence of antibodies against MSP1 and resistance to clinical malaria[34]. Immunization with recombinant fragments of this molecule also protects monkeys against *P. falciparum*[35] when used with Complete Freunds Adjuvant, and mice against *P. yoelii*[36,37]. Passive transfer of immune sera in mice also confers protection[38]. Although the weight of the evidence indicates that antibodies against the C-terminal fragment of MSP1 are protective, in one case immunization with C-terminal constructs did not result in protection[39]. Lack of protection in some cases could be due to the use of antigenic constructs that do not have a proper conformational structure since recognition by the immune system of this region of MSP1 is known to be dependent upon conformation. MSP1 is also the target of CD4+ T cells and several T-cell epitopes have been identified[40,41].

A recombinant version of the 42 kDa C-terminal portion of MSP1 has been produced at the Walter Reed Army Institute of Research, FMP1 (Falciparum merozoite protein 1), as a histidine-tagged (His6) fusion protein in *E. coli*. The antigen is derived from the 3D7 clone of *P. falciparum* and contains both T-cell and B-cell epitopes. Monoclonal antibodies raised against native parasite MSP1 recognize correctly folded conformational disulfide-bonded epitopes within the recombinant 42 kDa antigen. Additionally, the structural fidelity of this preparation has been confirmed by demonstrating that the recombinant 42 kDa antigen binds specifically to human erythrocytes in a manner analogous to native parasite-derived MSP1 binding to red blood cells. An efficient fermentation and purification process has been developed for the production of this antigen on a scale compatible with industrial manufacture. The vaccine is formulated in the same adjuvant system used in the RTS,S vaccine, now called AS02A. In order to increase product stability, the $MSP1_{42}$ antigen is manufactured as a lyophilized product and reconstituted just prior to injection.

The study proposed here is a phase I safety and immunogenicity study of FMP1 in Mali, in an area of lower malaria transmission intensity than western Kenya, where FMP1 is currently being tested for safety and immunogenicity. If shown to be safe and immunogenic, further studies will be planned with this formulation to assess its efficacy, either alone or in combination with other malaria vaccine candidates, possibly including RTS,S, other genotypes of MSP1 or other blood stage antigens.

4.3.1 Clinical Experience with the AS02A Adjuvant

The adjuvant system AS02A, previously known as SBAS2, consists of an oil-in-water emulsion combined with two immuno-stimulants, Monophosphoryl Lipid A (MPL) and a saponin derivative known as QS21. QS21 is a highly purified component of a saponin agent derived from the soap bark tree, *Quillaja saponaria*[26,42,43]. MPL is a detoxified, deacylated form of monophosphoryl lipid A, derived from the lipopolysaccharide (LPS) of *Salmonella minnesota*. LPS, and more specifically, its lipid A component, has long been known for its strong adjuvant effects; however, until recently, its high toxicity precluded its use in a vaccine formulation. Ribi et al.[22] showed that the monophosphorylated form of lipid A retains its adjuvant function and almost completely loses its endotoxin effects. Subsequently, the 3-deacylated form of MPL was shown to have a further decrease in its toxicity as tested in small animals, but retains its immunopotentiating effect[44]. Several immunogenicity studies performed in mice, guinea pigs, monkeys, and humans have shown that inclusion of 3D-MPL into a vaccine preparation potentiates both specific antibody and cellular immune responses[44,44-46]. The term MPL in this protocol refers to the 3-deacylated form of the compound. To date, the bulk of the experience with this formulation in malaria vaccines has been in conjunction with the RTS,S antigen reviewed above.

AS02A is the same adjuvant mixture as AS02/SBAS2, both containing the two immunostimulants QS21 and 3D-MPL, in an oil-in-water emulsion, but without thimerosal as a preservative in AS02A. Clinical experience with AS02A in 406 malaria naïve and malaria experienced adults (totaling over 1100 vaccine doses) and 150 children, found the vaccine/adjuvant combination to be acceptably reactogenic.

The largest clinical experience thus far has been in Gambian adults, in a series of Phase 1 and 2b studies in which seven hundred and eleven doses of RTS,S/AS02 were administered. A pooled analysis of the reactogenicity showed that headache (34%) and malaise (25%) were the most frequently reported general symptoms. There were 4 reports of a general symptoms of maximum intensity (grade 3) probably or suspected to be related to vaccination in this population. All were reported in a phase 2b efficacy study after administration of a 4th dose booster vaccination: 1 case of arthralgia, 2 cases of headache and 1 case of malaise (0.1%, 0.3% and 0.1% respectively of documented doses). The majority of solicited local or general symptoms were of short duration (4days) and all these adverse events reported resolved without sequelae. There was no significant increase in reactogenicity after subsequent (up to 4) doses. In these studies 4 SAEs have been reported. Of these, only one SAE probably related or suspected of being related to vaccination (elevated ALT) occurred during the trial after dose 2. However in the same study, four cases of elevated ALT were reported as SAEs in the control group (Rabies). There were 3 other reports judged unlikely to be related to vaccination, these were: 1) erectile impotence, 2) hospitalization with jaundice and urinary tract infection 5 months after study completion (6 months after the last dose) died the next day of suspected fulminant hepatitis, 3) pneumonia cirrhosis and hepatocellular carcinoma reported 5 months after dose 3 and died 6 months later. A summary table of the clinical experience with AS02 and AS02A in adults and children is provided below.

Vaccine / Trial	Location	Number of Participants	Total Number of Vaccinations in Trial	Reactogenicity Results
FMP1/AS02, RTS,S/AS02 or FMP1+RTS,S/AS02	WRAIR	60 adults, malaria naïve	176	No vaccine related SAEs, 4 severe injection site reactions (pain), all resolved in 24 hours; no significant laboratory abnormalities
FMP1/AS02	WRAIR	15 adults, malaria naïve	45	No SAEs or severe reactions; no significant laboratory abnormalities related to vaccination
FMP1/AS02A	Kenya	40 adults, malaria experienced	117	No vaccine related SAEs, 17 severe local reactions, no severe systemic reactions
TRAP/AS02 or TRAP+RTS,S/AS02	WRAIR	65 adults, malaria naïve	> 100	No SAEs, no significant hematologic or biochemical laboratory abnormalities
RTS,S/AS02	The Gambia	150 children	> 150	No vaccine related SAEs or severe reactions
RTS,S/AS02	The Gambia	226 adults, malaria experienced	711	1 SAE possibly related to vaccination (elevated ALT – resolved without sequelae), headache and malaise most common AEs

In all, these studies, with a cumulative experience of over 500 non-immune and semi-immune participants, suggest that, thus far, the AS02A has a good safety profile.

4.3.2 The FMP1 Vaccine

The FMP1 study vaccine consists of lyophilized recombinant $MSP1_{42}$ produced in and purified from *E. coli* bacteria. This antigen consists of the 42 kDa carboxy-terminal end of MSP1 comprising 392 amino acids derived from the merozoite surface protein MSP1 of the malaria parasite, *P. falciparum.* The protein is expressed as a fusion protein to which six histidine residues are added to the N-terminus to facilitate purification. The total amount of antigen in a single dose is 50 μg. The dose was chosen on the basis of results from Phase I studies in malaria-naïve adult participants as described below. The lyophilized antigen will be dissolved in 0.5 ml of AS02A adjuvant prior to injection.

4.3.3 Pre-clinical Toxicity, Safety and Reactogenicity of the FMP1/AS02 Malaria Vaccine

The FMP1/AS02 formulation was safe, well tolerated, and highly immunogenic in a pre-clinical trial conducted in *Macaca mulatta* performed at the Armed Forces Research Institute of Medical Sciences in Bangkok, Thailand. Eight monkeys were immunized intramuscularly on a 0, 1, 3 month schedule using a standardized safety and immunogenicity model. All eight immunized monkeys seroconverted to the immunogen. Group mean antibody titers against a 19 kDa subunit of the immunogen rose to 10,000 ELISA units after 2 doses and to 17,000 units after 3 doses. Rhesus antibody was also highly positive versus *Plasmodium falciparum*-parasitized red blood cells in an indirect fluorescence antibody assay.

Clinical grade lots of $MSP1_{42}$ adjuvanted with AS02A have been administered to mice and guinea pigs. No significant local or systemic toxicities were observed in any of the animals. Immune responses to the formulation in mice indicated excellent antibody responses to the FMP1 antigen.

4.3.4 Clinical Experience with FMP1/AS02A

A Phase I open label, dose-escalation study to evaluate the safety, reactogenicity, and immunogenicity of FMP1 with AS02 adjuvant was conducted at the WRAIR. Fifteen participants were randomized to receive either 10 μg (N=5), 25 μg (N=5), or 50 μg (N=5) doses of vaccine on a 0, 1, 3 month schedule by IM injection. The ratio of adjuvant to antigen was constant (i.e. 0.10 ml, 0.25 ml, or 0.50 ml of AS02). After 3 doses, there were no grade III reactions, defined as a reaction that prevents normal day-to-day activities, or serious adverse events. The laboratory tests have been normal except for an occasional elevated CPK level detected both at the time of immunization and 48 hours after immunization probably related to physical activity of the participants. Seroconversion occurred in all 15 individuals after a single immunization. Boosting of antibody levels occurred after 2nd and 3rd doses. The antibody titers against $MSP1_{42}$ are summarized below in normalized OD units (the dilution that gives an OD_{415}=1).

Table 1: Immunogenicity of varying doses of FMP1/AS02 (OD units)

Vaccine Cohort

1/5 Dose (10 μg)	Day 0	Day 14	Day 28	Day 42	Day 84	Day 98
Average	12	312	462	18066	7371	32648
Std Dev	8	191	236	8406	6304	24046
Geo Mean	10	272	412	16440	5749	26626
**1/2 Dose (25 μg)**						
Average	28	1285	2530	44172	ND	57771
Std Dev	6	1882	2631	21176	ND	24192
Geo Mean	28	636	1762	40744	ND	53569
**Full Dose (50 μg)**						
Average	32	688	990	32461	15914	50053
Std Dev	32	414	306	19307	5650	29991
Geo Mean	22	586	951	28448	14938	42799

Although the above results suggest little or no difference between 25 and 50 μg, IFA titers against whole merozoites were higher in the 50 μg group than in the 25 μg group. Because antibody titers against whole merozoites may be more relevant, the dose of 50 μg has been chosen as the dose for the Phase I trial outlined in this protocol.

Based on the safety and immunogenicity results from the open-label FMP1/AS02 Phase I dose-escalation study, a double blind Phase I/IIa trial was conducted at the WRAIR to evaluate the potential synergy of FMP1/AS02 and RTS,S/AS02. Sixty participants were randomized into 4 groups of 15, and received vaccines on a 0, 1, and 3 month schedule. The first group received separate arm injections of FMP1/AS02 and AS02; the second group separate arm injections of RTS,S/AS02 and AS02; the third group separate arm injections of FMP1/AS02 and RTS,S/AS02, the fourth group an extemporaneous mix mixture of FMP1/RTS,S/AS02 in separate arms. A fifth group (N=12) served as infectivity controls. The study was designed to assess safety, immunogenicity, reactogenicity, and efficacy defined as either delay or prevention of parasitemia in comparison to controls as determined by light microscopy. The primary efficacy analysis was to determine major agonist or antagonist effects of FMP1 on RTS,S/AS02-mediated protection. Secondary analyses are planned to include experimental molecular analyses to determine delay in release of hepatic merozoites, and correlation of efficacy results with the functional and quantitative antigen-specific antibody. Sixty volunteers received their first immunizations in May 2001, 60 received their second immunizations in June, and 56 received their third immunization in August 2001. The immunizations were minimally reactogenic and well tolerated. The most common side effects were minor pain, redness or swelling at the injection site. Of the total 176 immunization procedures involving injections at 352 sites, there were four Grade 3 reactions which consisted of pain at rest and which in all cases resolved by 24 hours post injection. There were no clinically significant biochemical or hematological abnormalities after immunization. As of 26 January 2003, there have been two SAEs reported: 1) a hospitalization for depression; and 2) a hospitalization for observation after an episode of chest pain. There have been no vaccine-related serious adverse events. After the third immunization 47 volunteers elected to undergo experimental malaria challenge in September 2002. Of the 47 challenged volunteers, 34 volunteers received FMP1/AS02 alone or in

combination with RTS,S/AS02. None developed any complications during detailed follow up. There was no evidence in this stringent malaria challenge model that FMP1/AS02 interfered with the protective efficacy of RTS,S/AS02.

This study was then followed by initiation in Kenya in April of 2002 of a controlled trial of FMP1/AS02A (full dose; 50 micrograms of antigen and 0.5 ml of AS02A) versus rabies vaccine in 40 malaria-experienced male and female adults. Dr. Jose Stoute is the Principal Investigator for this ongoing trial, entitled "Phase 1 trial to evaluate the safety and immunogenicity of WRAIR's MSP-1 malaria vaccine (FMP1) adjuvanted in GlaxoSmthKlines Biologicals' AS02A in Western Kenya." Forty volunteers received their first immunizations in April 2002, forty received their second immunization in May 2002, and 37 received their third immunization in June 2002. Statistics Collaborative Inc. prepared a report titled "Interim Report Open Version" dated 5 December 2002, which summarized the current data in an unblinded manner. There were a total of 17 instances of at least one Grade 3 reaction in association with vaccine administration; 13 of 40 were reported with the first immunization, 5 of 40 were associated with the second immunization, and 5 of 37 were reported with the third immunization. There were no Grade 3 systemic reactions. Seven serious adverse events were reported between 20 May and 18 October; these consisted of hospitalizations for: malaria (2), malaria plus pneumonia (1), sepsis (2), gastroenteritis (2). Based upon a review of the unblinded safety and immunogenicity data, the Data Safety Management Board gave permission to the field team to proceed with plans for a subsequent pediatric trial of FMP1/AS02A scheduled for 2nd quarter of 2003. Since review of the safety data, there have been an additional 3 SAEs reported: ectopic pregnancy with rupture (1), pneumonia (1), and pregnancy (1). The study team remains blinded as to whether these SAEs occurred in FMP1/AS02A or rabies vaccine recipients. This study is ongoing, with a final report expected in late 2003.

4.3.5 Justification of 0, 1, 2 Month Schedule

Most studies with FMP1 so far have been conducted using a 0, 1, and 3 month immunization schedule. The overall testing program of FMP1 aims at carrying out studies in children in subsequent trials. With this goal in mind, the ongoing study in Kenya and the present study use a 0, 1, and 2 month immunization schedule anticipating that this schedule will be more amenable to incorporation into the Expanded Program of Immunizations (EPI) of the WHO.

4.3.6 Comparison Vaccine

4.3.6.1 Immunogenicity

Having a comparator vaccine is particularly useful in Phase I trials conducted in malaria-endemic areas, since background immunity and natural exposure to malaria may make it difficult to interpret immunogenicity data. This is particularly a concern in this trial in a setting with seasonal transmission when some doses the vaccine will be administered in the non-transmission season. Rising titers of antibody to MSP1 could be due to immunization or to natural exposure or both. The use of a control group will permit comparison of immune responses and will result in a clearer interpretation of serological results. While a placebo control group would accomplish this same end, using a vaccine that is beneficial to the subjects increases the benefit to risk ratio, which is always relatively low in a Phase I trial. Based on dosing schedules and potential benefit to participants, potential choices for a

comparator vaccine include hepatitis B and rabies vaccines. We have chosen to use rabies vaccine as the comparator in part to be able to compare results directly with the ongoing study in Kenya, and in part because the available evidence supports a benefit for participants who receive rabies vaccine.

Rabies prevalence in Mali is not known but available data from the Ministry of Health's Division of Epidemiology suggest that the rabies burden is high. An average of 1,500 dog bites were reported to public health officials in Bamako, the capital of Mali, from 1996 through 1999. The vast majority of the dogs are unvaccinated and in most cases the status of rabies infection is unknown. Only 124 heads of dogs were examined for evidence of rabies infection. Rabies infection was found in 34 (27%) heads; 7 cases were negative and there were no reported results for 74 cases (60%). These incomplete data allow us to estimate that approximately 30% of dogs that bite humans may be carrying and potentially transmitting rabies infection. In the Bandiagara district health center, one case of human rabies is reported per year This is likely a gross underestimate of the true incidence of rabies cases given the general population's reliance on traditional healers and the relatively low utilization of the district health center. Of note, three dog bites were reported among the 40 study subjects during the Kenya trial.

Available data suggest that the Hepatitis B burden, although not well documented, is likely to be high in Mali. In 1980 a serologic study of 172 adults living in urban areas, found 97.2% had serologic markers indicating exposure to the Hepatitis B virus[50]. In 2001, a carriage rate of 15.5% for Hepatitis B surface antigen was found in pregnant women in Bamako[51].

Thus, the limited available evidence indicates that both rabies and Hepatitis B vaccinations would benefit the Malian population. We chose the rabies vaccine over Hepatitis B vaccine as the comparator vaccine because of the scientific benefit of standardization with the FMP1 protocol being implemented in Kenya. Imovax® Rabies produced by Aventis Pasteur, SA will be used as the comparator in this study. See Section 9.1.2 for more details. When Imovax® Rabies is administered according to the recommended immunization schedule (days 0, 7, 21), nearly 100% of subjects attain a protective titer. In two studies carried out in the US in 101 subjects, protective antibody titers >0.5 IU/ml were obtained by day 28 in all subjects. In studies carried out in Thailand in 22 subjects, and in Croatia in 25 subjects, antibody titers of >0.5 IU/ml were obtained by day 14 (injections on days 0, 7, 21) in all subjects[52-55].

High antibody titers have also been demonstrated with off-label immunization with Imovax® Rabies. Among participants in England, Germany, France and Belgium who received two doses one month apart, nearly 100% of the participants developed specific antibody and the geometric mean titer for the group was 10 IU[56-59]. The proposed immunization schedule of 0, 1, and 2 months is therefore expected to be highly successful in conferring protective immunity against rabies among the control participants.

4.3.7 Safety of Imovax® Rabies vaccine

Local and/or mild systemic reactions may occur after injection of Imovax® Rabies but these are usually transient and do not contraindicate continuing immunization. Imovax® rabies is a human diploid cell vaccine (HDCV). In a study using 5 doses of HDCV, local reactions such as pain, erythema, and swelling or itching at the injection site were reported in about 25% of recipients[60]. Mild systemic reactions such as headache, nausea, abdominal pain, muscle aches, and dizziness were reported in about 20% of recipients[60]. Two cases of neurologic illness resembling Guillain-Barré syndrome, a transient neuroparalytic illness, and a focal subacute central nervous system disorder temporally associated with HDCV has been

reported[61-63]. Systemic allergic reactions characterized by generalized urticaria and in some cases by arthralgia, angioedema, fever, nausea and vomiting have been reported following administration of HDCV. These reactions are uncommon in primary administrations but have been reported in up to 7% of persons receiving a booster dose[64].

4.3.8 Rationale for double blind controlled design

A double-blind controlled trial will allow assessment of vaccine safety in both groups in the conduct of the study. The sample size of the groups, however, will not allow detection of anything other than very large differences in the occurrence of adverse events between the two groups. The advantage of double blinding is to remove the potential for investigator and participant prejudgment about the effects of the two vaccines in the reporting of adverse events.

4.3.9 Justification for conducting FMP1 trial in Mali

A safe and effective vaccine that prevents malaria caused by *P. falciparum* would be an important addition to current methods used for controlling this serious infectious disease. Increasing drug resistance makes an effective vaccine to prevent infection and/or symptomatic disease an invaluable tool for malaria control. The safety and efficacy of malaria vaccines may vary according to the intensity of malaria transmission, which determines not only frequency and intensity of exposure to "natural boosting" but is also related to levels of naturally acquired immunity to malaria. It is therefore prudent to assess the safety and immunogenicity of FMP1 across the broad range of malaria transmission patterns that exist throughout Africa. Western Kenya and Bandiagara, Mali, represent two sites with very different transmission patterns, each of which is representative of many other malaria-endemic areas where a vaccine would eventually be offered.

5 OBJECTIVES

5.1 Primary objective

To evaluate the safety and reactogenicity of WRAIR's MSP1 malaria vaccine (FMP1) adjuvanted in GlaxoSmithKline Biologicals' AS02A in malaria-experienced Malian adults aged 18-55 years inclusive.

5.2 Secondary objective

To evaluate the humoral immune response of WRAIR's MSP1 malaria vaccine (FMP1) adjuvanted in GlaxoSmithKline Biologicals' AS02A in malaria-experienced Malian adults.

6 STUDY DESIGN AND METHODOLOGY

6.1 Overview

- Double blind, randomized controlled phase I study
- One study center
- Screening will be done within 35 days prior to the first immunization
- 40 adults will be randomized in a 1:1 ratio to receive either FMP1/AS02A or Imovax® Rabies vaccine
- Immunization schedule will be on study days 0, 30 +/- 7, and 60 +/- 7
- Route of immunization will be deltoid muscle IM
- Data will be collected onto source records and transcribed onto CRFs
- Study duration will be approximately 12 months per participant
- 8-day follow-up (day of vaccination and days 1, 2, 3 and 7 after vaccination) of solicited adverse events
- 31-day follow-up (day of vaccination and 30 subsequent days) of unsolicited adverse events
- Follow-up of serious adverse events (SAEs) until resolution
- Beginning Study Day 120, participants will be visited and assessed by local guides at home at monthly intervals and will be asked to return to clinic every 3 months for safety follow-up.

6.2 Number of participants/Center

Recruitment will be progressive until 40 adults of either gender who fulfill the inclusion criteria are included in the study. Volunteers will be recruited by non-coercive methods among adults 18-55 years of age residing in Bandiagara. Only adult volunteers will be included in this study. They will be recruited after coming voluntarily to the BMP clinic. No undue influence will be exerted upon volunteers to obtain their consent. In fact, after explaining the study to the potential volunteers, they will be allowed to leave and return later with their decision; this will allow time for them to discuss the study with their family and carefully consider their involvement in the study. Finally, at the BMP clinic, the individual consent process will be conducted in a separate and private room to ensure confidentiality, to reduce the likelihood of other participants influencing the decision to participate, and to allow further time to make a final decision.

The study will be conducted in Bandiagara. It has been the site of MRTC malaria epidemiological and entomological studies since 1993, and since 1998 has been the site of an NIH-supported contract for developing a site for testing malaria vaccines, known as the Bandiagara Malaria Project (BMP). The BMP has completed a full census of Bandiagara, established a laboratory with the capability of preserving sera, peripheral blood mononuclear cells, live parasites and DNA; and a clinical research center where malaria diagnosis and hemoglobin and blood glucose levels are routinely determined and where children with severe malaria are hospitalized and cared for. Since 1999, the BMP has conducted a case control study that has enrolled approximately 220 cases of severe malaria matched to 220 each of uncomplicated malaria and healthy controls. An ongoing cohort study of 400-440 subjects aged 3 months to 20 years with nested drug resistance studies has also been conducted continuously since 1999. The rate of loss to follow-up has consistently been less

than 7%. The BMP facilities are located within the Bandiagara District health center, where a two large recently renovated blocks of rooms including an air-conditioned clinical laboratory and vaccine storage preparation room and several private consultation rooms, are dedicated to BMP activities; and the Bandiagara Center for Research on Traditional Medicine where 2 rooms are exclusively used for BMP laboratory activities. Locked cabinets with limited access are used for data storage.

The BMP team has been trained to conduct GCP-compliant studies, using source documents, written informed consent and standardized case report forms. The BMP team has also established a strong trust and rapport with the community, and the community is very accepting of the possibility of conducting malaria vaccine trials there.

The extensive contact with the population has led to the development of mutual trust and the establishment of an ongoing informed consent process attempting to address issues related to interventional studies in resource-limited settings. Many discussions with local community leaders, heads of families and citizens through group meetings, and more limited group interviews have reviewed the need to obtain a written informed consent from study participants. The community has now become familiar with the informed consent process, including written, signed consent forms.

The informed consent process goes through the following steps:

i. A minimum 2 week screening and consent period.
ii. Explanation and clarification to local officials and community leaders, including the chiefs of quartiers (well-defined sections of the town), traditional healers, local medical staff, and school officials.
iii. Broadcast general information about the study through local radio station
iv. Allow time for leaders to communicate with community members and relay any additional questions or concerns.
v. Take time to explain protocols to heads of families.
vi. Careful word-for-word review of screening and study consent forms translated orally into local languages and dialects by research team.
vii. Upon recruitment, explanation of protocol to participants.

Consider informed consent as a dynamic procedure with re-consenting of study participants when new data becomes available that could affect participant safety and/or willingness to continue in the study.

The site is being connected to the MRTC central laboratory in Bamako via a VSAT system, which will allow a high-speed communication link with Bamako, U.S. partner institutions and the Internet.

6.3 Roles and responsibilities of key study personnel

a) Prof. Ogobara Doumbo, MD: Senior co-investigator; member of the protocol development team; responsible for obtaining approval from the local community and Malian authorities.

b) Mahamadou A Thera, MD: study PI ; member of protocol development team; responsible for the overall conduct of the study; responsible for obtaining study approval from the Malian IRB; responsible for obtaining individual informed consent; responsible for developing and maintaining updated clinical SOPs and source documents; clinical evaluation of study participants; management of AEs and SAEs. As the study PI, Dr. Thera will be intimately involved with all day-to-day study activities.

c) Christopher V Plowe, MD: Study co-PI; lead role in protocol development; coordination of communication among study partners and sponsors; assist with on-site supervision of all key study activities; clinical evaluation of study participants; management of AEs and SAEs.

d) Prof Dapa Diallo, MD: Senior co-investigator with overall responsibility for the BMP clinical laboratory; member of the protocol development team.

e) Alassane Dicko, MD, and Issaka Sagara, MD: co-investigators responsible for database design, data quality, data encoding, and the production of interim reports to the SMC, DSMB and sponsors.

f) Drissa Coulibaly, MD: co-investigator and Clinical trials Coordinator; responsible for ensuring that data collected onto source documents and CRFs are complete and accurate; will help in developing clinical SOPs and source documents. Dr. Coulibaly will work closely with Ms. Linda Rosendorf, CVD Regulatory Affairs Specialist.

g) Abdoulaye Kone, MD, Karim Traoré, MD and Ando Guindo, MD: clinicians responsible for the clinical evaluation of study participants, and the management of AEs and SAEs.

h) Moussa Sogoba, MD, Mohamed B Niambélé, MD, and : role in developing clinical SOPs and source documents; will participate in the internal monitoring of the study.

i) David Diemert, MD: co-investigator; member of the protocol development team; involved in the installation and management of the BMP clinical laboratory; clinician involved in the clinical evaluation of study participants and management of AEs and SAEs.

j) Linda Rosendorf, MS: CVD regulatory affairs specialist; responsible for pre-trial training of Clinical Trials Coordinator and Data Quality Manager; organization of study files in Maryland and in Mali; assist with coordination of activities during initiation of study.

j) Elissa Malkin, DO: clinician who may assist in the clinical evaluation of study participants and management of AEs and SAEs.

k) Louis Miller, MD: senior NIAID malariologist who has advised the protocol development team but will not play an active role in the conduct of the study.

l) Mounirou Baby, PharmD: key role in the installation and daily management of the BMP clinical laboratory.

m) Amagana Dolo, PharmD and Modibo Daou, PharmD: study pharmacists responsible for the integrity, accountability and maintenance of the cold chain for the study vaccines and adjuvant.

n) Kirsten Lyke, MD, Aric Gregson MD, Karen Kotloff, MD: experienced co-investigators from CVD Maryland who have contributed to protocol development and will be on-site to assist with the conduct of the study.

o) Janet Wittes PhD: main statistician for the study responsible for designing the CRFs and database and will play a lead role in the statistical analysis of the study endpoints.

p) Prof Hamar A Traoré, MD, Issa Benzacour, MD: Local Medical Monitors for the trial.

q) Other scientists listed as co-investigators on the protocol have actively participated in protocol development and will not be involved in conducting the study.

6.4 Inclusion criteria

- A male or non-pregnant female aged 18-55 years inclusive at the time of screening.
- For women, willingness not to become pregnant until 1 month after the last dose of vaccine
- Written informed screening and study consent obtained from the participant before study start.
- Available and willing to participate in follow-up for the duration of study (12 months)

6.5 Exclusion criteria

The following criteria will be checked at the time of study entry (i.e. following screening, at the time that participants are enrolled into the vaccine trial itself). If any apply at the time of study entry, the subject must not be included in the study:

- Previous vaccination with an investigational malaria vaccine or with any rabies vaccine.
- Use of any investigational or non-registered drug or vaccine other than the study vaccine(s) within 30 days preceding the first dose of study vaccine, or planned use up to 30 days after the third dose.
- Chronic administration (defined as more than 14 days) of immuno-suppressants or other immune-modifying drugs within six months prior to the first vaccine dose. This will include oral steroids and inhaled steroids, but not topical steroids.
- Planned administration/administration of a vaccine not foreseen by the study protocol within 30 days before the first dose of study vaccine(s) with the exception of tetanus toxoid.
- Previous vaccination with a vaccine containing MPL and/or QS-21 such as RTS,S.
- Any confirmed or suspected immunosuppressive or immunodeficient condition, including human immunodeficiency virus (HIV) infection.
- Any confirmed or suspected autoimmune disease
- History of allergic reactions or anaphylaxis to immunizations or to any vaccine component.
- History of serious allergic reactions to any substance, requiring hospitalization or emergent medical care
- History of allergy to tetracycline, doxycycline or neomycin
- History of splenectomy
- Serum ALT $\geq$35 IU/L
- Serum creatinine level >133 µmol /L (1.5 mg/dL)
- Hb <11 g/dL for males and <10 g/dL for females
- WBC $<3.0 \times 10^3/mm^3$ or $>13.5 \times 10^3/mm^3$
- Absolute lymphocyte count $\leq 1.0 \times 10^3$ /µl
- Thrombocytopenia < 100,000/µl
- More than trace protein, more than trace hemoglobin or positive glucose in urine

- Administration of immunoglobulins and/or any blood products within the three months preceding the first dose of study vaccine or planned administration during the study period.
- Suspected or known current alcohol or illicit drug abuse.
- Pregnancy or positive urine beta-HCG on the day of or prior to immunization.
- Breastfeeding
- Simultaneous participation in any other interventional clinical trial.
- Acute or chronic pulmonary, cardiovascular, hepatic, renal or neurologic condition, or any other findings that in the opinion of the PI may increase the risk to the participant from participating in the study.
- Other condition that in the opinion of the investigator would jeopardize the safety or rights of a participant in the trial or would render the participant unable to comply with the protocol

6.5.1 Justification for the Exclusion of Children

The FMP1/ASO2 vaccine has been tested in 60 adults in the United States, and 40 adults in Kenya. In these combined studies, no serious safety concerns were identified. However, this will be the first time that this vaccine formulation will be tested in an area of seasonal malaria transmission; additionally, the intensity of malaria transmission is much lower in Mali than it is in western Kenya. Differences in transmission dynamics may affect both the safety and immunogenicity of the vaccine, therefore it is felt that it is more ethical to perform this Phase I study first in adults who can give full, informed independent consent. Following this study, assuming the vaccine is shown to be safe and immunogenic in this study area, further studies in children are anticipated.

6.5.2 Rationale for using clinical assessment of immunosuppression

We do not plan to test for HIV at the time of screening for two reasons. First, HIV seroprevalence is 1.7% in Mali, one of the lowest rates in sub-Saharan Africa. Although no serosurveys have been done in Bandiagara itself, this site is in a remote rural area and almost certainly has a lower prevalence rate than the average for the entire country. After working at this site for the past five years, we have only very rarely encountered persons with illnesses that raised clinical suspicion of an immunosuppressive disease. Therefore, the training of staff and establishment of programs that would be necessary for voluntary counseling and testing for HIV would likely yield few, if any, cases of HIV in this small study.

Second, this study has been designed to produce comparable results to the WRAIR study now in the final stages of follow-up in Kenya. In that study, a similar approach was taken to exclude persons with clinical evidence of immunosuppressive disease but not test for asymptomatic HIV infection, based on the rationale that it is necessary to assess the safety and immunogenicity of this vaccine in generally healthy adults who are representative of the population from which they are drawn. In Kenya, where rates of HIV infection are higher, this general population includes many persons living with HIV and a study that excluded them would be of less value. Eventually, it will be necessary to demonstrate the safety and immunogenicity in persons living with HIV for any malaria vaccine to be employed in Africa, but because of the low rates of HIV infection in Mali these studies will have to be conducted elsewhere.

6.6 Treatments that could potentially interfere with the vaccine-induced immunity

The following criteria will be checked at each visit. If any become applicable during the study, the subject will not be required to discontinue the study, but a separate analysis may be done that excludes these individuals. See section 12.3 for definition of study cohorts/datasets to be evaluated.

- Use of any investigational drug or vaccine other than the study vaccine(s) during the study period.
- Chronic administration (defined as more than 14 days) of immunosuppressants or other immune-modifying drugs during the study period and chronic daily use of inhaled steroids. Intermittent use of inhaled and topical steroids are allowed.
- Administration of a vaccine not foreseen by the study protocol during the period starting from 30 days before the first dose of vaccine(s) and ending 30 days after the third dose.
- Administration of immunoglobulins and/or any blood products up to 30 days after the last dose of vaccine.
-

6.7 Contraindications to vaccination

The following criteria will be checked prior to each immunization and are contraindications to further immunization. However, the study participants will be encouraged to remain in the safety evaluation for doses received.

- Systemic hypersensitivity reaction following administration of the study vaccine. Severe (i.e., Grade III) local reactions will be evaluated by the Local Medical Monitor and Safety Monitoring Committee as outlined in Sections 14.3 and 14.4 to determine whether or not further doses of vaccine should be administered.
- Positive urine β-HCG

6.8 Indications for deferral of immunization

The following adverse events constitute grounds for deferral of vaccine administration at that point in time; if any one of these adverse events occurs at the time scheduled for vaccination, the subject may be vaccinated at a later date, within the time interval specified in the protocol section 7.1.6, or withdrawn at the discretion of the investigators. The subject must be followed until resolution of the event, as with any adverse event (see section 10.4). A subject who is withdrawn from the study, will be encouraged to remain in the safety evaluation for the duration of the study.

- Oral temperature $>37.5^{o}C$ or evidence of clinical malaria (see section 7.2.1) at the time of vaccination will warrant deferral of immunization until fever and symptoms resolve
- Any other condition that in the opinion of the investigator poses a threat to the individual if immunized or that may complicate interpretation of the safety of the vaccine following immunization.

Such individuals will be followed daily in the clinic until the symptoms resolve or the window for immunization expires. No further vaccination will be performed if the subject does not recover (oral temperature $\leq 37.5^{o}C$ and/or lack of symptoms) within 7 days of the originally scheduled vaccination date. The subject, however, will be followed for safety and immunogenicity.

If the individual meets any of the above criteria for deferral on the day of first immunization the PI may elect to exclude the subject from further participation in the study.

7 CONDUCT OF THE STUDY

7.1 General study aspects

7.1.1 Screening and inclusion process

Screening and recruitment will be progressive until the desired number of study participants is included. Non-coercive means of recruitment will be used according to ICH/GCP requirements and following MRTC policy. After community information is disseminated as described in Section 6.2, all interested and potentially eligible adults aged 18-55 will be invited to visit the study clinic on a specific date. These individuals will receive oral and written explanation of the study, after which screening consent will be obtained from those willing to participate. All screening tests, medical history and examinations will be performed only after screening consent is obtained. Any clinically relevant finding that is discovered upon screening will be treated appropriately. Detailed SOP's of all procedures will be on file with the investigators.

Upon screening, the Investigator will prepare a case report form (CRF) for each participant. A unique identification number will be assigned to each study participant (PID). The CRF will be labeled with the participant's identification number; it will contain information on the participant's date of birth and medical history, date of screening visit, whether the participant was included or not and, where applicable, the reasons for exclusion from the study.

Participants will provide a medical history, with special attention to any history of recurrent infections to suggest immune suppression, previous history of splenectomy and prior vaccine reactions. They will also undergo physical examination and laboratory screening tests, which include (see section 8.3 for details on laboratory testing to be performed): complete blood count (CBC) creatinine, ALT , urine analysis, and urine β-HCG (for females to exclude pregnancy). Urine β-HCG will be obtained on female participants just before each immunization. A participant who meets any of the exclusion criteria will be excluded. Participants excluded from this study because of significant abnormalities will be managed initially by study clinicians and referred to local health center for evaluation as necessary. All screening tests must be completed within the 35 days prior to entry into the study. Laboratory studies may be conducted at other times during the course of the trial if the investigators judge it necessary for the safety of the participant. All screening and follow-up diagnostic laboratory testing will be performed at the Bandiagara Malaria Project laboratory in Bandiagara and if applicable at the MRTC clinical laboratory in Bamako. Information gathered during screening (medical history, physical examination and laboratory analysis) will be recorded in the source documents and then transcribed into the CRFs.

The investigators will select 40 eligible participants who fulfill the inclusion criteria and none of the exclusion criteria, and invite them to sign the study consent. Each study subject will receive a photo ID card and a copy of the photo ID will be attached to the source document folder for each participant.

7.1.2 Vaccination process

Before each vaccination, criteria for continued eligibility will be reviewed and verified. Once the volunteer is deemed eligible to receive vaccination, the volunteer will be randomized and will receive a randomization number (ERN). The first volunteer to be vaccinated will be assigned ERN-01, the second volunteer will be assigned ERN-02, and so on, in the order of presentation. A history-directed physical examination will be done and oral temperature, blood pressure, pulse and baseline general symptoms will be recorded. Venous blood will be collected for laboratory analysis as detailed in sections 8.1 and 8.2.

After the participant's identity is checked by comparing his/her photo ID and PID with that on the CRF, he/she will be vaccinated by intramuscular injection into the left deltoid muscle. If any local impairment prevents administration of the vaccine into the upper arm for that particular dose, the vaccine may be administered into the opposite arm in the deltoid region (see section 9.2). Vaccination will be done on study days 0, 30 +/- 7 and 60 +/- 7.

7.1.3 Procedures to be followed for post-immunization evaluations

After immunization, the participant will be observed in a separate room for assessment of local and systemic reactions. The participant will be observed for a minimum of 30 minutes post-vaccination. Signs and symptoms (as detailed in Section 10) will be solicited and recorded in the source document by the investigators according to adverse events recording procedures (see Section 10).

Participants will complete an 8-day follow-up after each vaccination at the BMP clinic center (including day 0, the day of vaccination). All adverse events will be followed until resolution. If any symptom persists beyond the 8-day follow-up period the participant will be followed daily until resolution of the adverse event.

After the 8-day follow-up period, participants will be asked to come to the BMP clinic center on days 14 +/- 3 and 30 +/- 7 after vaccination. At each visit, the participants will be evaluated by a study physician. A complete clinical examination will be performed and information on any solicited or unsolicited symptoms since the last visit will be collected. Every effort will be made to ensure compliance with visits. Local guides will conduct home visits to participants a day before their scheduled visit at the clinic. If a participant does not appear for a scheduled clinic visit, the local guide will visit him/her again and drive the participant to the clinic center. If a serious adverse event (SAE) has occurred, appropriate measures will be taken to notify the Principal Investigator, Local Medical Monitor, SMC, and all sponsors and IRBs as described in section 10.5.2.

7.1.4 Competencies of staff

The BMP clinic team is composed of medical doctors and doctors of pharmacy who are experienced in conducting complex studies with long periods of follow-up and complying with ICH/GCP requirements. The past studies have been implemented with the aim of preparing the team to conduct GCP-compliant malaria vaccine trials. The team's training program in 2001 included GCP workshops in Mali, in Baltimore at the University of Maryland's Center for Vaccine Development, and at the John Hopkins Summer Institute on Biostatistics and Epidemiology. Several of the study investigators have attended the Harvard course on bioethics directed by Dr. Richard Cash. Before this trial commences the study team will attend a GCP workshop, and specific training on this protocol and its study procedures will be conducted. A specific training program on protocol study procedures will be designed for the local guides and supporting staff in Bandiagara. The PI, co-investigators and the BMP clinic team will carry out all the study procedures. The local guides will have the

responsibility to maintain contact with study participants, to remind them of their scheduled clinic visits and to document any travel outside the area of Bandiagara.

7.1.5 Detailed description of study visits

Day -35 to –1 Screening /inclusion of participants

Meetings will be held with city administrative and medical authorities to explain the purpose of the study. These will be followed by meetings with the traditional authorities and the heads of families for village-level "permission to enter." Subsequently, general information about the study will be disseminated through the local radio station. Target population will be invited for screening as described in investigator's subject recruiting SOPs. Screening will be performed until 40 volunteers are included.

Visit 1 (may take place over more than one visit)

- Written informed consent for screening
- Assignment of participant ID number
- Medical history of participant
- Physical examination of all body major systems: Ear-Nose-Throat, Pulmonary, Cardiovascular, Musculoskeletal, Central Nervous, Renal, Gastro-intestinal and Skin. All findings will be recorded on screening forms.
- Collect 5-10 ml venous blood sample to measure:
  - Hematology: Complete Blood Count (CBC) which includes: red blood cells, platelets, white blood cell count, lymphocyte count, hemoglobin, mean corpuscular volume and mean corpuscular hemoglobin concentration.
  - Biochemistry: serum creatinine and ALT
- Beta-HCG pregnancy test on urine for females
- Collect urine: dipstick for blood, glucose and protein.
- Check of inclusion and exclusion criteria
- Prepare an ID card containing participant's unique study number and photo for enrolled participants.

Day 0: Vaccination 1
Visit 2

Before vaccination:

- Review screening laboratory test results
- Review inclusion/exclusion criteria and check of contraindications/precautions
- Written informed study consent for vaccination
- Randomization and assignment of randomization number
- Record any complaints, symptom-directed physical examination, and examination of the immunization arm(s) for any abnormalities.
- Record vital signs: oral temperature, blood pressure, pulse
- Record baseline data for solicited general symptoms
- Collect 5-10 ml venous blood sample to measure:
  - CBC, hemoglobin electrophoresis, creatinine, ALT
  - Serum for anti-MSP1 antibody titer (store at $\leq$-20°C)
- Collect urine for β-HCG test for females
- Confirm that the participant's study number agrees with the label on syringe.
- Administer study vaccine dose 1; record date and time of injection

After vaccination:

- Observe for a minimum of 30 minutes
- Record blood pressure, pulse, oral temperature
- Record solicited and unsolicited events
- Instruct participants to return to the BMP clinic center immediately should they manifest any signs or symptoms they perceive as serious.

Days 1-3, 7: First week post-vaccination 1 follow-up visits
Visit 3-6

- Record vital signs; blood pressure, pulse, oral temperature
- Examine site of injection
- Record solicited and unsolicited signs/symptoms
- Targeted physical examination including immunization arm(s)

Day 14± 3 days: 14 days post-vaccination 1 follow-up visit
Visit 7

- Brief medical history
- Record vital signs; blood pressure, pulse, oral temperature
- Physical examination
- Record any unsolicited adverse events occurring after the last vaccine dose
- Collect 5-10 ml of venous blood to measure:
    - Serum for anti-MSP1 antibodies (store at ≤-20°C)
    - CBC, creatinine and ALT

Day 30± 7 days: 1 month post-vaccination follow-up visit and vaccination 2
Visit 8

Before vaccination:

- Check participant’s ID to confirm identity
- Targeted physical examination including immunization arm(s)
- Check of contraindications/precautions
- Record vital signs: oral temperature, blood pressure, pulse
- Review medical history and record any unsolicited adverse events occurring since last visit
- Record baseline data for solicited general symptoms
- Collect 5-10 ml venous blood sample to measure:
    - CBC, creatinine, ALT
    - Serum for anti-MSP1 antibody titer (store at ≤-20°C)
- Collect urine for β-HCG test for females
- Confirm that the participant’s study number agrees with label on syringe.
- Administer study vaccine dose 2; record date and time of injection

After vaccination:

- Observe for at least 30 minutes
- Record blood pressure, pulse, oral temperature
- Record solicited and unsolicited adverse events
- Instruct participants to return to the BMP clinic center immediately should they manifest any signs or symptoms they perceive as serious.

Days 31-33, 37 ± 7 days: Days 1,2,3,7 post-vaccination 2 follow-up visits
Visit 9-12

- Record vital signs; blood pressure, pulse, oral temperature
- Examine site of injection
- Record daily solicited and unsolicited signs/symptoms
- Targeted physical examination including immunization arm(s)

Day 44± 7 days: 14 days post-vaccination 2 follow-up visit
Visit 13

- Brief medical history
- Record vital signs; blood pressure, pulse, oral temperature
- Physical examination
- Record any unsolicited adverse events occurring after the last vaccine dose
- Collect 5-10 ml of venous blood to measure:
    - Serum for anti-MSP1 antibodies (store at ≤-20°C)
    - CBC, creatinine and ALT

Day 60± 7 days: 1 month Post-vaccination 2 follow-up and vaccination 3
Visit 14

Before vaccination:

- Check participant's ID to confirm identity
- Targeted physical examination including immunization arm(s)
- Check of contraindications/precautions
- Record vital signs: oral temperature, blood pressure, pulse
- Review medical history and record any unsolicited adverse events occurring since last visit
- Record baseline data for solicited general symptoms
- Collect 5-10 ml venous blood sample to measure:
    - CBC, creatinine, ALT
    - Serum for anti-MSP1 antibody titer (store at ≤-20°C)
- Collect urine for β-HCG test for females
- Confirm that the participant's study number agrees with label on syringe.
- Administer study vaccine dose 3; record date and time of injection

After vaccination:

- Observe for at least 30 minutes
- Record blood pressure, pulse, oral temperature
- Record solicited and unsolicited adverse events
- Instruct participants to return to the BMP clinic center immediately should they manifest any signs or symptoms they perceive as serious.

Days 61-63, 67 ± 7 days: Days 1,2,3,7 post-vaccination 3 follow-up visits
Visit 15-18

- Record vital signs; blood pressure, pulse, oral temperature
- Examine site of injection
- Record daily solicited and unsolicited signs/symptoms
- Targeted physical examination including immunization arm(s)

Day 74± 7 days: 14 days Post-vaccination 3 follow-up visit
Visit 19

- Brief medical history
- Record vital signs; blood pressure, pulse, oral temperature
- Targeted physical examination including immunization arm(s)
- Record any unsolicited adverse events occurring after the last vaccine dose
- Collect 5-10 ml of venous blood to measure:
  - Serum for anti-MSP1 antibodies (store at ≤-20°C)
  - CBC, creatinine and ALT

Day 90± 10 days: 30 days Post-vaccination 3 follow-up visit
Visit 20

- Brief medical history
- Record vital signs; blood pressure, pulse, oral temperature
- Targeted physical examination including immunization arm(s)
- Record any unsolicited adverse events occurring since last visit
- Collect 5-10 ml of venous blood to measure:
  - Serum for anti-MSP1 antibodies (store at ≤-20°C)
  - CBC, creatinine and ALT
- Collect urine for β-HCG test for females

Day 120± 10 days to Day 364± 10 days:
Post-vaccination Safety surveillance period

- From this date, participants will be visited monthly by local guides to confirm their location and to remind them to come to clinic for routine clinical evaluation.
- Participants are invited to continue to attend the BMP clinic any time they are sick. A malaria smear will be done whenever symptomatic malaria is suspected.

Day 180± 14 days to Day 364± 14 days:
Post-vaccination Safety surveillance period
Visit 21-23

- From this day participants will be asked to return to BMP clinic center every 3 months ±14 days.
- Targeted physical examination including immunization arm(s)
- Collect 5-10 ml venous blood to measure:
  - Serum for anti-MSP1 antibodies (store at ≤ -20°C)
  - CBC, ALT, creatinine
- Collect urine for β-HCG test for females
- Participants are invited to continue to attend the BMP clinic any time they are sick. A malaria smear will be done whenever symptomatic malaria is suspected.

7.1.6 Outline of study procedures

**Study Days**<br><br>**Clinic Visit**	**-35 to –1 Screening**<br>**1**	**0**<br>**2**	**1-3, 7**<br>**3-6**	**14**<br>**7**	**30**<br>**8**	**31-33, 37**<br>**9-12**	**44**<br>**13**	**60**<br>**14**	**61-63, 67**<br>**15-18**	**74**<br>**19**	**90**<br>**20**	**120-364**<br>**21-23**
Village and family level information and discussion	●											
Written individual Screening Consent	●											
Check of inclusion/exclusion criteria	●	●										
Written individual Study Consent		●										
Medical history	●	●	●	●	●	●	●	●	●	●	●	●
Targeted physical examination	●	●	●	●	●	●	●	●	●	●		
Vital signs (T, BP, P)	●	●[f]	●	●	●[f]	●	●	●[f]	●	●	●	
**Vaccination**		●			●			●				
Post-vaccination recording of solicited AE			●			●			●			
Recording of unsolicited AE occurring one month (minimum 30 days) post-vaccination, by investigators			●	●	●	●	●	●	●	●	●	
Recording of medication		●		●	●		●	●		●	●	
Recording of SAEs during the study period		●	●	●	●	●	●	●	●	●	●	●
Urine analysis for blood, glucose and protein	●[a]											
Urine β-HCG	●[a]	●[b]			●[b]			●[b]			●	●[d]
CBC	●[a]	●[c]		●	●[c]		●	●[c]		●	●	●[d]
Serum chemistry (Creatinine, ALT)	●[a]	●[c]		●	●[c]		●	●[c]		●	●	●[d]
Serum for anti-MSP1 response		●[c]		●	●[c]		●	●[c]		●	●	●[e]
Monthly home visit by local guides												●[h]
Review of health status												●[i]
Scheduled blood volume (ml)	5-10	5-10	0	5-10	5-10	0	5-10	5-10	0	5-10	5-10	15-30
Cumulative Blood Volume (ml)	5-10	10-20	10-20	15-30	20-40	20-40	25-50	30-60	30-60	35-70	40-80	55-110

a. Performed within 35 days prior to immunization
b. Performed just prior to each immunization
c. Blood collected just prior to each immunization
d. CBC, creatinine, ALT and urine β-HCG will be determined every 3 months from study Day 180 through study Day 364
e. Serum for MSP1 antibodies collected every 3 months from study Day 180 through study Day 364
f. Pre-dose and 30mn after each dose
g. Performed each time symptoms/signs evoking clinical malaria are present
h. Monthly local guides home visits to check the status of participants starting from study Day 120
i. Record any new onset chronic or acute diseases or other medically significant conditions, unscheduled clinic visits and any new treatments since previous scheduled study visit.

Table 2: Intervals between study visits

**Interval**	**Size of interval in days**
Visit 2 → Visit 7	14 ± 3
Visit 2 → Visit 8	30 ± 7
Visit 8 → Visit 13	14 ± 3
Visit 8 → Visit 14	30 ± 7
Visit 14 → Visit 19	14 ± 3
Visit 14 → Visit 20	30 ± 7

7.2 Definition and Management of Clinical Malaria

7.2.1 Definition of clinical malaria

A clinical episode of *P. falciparum* malaria is defined as the presence of *P. falciparum* asexual parasitemia on Giemsa-stained thick blood smear films in the presence of the following: (i) fever defined as oral temperature $\geq$ 37.5°C in the absence of other evident clinical conditions that could explain the fever; and/or (ii) one or more of the following symptoms consistent with malaria including but not limited to headache, vomiting, abdominal pain, or myalgia with or without fever. This case definition is based on six continuous years of clinical experience treating children and adults with malaria in Bandiagara, and represents the current standard for clinical decisions to treat malaria in this setting. Study investigators will use their best clinical judgment in deciding when to treat malaria and will not be precluded from treating malaria by this definition.

7.2.2 Treatment of malaria clinical episodes during the study

Chloroquine, the first-line antimalarial drug recommended by the Malian National Malaria Control Program, will be used to treat clinical episodes of uncomplicated malaria at the standard dose of 25 mg/Kg of body weight over 3 days: 10 mg/Kg on the first and second days and 5 mg/Kg on the third day of treatment. Despite reports of chloroquine-resistant *P. falciparum* malaria elsewhere in West Africa, it is still highly efficacious in Bandiagara for malaria treatment. Our recent studies have found adequate clinical response rates of ~91% among children in Bandiagara. Because of their semi-immune status we can expect a better clinical efficacy rate among the adult population.

Chloroquine use may be limited in some cases by the occurrence of pruritus, which was reported by 21% of chloroquine users in Bandiagara in 1998. In case of intolerance to chloroquine or in case of therapeutic failure, sulfadoxine-pyrimethamine (SP), the second-line drug recommended by the Malian National Malaria Control Program will be used, administered as a single dose of 3 tablets. SP is well tolerated and the potential issue of hemolysis due to G6PD deficiency has never been observed in the Malian population despite widespread use of this drug for many years in Mali. SP-related hemolysis has not been reported in several African countries that use SP as a first-line drug, and is attributable to the form of G6PD deficiency that is found in sub-Saharan Africa being a much milder form than that seen in Mediterranean populations. The rate of adequate clinical response to SP in Bandiagara according to studies conducted in 1999 and 2000 is more than 95%.

In the highly unlikely event of SP failure, a standard 7-day course of quinine will be administered as recommended by the WHO and the Mali National Control Programme.

All treatment courses will be administered under the supervision of the investigators and recorded in the appropriate section of the CRF. Participants diagnosed with clinical malaria will be treated before any further immunization. Participants evaluated in this manner will be given the appropriate dose of vaccine if their clinical symptoms resolve within 7 days. If the clinical symptoms do not resolve within 7 days the participants will not be vaccinated. However they will be followed for safety and immunogenicity. If any other illness is revealed during the malaria episode evaluation, such as an upper respiratory infection, the participants will receive appropriate care from the BMP physician team. If further evaluation or treatment is required, the participant will be referred to the Regional Hospital of Mopti or the National Hospital in Bamako, where they will be managed according to local standards of care. Transportation will be provided. Senior study investigators and the medical monitor will monitor their clinical management to assure that appropriate care is received.

Participants will be followed until resolution of symptoms.

8 SAMPLE HANDLING AND ANALYSIS

8.1 Overview of collection time points

Blood will be collected from study participants by venipuncture up to 11 times during the study. The maximum amount of blood requested from any participant for standard collection during the study for research purposes will not exceed 55-110 ml. However, additional blood may be obtained as deemed necessary by the investigators or clinicians to evaluate any illness or condition.

1. Safety

Test for CBC, creatinine, and ALT, at screening, Day 0, Days 14, 30, 44, and 60, and Days 74, 90 and every three months thereafter will be performed at the BMP clinical laboratory.

2. Serology

Separation of serum/plasma from the venous blood will be performed at the BMP clinic and samples (approx. 1-2 ml) will be aliquoted for later use to determine anti-MSP1 antibody levels at Day 0, Days 14, 30, 44,and 60 and Days 74, 90 and every three months thereafter until the end of the study.

8.2 Handling of biological samples collected by the Investigators

8.2.1 Instructions for handling of serum samples

1. Collection

Venous blood will be collected observing appropriate aseptic conditions. Serum will be collected whenever possible using Vacutainer® tubes with integrated serum separator (e.g. Becton-Dickinson Vacutainer® SST or Corvac® Sherwood Medical) or serum microtainers so as to minimize the risk of hemolysis and to avoid blood cell contamination of the serum when transferring to standard serum tubes. Plasma samples will be collected from EDTA or heparinized blood following centrifugation at 200 g for 3 minutes.

2. Serum separation

These guidelines aim to ensure high quality serum by minimizing the risk of hemolysis, blood cell contamination of serum or serum adverse cell toxicity at testing.

- For separation using Vacutainer® tubes, the instructions provided by the manufacturer will be followed.
- Following separation, the serum will be transferred to the appropriate standard tubes using a disposable pipette. The serum will be transferred as gently as possible to avoid cell contamination.
- The tubes will not be overfilled (max. 3/4 of the total volume) to allow room for expansion upon freezing.
- An appropriate cryo-resistant label will identify the tube.

8.2.2 Labeling

- Standard cryolabels will be used to label each serum or cell sample. Each label will contain the protocol number, the participant study number, the date of collection and specimen type.
- If necessary, any hand-written additions to the labels will be made using indelible ink.
- The label will be attached to the tube as follows: First attach the blank end of the label to the tube and then wrap the label around the tube so that the opposite end of the label overlaps the blank end ensuring that no written portion is covered.
- Labels will not be attached to caps
- Serum tubes will be stored in a vertical position at a temperature ≤ - 20ºC.

8.3 Laboratory Assays

The Investigators will maintain detailed SOP's on all laboratory assays at the BMP and central laboratories at MRTC, in Bamako. The methods that will be used are outlined in the following.

Hematology and Biochemistry:

A complete blood count (CBC), serum creatinine and ALT tests will be measured at defined time points throughout the study period. The Principal Investigator will maintain laboratory reference values and copies will be made available upon request to study monitors and sponsors.

- Complete blood count will be done using a Coulter AcT–series instrument.
- Serum creatinine will be determined using the Roche Reflotron Plus instrument.
- Serum alanine aminotransferase (ALT) using the Roche Reflotron Plus instrument.

Hematology and serum biochemistry assays will be performed at the BMP clinical laboratory in Bandiagara.

Urine will be collected for glucose, blood and protein determination using FDA-approved urinary reagent dipsticks. Pregnancy tests will be performed for women on urine samples using FDA-approved urine pregnancy test kits.

Procedure for the Laboratory Diagnosis of Malaria

If a participant is suspected of having symptoms of malaria, blood smears will be made and examined while the volunteer is in the BMP clinic. The blood will be collected by finger prick. Duplicate thick smears will be made. The slides will be placed horizontally, protected from flies and allowed to dry at room temperature with the aid of a fan if necessary. Once dry, the slides will be placed upright in a slide holder. The smears will be introduced in a staining rack and stained for thirty minutes in a 5% solution of Giemsa in phosphate buffer (pH 7.0-7.2). The slides will be then washed to remove excess stain and returned to slide holder and then dried at room temperature. All thick smears will be stained and reviewed according to the investigator's SOP.

Serology (Antibody responses):

Serological assays will be performed at the WRAIR laboratories in Silver Spring, US for antibody determination. Serum will be collected at indicated time points (see section 8.1).

Blood for analysis of antibody responses will be obtained from each participant and allowed to clot for one hour at room temperature, and serum will be separated and frozen at ≤ -20ºC until tested. All samples will be labeled with the participant's study number and date of collection, as previously described.

Immunogenicity (antibody levels) will be determined by evaluating antibody (IgG) responses to the *P. falciparum* $MSP1_{42}$ as measured using standard ELISA methodologies with appropriate capture antigens.

Additional assays

As a capacity-building exercise, WRAIR investigators will assist MRTC investigators with establishing the ability to perform serological assays for antibody (IgG) responses to the *P. falciparum* $MSP1_{42}$ by using the same ELISA methodologies with appropriate capture antigens that will be used for the immunogenicity study endpoint at WRAIR.

No additional blood will be drawn for any of these capacity-building assays. After final serological results from the reference immunology laboratory at WRAIR have been fully analyzed and reported, the results of the Mali assays may be compared to the WRAIR serological results, solely for the purposes of assessing how well the Malian laboratory was able to replicate the WRAIR results. It is emphasized that this is a capacity-building exercise and that only the serological results from the WRAIR Department of Immunology will be analyzed as trial endpoints.

9 STUDY VACCINES/MEDICATIONS AND ADMINISTRATION

9.1 Study vaccines

9.1.1 FMP1 vaccine

The candidate vaccine has been developed and manufactured by the WRAIR. The adjuvant AS02A is manufactured by GSK. The Quality Control Standards and Requirements for each component of the vaccine are described in separate release protocols and the required approvals have been obtained.

9.1.1.1 Active ingredients:

FMP1

62.5 µg of lyophilized protein with 3.1% lactose as cryoprotectant per vial.

AS02A adjuvant

The FMP1 vaccine will be reconstituted in AS02A adjuvant. AS02A contains 50 µg MPL and 50 µg QS21, 250 µl of SB62 (oil/water emulsion) in phosphate buffered saline (PBS) per volume of 0.5 ml. All AS02A vials contain 0.65-0.75 ml of liquid and will be stored at 2ºC to 8ºC. As opposed to the AS02 used in previous studies, the AS02A adjuvant contains no thiomerosal. AS02A adjuvant will be supplied as pre-filled syringe.

9.1.2 Imovax® Rabies vaccine

The rabies vaccine, Imovax® Rabies, manufactured by Aventis Pasteur, SA is a sterile, stable, freeze-dried suspension of rabies virus prepared from the strain PM-1503-3M obtained from the Wistar Institute, Philadelphia, PA. Each 1 ml dose of reconstituted vaccine contains 100 mg of human albumin, less than 150 µg of neomycin and equal or greater than 2.5 IU of rabies antigen. The potency of the final product is determined by the NIH mouse potency test using the US reference standard. The vaccine is supplied as single dose vials containing lyophilized antigen with 1 ml of diluent in a pre-filled syringe (water for injection).

9.2 Dosage and administration

9.2.1 Reconstitution of FMP1 vaccine

The top of a lyophilized FMP1 vaccine vial will be disinfected with alcohol swabs and allowed to dry for a few seconds. The AS02A contents of one pre-filled syringe will be injected using a sterile needle into a vial of lyophilized vaccine. The needle and syringe will be discarded. The pellet of FMP1 will then be dissolved by gently swirling the vial and waiting for 1 minute to ensure complete dissolution of vial contents before withdrawing 0.5 ml of reconstituted FMP1 into a sterile 1 ml syringe with a 1" 23 gauge needle (See also section 9.5 for instructions on masking the syringe contents).

9.2.2 Reconstitution of Imovax ® Rabies vaccine

The top of a vaccine vial will be disinfected with alcohol swabs and allowed to dry a few seconds. The complete contents of a pre-filled syringe containing diluent (1 ml of water for injection) will be injected into a vial of lyophilized vaccine. The pellet will then be allowed to dissolve by gently swirling the vial and waiting for 1 minute to ensure complete dissolution of vial contents before withdrawing 1 ml of the reconstituted rabies vaccine with a sterile 1 ml syringe and a 1" 23 gauge sterile needle (See also section 9.5 for instructions on masking the syringe contents).

9.2.3 Administration of vaccines

Each 0.5 ml of FMP1 or 1.0 ml of Imovax® Rabies vaccine will be administered slowly by intramuscular injection in the left deltoid muscle immediately after reconstitution. Alternatively the right deltoid muscle could be used when the preferred site for injection is contraindicated or not advisable such as in the case of severe pain, infection or if the study participant declares a preference to be immunized in the alternative site.

The vaccinees will be observed closely for at least 30 minutes, with appropriate medical treatment readily available in case of a rare anaphylactic reaction following the administration of vaccines.

Senior physician investigators trained in the management of acute anaphylaxis reactions will administer the vaccines in the BMP clinic. The vaccinating investigators will not be directly involved in post-immunization assessment of adverse events. A physician skilled and familiar with emergency resuscitation procedures will assist during the immunization phases. In order to maintain the study blinding, the vaccines will be prepared for administration by a specific team that will not be involved in further participant evaluation during follow-up.

9.3 Storage

ALL VACCINES MUST BE STORED AT A TEMPERATURE BETWEEN + 2ºC AND + 8ºC (IN A REFRIGERATOR OR COOLER) AND MUST NOT BE FROZEN. ALL AS02A PRE-FILLED SYRINGES MUST BE STORED AT + 2ºC TO + 8ºC.

Vaccines will be kept in an exclusively dedicated refrigerator that has 24-hour temperature recording. A back-up refrigerator and generator will be available in case of breakdown/power failure. The refrigerator that holds the vaccines and adjuvant will be maintained locked. The field site manager and the study coordinator will keep the keys. Records will be maintained that document receipt, release for immunization, disposal or return to the manufacturer of all vaccine vials. Copies of these records will be provided to the sponsors for archiving.

9.4 Treatment allocation and randomization

Individual participants will be randomized to receive either FMP1 or Imovax® Rabies vaccine without stratification for gender. The gender distribution in the sample is expected to be representative of the gender distribution in the global population of Bandiagara. The randomization list will contain sequential codes linked to a study vaccine assignment (FMP1 or Rabies). The codes will be assigned to participants in the order in which they present to the clinic on the first day of immunization. The access to the randomization list will be exclusively limited to the study drug manager(s)/pharmacist(s). These individuals are unblinded and will not be involved in study participants' further evaluation. It is critical that they understand the importance of not revealing the contents of the randomization list to anyone else involved in the study. The Local Medical Monitor will also keep one set of the randomization code in a sealed envelope in the event that emergency unblinding is required.

9.5 Methods of Blinding and Breaking the Study Blind

9.5.1 Blinding

The reconstituted FMP1 vaccine and the AS02A diluent will have exactly the same milky white appearance. The FMP1 vaccine and the AS02A diluent will be packaged separately. The comparator rabies vaccine will be in the same package as received from the manufacturer. After reconstitution it will appear clear pink. Therefore, blinding of the individual preparing the vaccine dose ("drug manager") will not be possible. Since the test article and comparison vaccines can be distinguished by appearance, the vaccine preparation area and the immunizing area will be physically separated. The drug manager, an experienced pharmacist, will be exclusively dedicated to vaccine preparation. He may have assigned to him a drug manager assistant to ensure that the proper vaccine is delivered for each participant. To determine which vaccine each participant will receive, the drug manager will refer to the unique randomization code assigned to that participant. The drug manager will check the participant number on the participant's photo ID and will make sure that it matches that in the CRF. The drug manager will then refer to a key matching the randomization code given to the participant to the vaccine to be administered. He will also confirm that the randomization code of the participant matches the vaccine to be given in the key list. The vials will be reconstituted as above and the vaccine will be drawn into a 1 ml syringe. The barrel of the syringe will be covered with opaque tape to hide its contents and labeled with

the participant number. The syringe will be labeled with a sticker containing the randomization code for the individual and the individual's ID number.

Immunizations will be carried out simultaneously in one or two separated rooms close to the vaccine preparation room. The vaccine-filled syringes will be brought to vaccinators who will consist of co-investigators who will not be involved with follow-up assessments. They will maintain, according to the Investigators' SOPs, a vaccine log record that will contain the participant ID number, the randomization code from the randomization list and the randomization code from the syringe containing the vaccine. At the end of each immunization period, the vaccination log will be handed to the drug manager who will confirm the absence of discrepancies and will keep all the vaccination logs and the randomization key in a locked cabinet.

The PI will maintain in his study file SOPs describing all blinding and immunization procedures.

Despite the fact that the volumes of the two study vaccines are different, every attempt will be made to maintain blinding. Firstly, the syringe barrels will be covered with opaque tape. Secondly, the injectors of the study vaccines will be investigators who are not involved in any way with follow-up activities, so that even if they realize which vaccine they are injecting, they will not be involved in the assessment of adverse events following vaccination. WRAIR investigators and many others who have used this technique concede that a very astute participant could discern how far syringe plungers extend from the barrel, and potentially discuss this later with other participants. Mitigating this concern is the practice of injecting each participant in a closed room with only the injector present, so that each participant sees only the syringe he or she is injected with and never sees other participants being injected. Furthermore, the participants are not told that the two vaccines vary with respect to volume, so it is very unlikely that they will discuss this amongst themselves. It would theoretically be possible to use a curtain or some other obstruction so that the participant never sees the syringe. However, such an unorthodox procedure would be very anxiety-provoking for participants and is unjustified.

9.5.2 Breaking the study blind

A participant's study randomization code may be unblinded only for safety purposes. This is very unlikely to occur, as once a vaccine is administered, knowing which vaccine was given is unlikely to influence the medical management of an adverse event. This procedure is therefore exceptional and any decision to unblind will be discussed with the sponsors, the PI, the Senior Co-Investigators, the Local Medical Monitor, SMC and the DSMB. If deemed necessary for urgent safety reasons, the Local Medical Monitor, in consultation with the SMC, may unblind a specific participant without revealing the study blind to the investigators and the sponsors. Any opening of these coded envelopes will be documented according to investigator SOPs. It is to be emphasized that the Local Medical Monitor and SMC may put the study on hold at any time and discuss with the DSMB. The DSMB will consult with the investigators and sponsors as necessary. The decision to completely unblind or permanently stop the study, will take the final form of a formal recommendation by the DSMB to the study sponsor and IND sponsor. The PI must then notify the local IRB of this decision.

In the event that the investigators come to know the study code, the PI must notify the sponsors immediately. The reasons will be documented by the PI and added to the study file.

Final unblinding will be done only after final closeout monitoring/verification of GCP compliance by USAMMDA, DSMB review to verify all safety concerns have been addressed, and after all safety and immunological results have been entered and databases

locked. The final decision to unblind will be made jointly by USAMMDA, DMID and the DSMB.

9.6 Replacement of unusable vaccine doses

In addition to study vaccines, 5% supplemental single doses of AS02A will be provided to replace broken or lost doses. All the procedures described in Section 7 still apply.

9.7 Vaccine accountability

The FMP1 vaccine will be transported by WRAIR personnel to the University of Maryland following SOPs for maintaining and documenting required temperature ranges. Both vaccines will be shipped hand-carried to Mali by CVD personnel including the study co-PI, and temperature recorders will document maintenance of required temperature ranges. The AS02A adjuvant will be similarly shipped from Belgium. On arrival in Bamako, these shipments will have been pre-cleared with Customs and will be retrieved directly after flight arrival by the PI and the vaccine manager. Vaccines and adjuvant will be stored in a cold room in the main MRTC laboratory in Bamako until a few days before each vaccination is scheduled in Bandiagara. Vaccines will be transported in an air-conditioned vehicle from Bamako to Bandiagara in the same containers used for shipping to Mali. Only the vaccine manager and assistant vaccine manager will have access to vaccines at all times. The Vaccine Log Book will also be used to record use and final disposition of each vial of vaccine and adjuvant. Used vaccine vials, as well as unused vaccine vials, will be kept , until such time as the investigators and sponsors agree that there are no concerns about vaccine accountability and that they can be discarded.

9.8 Concomitant medication/Treatment

At each study visit/contact, the investigator will question the participant about any medication taken. Concomitant medication, including any vaccine other than the study vaccines, and any other medication relevant to the protocol, including any specifically contraindicated or administered during the period starting from one week before each dose and ending one month (maximum 30 days) after must be recorded in the case report form with trade name and/or generic name of the medication, medical indication, start and end dates of treatment.

9.8.1 Drugs to treat anaphylaxis

These include epinephrine 1:1,000, epinephrine 1:10,000, diphenhydramine and methylprednisolone. Epinephrine will be injected parenterally in standard recommended doses. Diphenhydramine will be administered orally or parenterally in doses of 50 mg. Methylprednisolone will be injected intravenously in doses of 10-40 mg as needed to treat anaphylaxis. A kit for anaphylaxis management including required drugs, necessary supplies for airway management and oxygen will be available on-site. The Investigators will be trained and familiarized with common resuscitation procedures. A physician skilled and familiar with emergency cardiac resuscitation will assist on-site at each immunization phase.

9.8.2 Drugs to treat malaria

Chloroquine (CQ) will be the first line drug for malaria treatment. In case of therapeutic failure to CQ, Sulfadoxine-Pyrimethamine (SP) or quinine will be used as recommended by Malian National Malaria Control Program.

10 ADVERSE EVENTS

It is the responsibility of the investigators to document all adverse events according to the detailed guidelines set out below. The participants will be instructed to contact the investigator immediately should they manifest any signs or symptoms that they perceive as serious during the study.

10.1 Eliciting and documenting adverse events

10.1.1 Adverse event definition

An adverse event includes any noxious, pathological or unintended change in anatomical, physiological or metabolic functions as indicated by physical signs, symptoms and/or laboratory detected changes occurring in any phase of the clinical study whether associated with the study vaccine, active comparator or placebo and whether or not considered vaccination related. This includes an exacerbation of pre-existing conditions or events, intercurrent illnesses, or vaccine or drug interaction. Anticipated day-to-day fluctuations of pre-existing conditions that do not represent a clinically significant exacerbation need not be considered adverse events. Discrete episodes of chronic conditions occurring during a study period will be reported as adverse events in order to assess changes in frequency or severity.

Adverse events will be documented in terms of a medical diagnosis. When this is not possible, the adverse event will be documented in terms of signs and/or symptoms observed by the investigator or reported by the subject at each study visit.

Pre-existing conditions or signs and/or symptoms (including any which are not recognized at study entry but are recognized during the study period) present in a participant prior to the start of the study will be recorded on the Medical History form within the participant's CRF. Any of the signs or symptoms to be solicited present during physical examination of the participant at each vaccination visit will be recorded on the Pre-vaccination assessment page of the participant's CRF.

Adverse events, which occur after informed study consent is obtained, but prior to vaccination, will be documented in the Medical History form within the subject's CRF

Any hospitalization will be considered a serious adverse event. However, hospitalization for either 1) elective surgery related to a pre-existing condition that did not increase in severity or frequency following initiation of the study, or 2) for routine clinical procedures that are not the result of an adverse event, need not be considered as adverse events and are therefore not serious adverse events.(See section 10.5).

Adverse events to be recorded as endpoints are described in Section 10.1.4 All other adverse events will be recorded as unsolicited adverse events.

10.1.2 Surveillance period for occurrence of adverse events

All adverse events occurring within 31 days following administration of each dose of vaccine must be recorded on the Adverse Event form in the participant's CRF, irrespective of severity or whether or not they are considered vaccination-related.

Solicited adverse events will be elicited for an 8-day follow-up period (day of vaccination and days 1, 2, 3 and 7 after vaccination) and unsolicited adverse events will be recorded during a 31-day follow-up period (day of vaccination and 30 subsequent days). Serious adverse events will be recorded throughout the study.

Instances of death, cancer or congenital abnormality in offspring of a study subject if brought to the attention of the investigator AT ANY TIME after cessation of study AND suspected by the investigator to be related to study vaccine, will be reported to the sponsors and GSK Biologicals.

10.1.3 Recording adverse events

At each visit/assessment, the investigator will evaluate all adverse events either observed by the investigators or reported by the participant spontaneously or in response to a direct question. New adverse events will be recorded in the Adverse Event form within the participant's CRF. The nature of each event, date and time (where appropriate) of onset, outcome, intensity and relationship to vaccination will be established. Details of any corrective treatment will be recorded on the appropriate page of the CRF. See Section 10.5 for instructions for reporting and recording of serious adverse events.

As a consistent method of soliciting adverse events, the participant will be asked a non-leading question such as: "Have you felt different in any way since receiving the vaccine or since the last visit?" The investigator will record only those adverse events having occurred within the time frames defined above.

Adverse events already documented in the CRF, *i.e.* at a previous assessment and designated as 'ongoing' will be reviewed at subsequent visits, as necessary. If these have resolved, the documentation in the CRF will be completed. If an adverse event changes in frequency or intensity during a study period, a new record of the event will be started.

10.1.4 Solicited adverse events

Local (injection site) adverse events
Pain at injection site
Swelling at injection site
Erythema
Limitation of arm motion (abduction at the shoulder)

General adverse events
Fever (oral body temperature ≥ 37.5°C)
Chills
Nausea
Headache
Malaise
Myalgia
Joint pain

Temperature will be recorded at the time of the clinic visit. If additional temperature measurements are recorded at another time of the day, the highest temperature will be recorded.

The assessment of severity/intensity will be as described in Section 10.2. For general signs and symptoms reported, the investigators will assign causality as described in Section 10.3 For all signs and symptoms reported, the investigators will report the outcome as described in Section 10.4.

10.1.5 Unsolicited adverse events

Unsolicited adverse events will be recorded in dedicated space within the CRF. Unsolicited adverse events are adverse events reported by the participants that are different from those solicited or solicited symptoms that begin after the 8-day follow-up period for solicited adverse events. Should any systemic (general) signs/symptoms be reported, their relationship with the study vaccine will be assessed by the investigators and transcribed into the CRF, as descried in section 10.3.

10.2 Assessment of intensity

For each solicited symptom the participants will be asked if they sought medical advice for this symptom.

For all other adverse events than those in Table 3, maximum intensity will be assigned to one of the following categories:

0 = No adverse event

1 = An adverse event which is easily tolerated by the subject, causing minimal discomfort and not interfering with everyday activities.

2 = An adverse event that is sufficiently discomforting to interfere with normal everyday activities.

3 = An adverse event that prevents normal, everyday activities. Such an adverse event would for example prevent attendance at work/school and would require the administration of corrective therapy.

Intensity of the following adverse events will be assessed as described in Table 3:

Table 3: Assessment of adverse event intensity

Adverse Event	Intensity grade	Intensity definition
Pain at injection site	0	None
	1	Pain that is easily tolerated
	2	Pain that interferes with daily activity
	3	Pain that prevents daily activity
Swelling at injection site<br>Record size	0<br>1<br>2<br>3	0 mm<br>>0 - ≤ 20 mm<br>>20 - ≤ 50 mm<br>>50 mm
Erythema at injection site<br>Record size	0<br>1<br>2<br>3	0 mm<br>>0 - ≤ 20 mm<br>>20 - ≤ 50 mm<br>>50 mm
Limitation of arm motion - Abduction at the shoulder	0	None
	1	>90° but <120°
	2	>30° but ≤90°
	3	≤30°
Fever<br>Record oral temperature	0<br>1<br>2<br>3	<37.5ºC<br>37.5 - ≤38.0ºC<br>>38.0 - ≤39ºC<br>>39ºC
Chills	0	None
	1	Chills that are easily tolerated
	2	Chills that interfere with daily activity
	3	Chills that prevent daily activity
Nausea	0<br>1<br>2<br>3	None<br>Nausea that is easily tolerated<br>Nausea that interferes with daily activity<br>Nausea that prevents daily activity
Headache	0	None
	1	Headache that is easily tolerated
	2	Headache that interferes with daily activity
	3	Headache that prevents daily activity
Malaise	0	None
	1	Malaise that is easily tolerated
	2	Malaise that interferes with daily activity
	3	Malaise that prevents daily activity
Myalgia	0	None
	1	Myalgia that is easily tolerated
	2	Myalgia that interferes with daily activity
	3	Myalgia that prevents daily activity
Joint pain	0	None
	1	Joint pain that is easily tolerated
	2	Joint pain that interferes with daily activity
	3	Joint pain that prevents daily activity

10.3 Assessment of causality

Every effort will be made by the investigator to explain each adverse event and assess its causal relationship, if any, to administration of the study vaccine(s).

The degree of certainty with which an adverse event can be attributed to administration of the study vaccine(s) (or alternative causes, e.g., natural history of the underlying diseases, concomitant therapy, etc.) will be determined by how well the event can be understood in terms of one or more of the following:

- Reaction of similar nature having previously been observed with this type of vaccine and/or formulation.

- The event having often been reported in literature for similar types of vaccines.
- The event being temporally associated with vaccination or reproduced on re-vaccination.

All solicited local (injection site) reactions will be considered causally related to vaccination. Causality of all other adverse events will be assessed by the investigators using the following method:

In your opinion, did the vaccine(s) possibly contribute to the adverse event?

NO : The adverse event is not causally related to administration of the study vaccine(s). There are other, more likely causes and administration of the study vaccine(s) is not suspected to have contributed to the adverse event.

YES : There is a reasonable possibility that the vaccine contributed to the adverse event.

Non-serious and serious adverse events will be evaluated as two distinct events given their different medical nature. If an event meets the criteria to be determined “serious” (see Section 10.5.1 for definition of serious adverse event), the investigator will examine it to the extent to be able to determine ALL contributing factors applicable to each serious adverse event.

Other possible contributing factors include:
- Medical history
- Other medication
- Protocol required procedures
- Lack of efficacy of the vaccine
- Erroneous administration

10.4 Following-up of adverse events and assessment of outcome

Investigators will follow up subjects with serious adverse events until the event has disappeared or until the condition has stabilized regardless of when this occurred in relation to the study conclusion. Investigators will follow up participants with non-serious adverse events until the participant completes the study. Clinically significant laboratory abnormalities, as well as any adverse event, will be followed up until they have returned to normal, or until a satisfactory explanation has been provided. Reports relative to the subsequent course of an adverse event noted for any subject must be submitted to the Study Monitor.

Outcome will be assessed as:
1 = Recovered
2 = Recovered with sequelae
3 = Ongoing at participant study conclusion
4 = Died
5 = Unknown

10.5 Serious adverse events

10.5.1 Definition of a serious adverse event

A serious adverse event is any untoward medical occurrence that results in death, is life threatening, results in persistent or significant disability/incapacity, requires in-patient hospitalization or prolongation of existing hospitalization or is a congenital anomaly/birth defect in the offspring of a study subject. In addition, important medical events that may jeopardize the patient or may require intervention to prevent one of the other outcomes listed above will be considered serious.

- Life threatening—definition: An adverse event is life threatening if the participant was at risk of death at the time of the event; it does not refer to an event, which hypothetically might have caused death, if it were more severe.

- Disabling/incapacitating—definition: An adverse event is incapacitating or disabling if the event results in a substantial disruption of the participant's ability to carry out normal life functions. This definition is not intended to include experiences of relatively minor medical significance such as headache, nausea, vomiting, diarrhea, influenza, and accidental trauma (e.g. sprained ankle).

- Hospitalization: In general, hospitalization signifies that the participant has been detained (usually involving at least an overnight stay) at the hospital or emergency ward for treatment that would not have been appropriate in the physician's office or outpatient setting.

Hospitalization for either 1) elective surgery related to a pre-existing condition that did not increase in severity or frequency following initiation of the study, or 2) for routine clinical procedures that are not the result of an adverse event, need not be considered as adverse events and are therefore not serious adverse events.

- Routine Clinical Procedure—definition: One which is defined as a procedure which may take place during the study period and will not interfere with the study vaccine administration or any of the ongoing protocol specific procedures.

If anything untoward is reported during an elective procedure, that occurrence must be reported as an adverse event, either 'serious' or 'non-serious' according to the usual criteria.

When in doubt as to whether 'hospitalization' occurred or was necessary, the adverse event will be considered serious.

10.5.2 Reporting serious adverse events

In the event that one or more serious adverse reactions probably or suspected of being related to vaccination are detected following any immunization in any of the vaccine groups, no further vaccinations will be administered until a written report has been submitted to the DSMB, DMID, U.S. Army HSRRB, University of Maryland IRB, FMPOS Ethics Review

Committee and NIAID IRB, and the investigators have conferred with the Local Medical Monitor and SMC.

Any serious adverse events occurring during the study period whether or not considered to be related to the study vaccine/comparator will be reported within 24 hours of the PI being notified to the to the IND sponsor (USAMMDA) at phone number 301-619-2165, followed by faxing of the complete AE reporting form to the Sponsor at 301-619-7803. SAEs will also be reported within 24 hours by telephone or E-mail to the Local Medical Monitor, SMC and the FMPOS Ethics Review Committee in Mali. Any SAE that is related to the study vaccine will be reported to GSK-Biologicals within 24 hours of the PI being notified. A written report will follow the initial report within 3 working days. The report will be sent to the Quality Assurance Office, USAMMDA (cf. Appendix 16.3)

Notifications and reports will be provided by USAMMDA to the following agencies by e-mail, fax or telephone within the reporting deadlines required by each agency: the co-sponsor (DMID), the DSMB, the University of Maryland School of Medicine IRB, the Food and Drug Administration, the HSRRB, the NIAID IRB, the NIAID DSMB, and GSK-Biologicals. The rationale for USAMMDA serving as the primary contact point for disseminating all SAE reports to these bodies is that communications at the field site are limited, and while every effort will be made to build redundancy into the communications systems, it is possible that phone land lines and E-mail could be down at simultaneously, leaving only satellite phones as a means of communication from Bandiagara. The PI and co-PI on site will make every effort to directly notify all IRBs, sponsors and partners directly within their required reporting deadlines, in addition to the notifications they will receive from USAMMDA.

Every serious adverse event that is not resolved at the time the initial written report is filed will have a follow-up report submitted when information is available. Any submitted report will be identified as “initial”, “follow-up”, or “medical monitor”.
The initial notification will include:

- The study protocol number and the name of the PI
- The participant study number, sex and age
- The date of onset of the SAE, and date of administration of study vaccine(s)

The PI will not wait to collect additional information to fully document the event before making notification of a serious adverse event. The telephone/e-mail report will be followed by a full written report using the SAE form within the CRF, detailing relevant aspects of the adverse events in question.

Instances of death, cancer or congenital abnormality in offspring of a study participant if brought to the attention of the investigator AT ANY TIME after cessation of the study AND suspected by the Investigators to be related to study medication will be reported to the sponsors and GSK Biologicals.

HSRRB, USAMRMC Deputy for Regulatory Compliance and Quality, Human Subjects Protection Division, U.S. Army Medical Research and Material Command, Fort Detrick, MD. Tel: DSN 343 7803 or 301-619-2165/6; Fax: DSN 343 7803 or 301-619-7803

DMID, Holli Hamilton, M.D., M.P.H., Tel: 301-402-8339, Fax: 301-435-3649

DSMB to be determined

SMC to be determined

Local Medical Monitor: Prof. Hamar A Traoré, HPG, BP 1805, Bamako, Tel: +223 222 5002

NIAID DSMB, Marilyn Powers, Tel: 301-846-7016, Fax: 301-846-7514

University of Mali, FMPOS Ethics Review Committee, Me A. Sylla, BP 1805, Bamako, Mali, Tel: + 223 222 52 77; Fax: + 223 222 81 09

University of Maryland IRB by E-mail to ORS@umaryland.edu

NIAID IRB, Dr. Peter Mannon (Tel: 301-435-9273, Fax: 301-435-6739)

GSK-Biologicals, Primary Contact: Amanda Leach, M.S.c, MRCPCH, Clinical Development Manager, GSK Biologicals, Tel: +32-2-656-7788, Fax: +32-2-656-6160, E-Mail: amanda.leach@gskbio.com. Back-up contact: Dr Marc Ceuppens, Manager Clinical Safety Vaccines, Tel: +32 2 656 8798, Fax: +32 2 656 8009, Mobile phone for 7/7 day availability: 32 477 404 713, E-Mail: marc.ceuppens@gskbio.com

10.6 Pregnancy

Participants who become pregnant during the study period (up to 30 days after receiving the last vaccine dose) must not receive additional doses of vaccine but may continue other study procedures.

Female participants will be instructed to notify the investigators if they become pregnant at any time during the 12-month study period. Although not considered an adverse event pregnancy will be reported in the same way as an adverse event. All pregnancies occurring during the study period will be followed to term, any premature termination reported, and the health status of the mother and child including date of delivery and the child's gender and weight will be reported to HSRRB with copy to DMID and GSK.

10.7 Treatment of adverse events

Treatment of any adverse event will be provided by the investigators with advice from the Local Medical Monitor. The applied measures will be recorded in the CRF of the participant. The recording of adverse events is an important aspect of study documentation. It is the responsibility of the investigators to document all adverse events according to the detailed guidelines set out. The participants will be instructed to contact the investigators immediately if they manifest any signs or symptoms they perceive as serious.

11 PARTICIPANT COMPLETION AND DROP-OUT

11.1 Definition

From the perspective of data analysis a 'drop-out' is any participant who did not come back for the concluding visit foreseen in the protocol. A participant who returns for the concluding visit foreseen in the protocol is considered to have completed the study.

11.2 Procedure for handling drop-outs

Investigators will attempt to contact those participants who missed scheduled follow-up visits. Information gathered will be described on the Study conclusion page of the CRF and on Medication/Adverse event forms as appropriate.

11.3 Reasons for drop-out

The Study conclusion page on the CRF will specify which of the following possible reasons were responsible for dropout of the participant from the study:

- Serious adverse event
- Non-serious adverse event
- Protocol violation (to be specified)
- Withdrawal of study consent, not due to an adverse event
- Migration from the study area
- Lost to follow-up
- Other (to be specified)

12 DATA MANAGEMENT AND ANALYSIS

Data entry will be performed on site and in the MRTC/MMVDU data management unit in Bamako if necessary. Data analysis and reporting for primary and secondary endpoints will be done by Statistics Collaborative, Inc., Washington, DC. Data entry and management systems will be 21 CFR Part 11 compliant.

12.1 Primary Endpoints

Occurrence of solicited symptoms during an 8-day follow-up period after vaccination (day of vaccination and days 1, 2, 3 and 7 after vaccination).

Occurrence of unsolicited symptoms during a 31-day follow-up period after vaccination (day of vaccination and 30 subsequent days).

Occurrence of serious adverse events during the study period.

12.2 Secondary Endpoints

Anti-MSP1 antibody titers at time points at which blood samples are collected for serology.

12.3 Study cohorts/datasets to be evaluated

12.3.1 Total cohort

The 'Total Cohort' will include all participants enrolled (defined as randomized to study groups) in the study.

12.3.2 Safety cohort

The 'Safety Cohort' will consist of all participants who have received at least one dose of study vaccine or comparator and for whom any data on safety are available.

The presentation of safety data will explore separately the adverse experiences among participants who received all vaccination, among those who received only some and among those with clinical violations of study protocol.

12.3.3 Immunogenicity cohort

The 'Immunogenicity Cohort' will include all evaluable participants (i.e., those meeting all eligibility criteria, complying with the procedures defined in the protocol, with no elimination criteria during the study) for whom data concerning immunogenicity endpoint measures are available. This will include participants for whom assay results are available for antibodies against at least one study vaccine antigen component after vaccination.

12.4 Estimated sample size

This Phase I trial is not powered to detect differences between groups. Even if comparative statistics for the safety variables will be computed, the study will have low power to detect anything other than very large differences in the incidence of local and general side effects between the vaccination groups. This is done weighing the need to detect any possible untoward reactions against the need to limit the number of volunteers involved for safety purposes. The sample size of 40 is widely accepted and used in industry for the initial assessment of the safety, tolerance and immunogenicity of an investigational vaccine. Incorporation of a comparator vaccine as control will enable broad initial estimates of the incidence of local and general side effects and of immune responses among vaccine recipients.

Table 4. Event detection probability table.

Event Rate	Pr 0/20	Pr 1+/20
0.01	0.82	0.18
0.05	0.36	0.64
0.08	0.19	0.81
0.10	0.12	0.88

Event rate. True rate at which an event occurs.
Pr 0/20. Given the event rate, probability that no events will be detected among 20 vaccinees.

Pr 1+/20. Given the event rate, probability that one or more events will be detected among 20 vaccinees.

If the SAE rate is 8%, then the probability of observing at least one event in a group size of 20 (Pr 1+/20) is 0.81. If the SAE rate is 8%, then the probability of observing no event in a group size of 20 (Pr 0/20) is 0.19.

12.5 Final analysis

12.5.1 Analysis of demographics

Demographic characteristics (age, sex and place of residence) of each study cohort will be tabulated. The mean age (plus range and standard deviation) by sex of the enrolled participants, as a whole and per group will be tabulated.

12.5.2 Analysis of immunogenicity

Immunogenicity will be assessed in several ways. A series of graphs will display immunologic responses. For each vaccine group and timepoint, the distribution of anti-MSP-1 antibody levels and reverse cumulative curves will be plotted. Corresponding summary statistics will show means and standard deviations as well as median, 25th and 75th percentiles, and 10th and 90th percentiles. The statistics will be presented both as raw data and as log-transformed data.

In addition, for each treatment group and timepoint, anti-MSP-1 antibody levels will be presented as geometric means of OD units with 95% confidence intervals. For each vaccine group and for each timepoint, a table will show the proportion of volunteers with two-fold, four-fold, and eight-fold increases in anti-MSP-1 antibody titers. To describe more fully the antibody levels over time and to allow more precise estimates of relevant parameters, mixed models will be fit to the observations for individual volunteers and averaged over all volunteers in each vaccine group.

12.5.3 Analysis of safety

The overall percentage of participants with at least one local adverse event (solicited and unsolicited) and the percentage with at least one general adverse event (solicited and unsolicited) during the 8-day follow-up period after vaccination will be tabulated. The incidence, intensity and relationship of individual solicited symptoms over the 8-day follow-up period will be calculated per group and vaccine dose.

The number of participants with at least one report of an unsolicited adverse event, classified by WHO-preferred terms, reported up to 30 days after vaccination will be tabulated per group and vaccine dose. The intensity and relationship to vaccination of the unsolicited symptoms reported will also be assessed.

Serious adverse events are expected to be rare, but where observed will be described. Comparisons of incidence of symptoms, local and general symptoms will be made based on a two-tailed Fischer exact test. Analysis of safety during the 12-month follow-up period will consist of comparison of incidence of serious adverse events as well as hemoglobin, creatinine and ALT levels.

Clinical laboratory parameters

Hematological (CBC) and biochemical (ALT, creatinine) laboratory parameters will be measured at specific time points, Days 0, 14, 30, 44, 60, 74, 90 and starting on day 180 every 3 months. Clinically relevant abnormal values will be tabulated and a trend analysis could be performed if deemed necessary.

12.6 Preliminary analysis

Safety, reactogenicity and immunogenicity data after three vaccine doses will be compiled by Statistics Collaborative Inc., Washington D.C. based on CRF data transcribed on site. Analysis of safety will be done for each group without revealing the assignment of individual participants.

12.7 Administrative matters

To comply with Good Clinical Practices important administrative obligations relating to investigator responsibilities, monitoring, archiving data, audits, confidentiality and publications must be fulfilled. See Appendices 16.4 and 16.5 for details.

13 TIME FRAME

Screening: June 2003
Dose 1: June 2003
Dose 2: July 2003
Dose 3: August 2003
Start Post-immunization Surveillance Period: October 2003
Preliminary Report: March 2003
Final Report: October 2004

14 ETHICAL CONSIDERATIONS

14.1 Statement of Compliance and Ethical Reviews

The study is under FDA IND BB-9202 and will be conducted according to current Good Clinical Practices, US 21 CFR Part 50-Protection of Human Subjects and Part 56-Institutional Review Boards, U.S. Army Regulation AR 40-38 and AR 70-25, and the applicable rules and regulations of Mali.

The FMPOS Ethical Review Committee will review and approve the protocol prior to study start. In addition to the review by the Human Subjects Research Review Board (HSRRB) of the Office of the Surgeon General, US Army, the study will be reviewed and approved by DMID and the NIAID and University of Maryland IRBs. Documentation of the approval by these ethical review boards will be conserved in the PI’s study file.

14.1.1 Institutional Review Boards (IRB)

All amendments will be submitted to the FMPOS IRB (FWA00001769), the NIAID and UMB IRBs, the HSSRB and DMID. No amendments will go into effect without written approval from the FMPOS IRB, the NIAID IRB, the UMD IRB, HSRRB and DMID except when necessary to eliminate immediate hazards to the participants. Protocol deviations will also be reported to all the local IRBs and the HSRRB according to each IRB's policy. Also with regard to protocol violations, data will be entered and checked for missing or out-of-range or other inaccurate information. Source documents will be examined to determine whether missing data were not transcribed, unavailable or missing for unknown reasons and this information will be coded and documented in the database.

The investigators will inform all the IRBs and DMID of the following:

- All subsequent protocol amendments, informed consent changes or revisions of other documents originally submitted for review
- Serious and/or unexpected adverse events occurring during the study, where required
- New information that may affect adversely the safety of the participants or the conduct of the study
- An annual update and/or request for re-approval, where required
- When the study has been completed, where required.

14.2 Informed consent

The principles of informed consent in the current edition of the Declaration of Helsinki will be implemented in each clinical study before any protocol-specified procedures or interventions are carried out.

Information will be given in both oral and written form whenever possible and deemed appropriate by the IRB.

The written consent documents will embody the elements of informed consent as described in the current edition of the Declaration of Helsinki, will adhere to the ICH Harmonized Tripartite Guideline for Good Clinical Practice and will also comply with applicable Malian regulations.

14.3 Role of Local Medical Monitor (Local Safety Monitor)

Prof. Hamar Traoré is the Local Medical Monitor for this study. The term Local Medical Monitor is equivalent to the ICH term "Sponsor's Medical Expert". Prof Traoré's *curriculum vitae* will be maintained on record. He is a qualified and experienced physician not otherwise associated with this protocol, who is able to provide medical care to research subjects for conditions that may arise during the conduct of the study, and who will monitor the subjects during the conduct of the study. The medical monitor is required to review all serious adverse events (per ICH definitions) associated with the protocol and provide an unbiased written report of the event within 10 calendar days of the initial report. At a minimum, the local medical monitor should comment on the outcomes of the serious adverse event (SAE) and relationship of the SAE to the test product. The medical monitor should also indicate whether he concurs with the details of the report provided by the study investigator.

The Local Medical Monitor will support the clinical investigators and act as a link between the investigators and the DSMB.

The PI will report all serious adverse events to the Local Medical Monitor. He will review all serious adverse events associated with the protocol and will provide an unbiased written report of the event within 10 calendar days of the initial report. At a minimum, this report will comment on the outcomes of the adverse event and relationship to vaccination and indicate if the Local Medical Monitor concurs with the details of the report provided by the investigators.

- The involvement of the Local Medical Monitor will be particularly important when decisions related to safety of participants have to be made quickly. Code break envelopes will be in his safekeeping and he may unblind individual study participants if deemed necessary for medical and/or ethical reasons. In exceptional circumstances, for example a death possibly related to vaccination, he would have the authority to suspend the whole or any specific aspect of the trial pending review by the SMC and DSMB.

The Medical Monitor may recommence the trial after discussions the DSMB and sponsors, if the DSMB recommends resuming the trial and the sponsors agree. Notification will then be made to all IRBs.

Prof. Traoré will be on-site during active phases of immunization and during the immediate post-vaccination follow-up period. Dr. Issa Ben Zacour, a medical staff member at the Bandiagara CSREF, will act as the on-site local Medical Monitor in support of Prof. Traoré between the vaccinations. Dr. Ben Zacour is residing in Bandiagara full time, but is occasionally required to travel out of Bandiagara for professional duties, during which time, in consultation with Prof. Traoré and the senior study investigators, he will designate another qualified physician on staff at CSREF and familiar with the study to cover his responsibilities.

The Local Medical Monitor's role will include:

- Acting as the study volunteers' advocate
- Promptly communicating relevant safety information to the SMC and DSMB
- Providing advice to the investigators on whether a set of clinical circumstances in a study warrants formal notification to the USAMMDA, the SMC and DSMB.
- Providing clinical advice on any illness in study subjects especially in circumstances in which treatment might influence the course of the trial.
- Review all SAEs as outlined above

The Local Medical Monitor will liaise closely with the PI throughout the course of the trial and relay relevant safety information to the PI, the SMC and the DSMB. The PI will inform the FMPOS IRB, the SMC and USAMMDA.

14.4 Safety Monitoring Committee (SMC)

An independent Safety Monitoring Committee will be constituted to help the Medical Monitor review safety data in real time. This committee will consist of the Local Medical Monitor and at least 2 other independent experts. The role of the SMC will be to review safety data between immunizations and to approve progression to the next immunization,

whereas the role of the DSMB will be to assess and evaluate the accumulated safety and immunogenicity data from this trial and preceding trials, in order to make global decisions regarding the future of this vaccine formulation; the DSMB will not be required to meet during the conduct of this study. The Safety Monitoring Committee will hold two regularly scheduled conference calls, to review the safety data generated from the trial up to that point: the first will occur three weeks after the 1st immunization (i.e., one week prior to the 2nd immunization) and the second will occur three weeks after the 2nd immunization (i.e., one week prior to the 3rd immunization). The purpose of these conference calls will be to review the accumulated safety data in order to determine whether or not the study should proceed to the next immunization. The study will not proceed to the next immunization unless explicitly agreed to by the members of the Safety Monitoring Committee, either in the form of a letter or email from the Local Medical Monitor. Other conference calls and/or meetings may be required.

The investigator will inform the SMC of:

- All subsequent protocol amendments, informed screening or study consent form changes or revisions of other documents originally submitted for review
- Serious adverse events (SAEs) and grade 3 adverse experiences (as defined in Table 3, section 10.2) occurring during the study, regardless of relationship to the study vaccine
- New information that may affect adversely the safety of the subjects or the conduct of the study.

The SMC will be empowered to put the study on hold pending review of potential safety issues. The SMC would request additional information from the Principal Investigator as needed and will perform any appropriate statistical calculations to support discussions with the DSMB. Final recommendations to permanently terminate or restart the study is made by the DSMB to the sponsors. All documentation provided to members of the SMC for information and review must be treated in a confidential manner.

14.5 Data Safety Monitoring Board (DSMB)

14.5.1 Composition of the DSMB

An independent committee consisting of up to five experts in malaria, infectious diseases, biostatistics and other appropriate disciplines was formed to oversee ethical and safety aspects of earlier studies of this product.

14.5.2 Role of the DSMB

The DSMB may convene during the study proper to review any relevant safety data and to review and approve the Report and Analysis Plan (RAP), and at the close out of the study. Other unscheduled meetings may be required. Meetings must be documented and minutes made available for the study files on site and to the sponsors. The DSMB may, if deemed necessary, convene a meeting with or request further information from the Principal Investigators, the Local Medical Monitor, the Safety Monitoring Committee, the WRAIR, the DMID, U-Maryland and GSK Biologicals' designated project representatives at any stage of the study. The SMC and local medical monitors will be responsible for real-time safety

monitoring and will assess adverse events between each set of immunizations as described in Sections 14.3 and 14.4. However, both the SMC and the DSMB will receive SAE reports and both will be independently empowered to put the trial on hold. If the trial is put on hold, the DSMB will analyze data and reports and recommend to the sponsors whether to continue the trial or to stop it. The DSMB is the same DSMB that has been monitoring this vaccine for all of its clinical trials, and will provide general oversight and receive a final report of the study, but will not be primarily responsible for real-time safety monitoring. In short, the DSMB is constituted for review of the product used in the clinical trial. With their broad experience with this product and awareness of other ongoing and planned trials, they will benefit from this level of involvement with our study and will be able to notify us if important adverse events occur in other studies.

The investigator must inform the DSMB of:

- All subsequent protocol amendments, informed screening or study consent form changes or revisions of other documents originally submitted for review
- All serious adverse events (SAEs) and grade 3 adverse experiences (as defined in Table 3, section 10.2), including death, occurring during the study, regardless of relationship to the study vaccine
- All subsequent protocol administrative changes (for information)
- New information that may affect adversely the safety of the subjects or the conduct of the study.

The DSMB will be empowered to put the study on hold pending review of potential safety issues. The DSMB will review for data trends in relation to safety issues and will have the right to request additional clinical data about all cases from the Principal Investigator as needed. The DSMB will perform any appropriate statistical calculations to support recommendations to the sponsors. All documentation provided to members of the DSMB for information and review must be treated in a confidential manner.

The Chairman of the DSMB will be invited to propose new members for the board in the event that members must be replaced.

14.6 NIAID DSMB

Because this is a randomized and blinded study, NIAID policy mandates that it be reviewed by the permanent NIAID DSMB. (**NOTE: Throughout the protocol, unless "NIAID DSMB" is specified, the term "DSMB" will refer to the primary DSMB described above that has overseen all trials of this vaccine.)** This DSMB has been constituted to review the data and analysis plans of all intramural NIAID clinical studies that require DSMB oversight, and consists of experts in infectious diseases, biostatistics, and clinical trials. It serves in an advisory capacity to the NIAID IRB, which can either accept or reject it's recommendations. The Board meets at regular periods during the year, but may convene in between their regularly scheduled meetings should the need arise.

This protocol and the Report and Analysis Plan (RAP) will be submitted to the NIAID DSMB for their review; however, the Board's review is not required before the start of the study. Additionally, all cumulative safety data reports from the trial will be submitted to the Board at the same time that they are submitted to the SMC (i.e., one week before the 2nd vaccination, and one week before the 3rd vaccination). After the third and final vaccination, additional safety and immunologic results and reports will be submitted to the NIAID DSMB

as they become available. A final report will be submitted to the NIAID DSMB following completion of the study.

Safety data from the study that is submitted to the NIAID DSMB in between vaccinations may not be reviewed by the DSMB before subsequent vaccinations are administered, due to the Board's meeting schedule. It is the responsibility of the Local Medical Monitor and SMC to review this data in "real time". As stated above, the role of the NIAID DSMB is advisory: it does not, therefore, have the authority to place the study on hold. This responsibility rests foremost with the Local Medical Monitor and SMC, who will be actively assessing the safety information throughout the trial. The NIAID DSMB will have access to the randomization code if requested, as they may wish to review the data in an unblinded fashion prior to breaking the randomization code.

It is the Principal Investigator's responsibility to ensure that the NIAID DSMB reviews the current protocol at it's meetings. Occurrence of SAE's will be reported to the NIAID DSMB at the same time that they are reported to the IRB's, FMP1 DSMB, and SMC. Additionally, any new information that may affect adversely the safety of the subjects or the conduct of the study will be submitted to the NIAID as it becomes available.

14.7 Risks and Potential Benefits to the Participants

14.7.1 Vaccination

Risks associated with both vaccinations include local inflammatory reactions to the injected product, such as injection site pain and swelling and some limitation of arm movement. Systemic effects may include flu-like syndrome, fever, chills, nausea/GI symptoms, headache, malaise, myalgia and arthralgia. While rare, allergic reactions, including life-threatening anaphylaxis, are associated with many vaccine preparations and must therefore be considered as a potential risk in this study. Risks associated with drawing blood include fainting, infection and bruising.

14.7.2 Medical treatment for participants

Free medical treatment will be provided to all enrolled participants during the active immunization phase and the follow-up period. The pharmacy at the BMP clinic will have sufficient provisions to provide participants with drugs for the treatment of minor illnesses free of charge. If further evaluation or treatment is necessary the participant will be referred to the regional hospital in Mopti located 75 km from Bandiagara. If the Investigators judge that a participant requires hospitalization in Mopti, or at the National Hospital in Bamako, referral and transportation to these places will be arranged and the medical management of the participants will be monitored by senior physician investigators and the local medical monitor.

Medical care for ailments not related to vaccination will not extend beyond the study period. Medical care for ailments related to vaccination will extend at least until the condition has resolved.

14.7.3 Rabies vaccination

During the conduct of the study participants randomized to receive rabies immunization will benefit from this due to the assumed prevalence of rabies in Bandiagara. At the end of the study all participants will be informed of the vaccine they received. Volunteers randomized to the FMP1 vaccine will be offered rabies immunization at that time. This will be done at the recommended schedule of 0, 7 and 21 days.

14.7.4 Risks associated with malaria treatment

The medications that will be used for routine treatment of clinical malaria episodes – chloroquine, sulfadoxine-pyrimethamine, and quinine – are all FDA-approved medications for the treatment of malaria and are recommended for the treatment of malaria by the Malian Ministry of Health and the World Health Organization. They have good safety profiles, although all have well-known side effect profiles.

Chloroquine most commonly is associated with benign itching, and very rarely with nausea, vomiting, dizziness, headache, blurred vision, fatigue, diarrhea and convulsions. Retinopathy is rarely associated with chronic use for rheumatoid arthritis, but not with periodic treatment for malaria.

No significant toxic effects have been reported with pyrmimethamine when used for malaria treatment. Sulfonamides have been associated with severe cutaneous reactions, but these occur when used chronically for prophylaxis, not for periodic treatment. Sulfonamides can also precipitate hemolytic crisis in persons with the Mediterranean form of G6PD deficiency but this is virtually unheard of in persons of subSaharan African origin whose form of G6PD deficiency is less severe. Sulfonamides have also been associated with kernicterus and are relatively contraindicated in late pregnancy.

Quinine can cause a temporary syndrome called cinchonism (tinnitus, heacache, nausea, dizziness, tremors). Rapid infusion and prolonged use of parenteral quinine can cause hypoglycemia. Serious, idiosyncratic effects are very rare and include angioedema and agranulocytosis.

14.7.5 Pregnancy

The effects of both the study and comparator vaccines on the unborn fetus are unknown. Female participants will be counseled to avoid becoming pregnant during the immunization phase of the study and up to one month after the last immunization. Any female participant interested in contraceptive methods will be referred to the local health center family planning services for evaluation and institution of an appropriate contraceptive method.

14.7.6 Benefits

Participants may not receive any direct benefit from the experimental vaccine. However, they will receive follow-up medical care during the 12 months of the study, at the BMP Clinic in Bandiagara. Treatment for malaria and for other illnesses will be free of charge, according to the standard of care that is available in Mali. They will still be able to receive free medical care at the clinic even if they are withdrawn from the study. At the end of the study, participants will be told what vaccine they received. If they received the malaria vaccine, they may come to the clinic after the study to get the rabies vaccine, if they wish, so that all participants may potentially benefit from immunization against rabies.

14.8 Precautions to minimize risks

14.8.1 Vaccination

As outlined above, the participants will be monitored closely during their participation in this study. The study vaccine has been prepared according to Good Manufacturing Procedures (GMP). The vaccine will be administered by experienced investigators with drugs and equipment available for the treatment of anaphylaxis. All vaccine doses will be given by intramuscular injection to minimize injection site reactions like pain.

14.8.2 Malaria treatment

Medications available for the treatment of clinical malaria include chloroquine at 25 mg/kg over 3 days (day 1 and day 2, 10 mg/kg and 5 mg/kg on day 3). In case of intolerance to chloroquine or lack of efficacy, sulfadoxine-pyrimethamine will be used at 1 tablet per 20 kg, given as a single dose (3 tablets for adults and persons weighing ≥60kg). Severe episodes of clinical malaria will be treated with quinine. The treatment of clinical malaria will be done according to National Malaria Control Program recommendations and based on the investigators' SOPs.

14.8.3 Protection of study staff

All study personnel have been trained to follow Universal Precautions. Additionally, the following approved SOPs from the BMP clinical lab elaborate the precautions that will be taken by study personnel to minimize risks: General Laboratory Safety, Exposure to Blood and Infectious Material, and Waste Management.

14.9 Procedures for Maintaining Confidentiality

Participants will be assigned a unique identifier number. All results will be referred to this number. Study records will only be available to staff members and will be kept locked at the study site and will conform to the investigators' SOPs. Following the conclusion of the study, records will be maintained on site for a minimum of two years, after which they will be stored long-term in the MMVDU data storage facilities in Bamako. They will remain available for future audits. Representatives of the US Army Medical Research and Material Command (USAMRMC), the FDA and the sponsors may review these records.

14.10 Compensation

Each participant will be compensated for the time they donate to the study by being given 50 kg of rice and 50 kg of millet, total value about $40. One half will be provided after the first immunization and one half at the end of the study. Throughout Mali, the availability of food is subject to seasonal variation in relation to the harvest season. However, there is no recent history of famine or starvation. In the region of our study site, while cases of pediatric malnutrition are occasionally seen at the Health Centre, these are attributable to poor feeding habits rather than to scarcity of food, and the treatment is educating the parents to provide more nutritional foods to small children. The total amount of food to be distributed in two parts over the course of one year will last an average family approximately four weeks. The type of food distributed, rice and millet, are staple starches that are typically served

accompanied by a sauce containing some sort of meat as well as vegetables, and therefore are only a part of the local diet. This amount of compensation is consistent with what we have provided to participants of longitudinal studies in Mali for several years, and has been carefully considered by the local Malian IRB, who have determined that it is appropriate compensation for time lost to study procedures and not coercive.

14.11 Financing and insurance

This study will be financed primarily by contract N01-AI-85346 from the DMID, National Institutes of Health, to the University of Maryland with a subcontract to the University of Mali. Additional resources are provided by the intramural MVDU, National Institutes of Health. These additional resources are primarily in the form of infrastructure including vehicles, communications, computer networks, as well as the training, preparation and equipping of the clinical laboratory.

GSK Biologicals has and will maintain during the term of its Material Transfer Agreement with the University of Bamako or the Protocol, whichever is the longer, a clinical trial liability insurance policy sufficient to covert he cost of reasonable medical care required to treat or stabilize adverse reactions suffered by patients who received FMP1 adjuvanted with AS02A in accordance with the approved Protocol, to the extent the medical care is not covered by the patients' medical or hospital insurance or by third party or governmental programs providing such coverage.

15 REFERENCES

1. Miller,L.H., Baruch,D.I., Marsh,K. & Doumbo,O.K. The pathogenic basis of malaria. *Nature* **415**, 673-679 (2002).

2. Chitnis,C.E. Molecular insights into receptors used by malaria parasites for erythrocyte invasion. *Curr. Opin. Hematol.* **8**, 85-91 (2001).

3. Dvorak,J.A., Miller,L.H., Whitehouse,W.C. & Shiroishi,T. Invasion of erythrocytes by malaria merozoites. *Science* **187**, 748-750 (1975).

4. Aikawa,M., Miller,L.H., Johnson,J. & Rabbege,J. Erythrocyte entry by malarial parasites. A moving junction between erythrocyte and parasite. *J. Cell Biol.* **77**, 72-82 (1978).

5. Snow,R.W., Craig,M., Deichmann,U. & Marsh,K. Estimating mortality, morbidity and disability due to malaria among Africa's non-pregnant population. *Bull. World Health Organ* **77**, 624-640 (1999).

6. Alonso,P.L., Armstrong,J.R. & Lindsay,S.W. Malaria, bednets, and mortality. *Lancet* **338**, 897 (1991).

7. D'Alessandro,U. *et al.* Mortality and morbidity from malaria in Gambian children after introduction of an impregnated bednet programme. *Lancet* **345**, 479-483 (1995).

8. Nevill,C.G. *et al.* Insecticide-treated bednets reduce mortality and severe morbidity from malaria among children on the Kenyan coast. *Trop. Med. Int. Health* **1**, 139-146 (1996).

9. Brooker,S. *et al.* Situation analysis of malaria in school-aged children in Kenya - what can be done? *Parasitol. Today* **16**, 183-186 (2000).

10. Sachs,J. & Malaney,P. The economic and social burden of malaria. *Nature* **415**, 680-685 (2002).

11. Coulibaly,D. *et al.* Impact of preseason treatment on incidence of falciparum malaria and parasite density at a site for testing malaria vaccines in Bandiagara, Mali. *Am. J. Trop. Med. Hyg.* **67**, 604-610 (2002).

12. Diallo,D. *et al.* Haemoglobinopathies C and S in the Dogons. *Nouvelle Revue Francaise d Hematologie* **35**, 551-554 (1994).

13. Agarwal,A. *et al.* Hemoglobin C associated with protection from severe malaria in the dogon of mali, a west african population with a low prevalence of hemoglobin S [In Process Citation]. *Blood* **96**, 2358-2363 (2000).

14. Djimde,A. *et al.* A molecular marker for chloroquine-resistant falciparum malaria. *N. Engl. J. Med.* **344**, 257-263 (2001).

15. Djimde,A., Doumbo,O.K., Steketee,R.W. & Plowe,C.V. Application of a molecular marker for surveillance of chloroquine- resistant falciparum malaria. *Lancet* **358**, 890-891 (2001).

16. Snow,R.W. *et al.* Severe childhood malaria in two areas of markedly different falciparum transmission in east Africa. *Acta Tropica* **57**, 289-300 (1994).

17. Dame,J.B. *et al.* Structure of the gene encoding the immunodominant surface antigen on the sporozoite of the human malaria parasite Plasmodium falciparum. *Science* **225**, 593-599 (1984).

18. Cerami,C. *et al.* The basolateral domain of the hepatocyte plasma membrane bears receptors for the circumsporozoite protein of Plasmodium falciparum sporozoites. *Cell* **70**, 1021-1033 (1992).

19. Ballou,W.R. *et al.* Safety and efficacy of a recombinant DNA Plasmodium falciparum sporozoite vaccine. *Lancet* **1**, 1277-1281 (1987).

20. Herrington,D.A. *et al.* Safety and immunogenicity in man of a synthetic peptide malaria vaccine against Plasmodium falciparum sporozoites. *Nature* **328**, 257-259 (1987).

21. Fries,L.F. *et al.* Safety, immunogenicity, and efficacy of a Plasmodium falciparum vaccine comprising a circumsporozoite protein repeat region peptide conjugated to Pseudomonas aeruginosa toxin A. *Infect. Immun.* **60**, 1834-1839 (1992).

22. Ribi,E., Cantrell,J. & Feldner,T. Microbiology. Levine,L., Bonventre,P.F. & Morello,J.A. (eds.) (American Society of Microbiology, Washington, DC,1986).

23. Fries,L.F. *et al.* Liposomal malaria vaccine in humans: a safe and potent adjuvant strategy. *Proc. Natl. Acad. Sci. U. S. A* **89**, 358-362 (1992).

24. Hoffman,S.L. *et al.* Safety, immunogenicity, and efficacy of a malaria sporozoite vaccine administered with monophosphoryl lipid A, cell wall skeleton of mycobacteria, and squalane as adjuvant. *Am. J. Trop. Med. Hyg.* **51** , 603-612 (1994).

25. Vreden,S.G., Verhave,J.P., Oettinger,T., Sauerwein,R.W. & Meuwissen,J.H. Phase I clinical trial of a recombinant malaria vaccine consisting of the circumsporozoite repeat region of Plasmodium falciparum coupled to hepatitis B surface antigen. *Am. J. Trop. Med. Hyg.* **45**, 533-538 (1991).

26. Gordon,D.M. *et al.* Safety, immunogenicity, and efficacy of a recombinantly produced Plasmodium falciparum circumsporozoite protein-hepatitis B surface antigen subunit vaccine. *J. Infect. Dis.* **171**, 1576-1585 (1995).

27. Bomford,R. *et al.* Adjuvanticity and ISCOM formation by structurally diverse saponins. *Vaccine* **10**, 572-577 (1992).

28. Stoute,J.A. *et al.* A preliminary evaluation of a recombinant circumsporozoite protein vaccine against Plasmodium falciparum malaria. RTS,S Malaria Vaccine Evaluation Group. *N. Engl. J. Med.* **336**, 86-91 (1997).

29. Stoute,J.A. *et al.* Long-term efficacy and immune responses following immunization with the RTS,S malaria vaccine. *J. Infect. Dis.* **178**, 1139-1144 (1998).

30. Kester,K.E. *et al.* Efficacy of recombinant circumsporozoite protein vaccine regimens against experimental Plasmodium falciparum malaria. *J. Infect. Dis.* **183**, 640-647 (2001).

31. Bojang,K.A. *et al.* Efficacy of RTS,S/AS02 malaria vaccine against Plasmodium falciparum infection in semi-immune adult men in The Gambia: a randomised trial. *Lancet* **358**, 1927-1934 (2001).

32. Blackman,M.J., Heidrich,H.G., Donachie,S., McBride,J.S. & Holder,A.A. A single fragment of a malaria merozoite surface protein remains on the parasite during red cell invasion and is the target of invasion- inhibiting antibodies. *J. Exp. Med.* **172**, 379-382 (1990).

33. Chang,S.P., Gibson,H.L., Lee-Ng,C.T., Barr,P.J. & Hui,G.S. A carboxyl-terminal fragment of Plasmodium falciparum gp195 expressed by a recombinant baculovirus induces antibodies that completely inhibit parasite growth. *J. Immunol.* **149**, 548-555 (1992).

34. Egan,A.F. *et al.* Clinical immunity to Plasmodium falciparum malaria is associated with serum antibodies to the 19-kDa C-terminal fragment of the merozoite surface antigen, PfMSP-1. *J. Infect. Dis.* **173**, 765-769 (1996).

35. Chang,S.P. *et al.* A recombinant baculovirus 42-kilodalton C-terminal fragment of Plasmodium falciparum merozoite surface protein 1 protects Aotus monkeys against malaria. *Infect. Immun.* **64**, 253-261 (1996).

36. Ling,I.T., Ogun,S.A. & Holder,A.A. Immunization against malaria with a recombinant protein. *Parasite Immunol.* **16**, 63-67 (1994).

37. Daly,T.M. & Long,C.A. A recombinant 15-kilodalton carboxyl-terminal fragment of Plasmodium yoelii yoelii 17XL merozoite surface protein 1 induces a protective immune response in mice. *Infect. Immun.* **61**, 2462-2467 (1993).

38. Daly,T.M. & Long,C.A. Humoral response to a carboxyl-terminal region of the merozoite surface protein-1 plays a predominant role in controlling blood-stage infection in rodent malaria. *J. Immunol.* **155**, 236-243 (1995).

39. Burghaus,P.A. *et al.* Immunization of Aotus nancymai with recombinant C terminus of Plasmodium falciparum merozoite surface protein 1 in liposomes and alum adjuvant does not induce protection against a challenge infection. *Infect. Immun.* **64**, 3614-3619 (1996).

40. Crisanti,A. *et al.* Epitopes recognized by human T cells map within the conserved part of the GP190 of P. falciparum. *Science* **240**, 1324-1326 (1988).

41. Rzepczyk,C.M. *et al.* Analysis of human T cell response to two Plasmodium falciparum merozoite surface antigens. *Eur. J. Immunol.* **19**, 1797-1802 (1989).

42. Wu,J.Y. *et al.* Saponin adjuvant enhancement of antigen-specific immune responses to an experimental HIV-1 vaccine. *J. Immunol.* **148**, 1519-1525 (1992).

43. Soltysik,S., Bedore,D.A. & Kensil,C.R. Adjuvant activity of QS-21 isomers. *Ann. N. Y. Acad. Sci.* **690**, 392-395 (1993).

44. Myers,K.R., Truchot,A.T. & Ward,J. Cellular and Molecular Aspects of Endotoxin Reactions: Proceedings of the 1st Congress of the International Endotoxin Society. Nowotny,A. (ed.), pp. 145-156 Amsterdam,1990).

45. Loppnow,H., Ddurrbaum,I. & Brade,H. Advances in Experimental Medicine and Biology. Friedman,H., Klein,T.W. & Nakano,M. (eds.), pp. 561-566 (Plenum Press, New York,990).

46. Johnson,A.G. & Tomai,M.A. Advances in Experimental Medicine and Biology. Friedman,H., Klein,T.W. & Nakano,M. (eds.), pp. 567-579 (Plenum Press, New York,1990).

47. Stoute,J.A. *et al.* A preliminary evaluation of a recombinant circumsporozoite protein vaccine against Plasmodium falciparum malaria. RTS,S Malaria Vaccine Evaluation Group. *N. Engl. J. Med.* **336**, 86-91 (1997).

48. Open phase I study to evaluae the safety, reactogenicity and immunogenicity of SMilthKine Beecham Biologicals' vaccine against malaria. (Malaria-002). SmithKline Beecham Biologicals. 1997.

49. Open phase I study to evaluate the safety, reactogenicity and immunogenicity of SmithKine Beecham Biologicals' candidate vaccine against malaria in semi-immune adult males in the Gambia. Protocol 257049/004 (Malaria-004) - Interim safety report March 23, 1998. SmithKline Beecham Biologicals. 1998.

50. Maupas,P. *et al.* [Epidemiology and pathologic results of chronic carriers state of hepatitis B in Mali]. *Bull. Soc. Pathol. Exot. Filiales.* **74**, 722-732 (1981).

51. Sidibe,S., Sacko,B.Y. & Traore,I. [Prevalence of serologic markers of the hepatitis B virus in pregnant women of Bamako, Mali]. *Bull. Soc. Pathol. Exot.* **94**, 339-341 (2001).

52. Dreesen,D.W., Fishbein,D.B., Kemp,D.T. & Brown,J. Two-year comparative trial on the immunogenicity and adverse effects of purified chick embryo cell rabies vaccine for pre-exposure immunization. *Vaccine* **7**, 397-400 (1989).

53. Nicholson,K.G., Farrow,P.R., Bijok,U. & Barth,R. Pre-exposure studies with purified chick embryo cell culture rabies vaccine and human diploid cell vaccine: serological and clinical responses in man. *Vaccine* **5**, 208-210 (1987).

54. Vodopija,I. *et al.* An evaluation of second generation tissue culture rabies vaccines for use in man: a four-vaccine comparative immunogenicity study using a pre- exposure vaccination schedule and an abbreviated 2-1-1 postexposure schedule. *Vaccine* **4**, 245-248 (1986).

55. Wasi,C. *et al.* Purified chick embryo cell rabies vaccine. *Lancet* **1**, 40 (1986).

56. Cox,J.H. & Schneider,L.G. Prophylactic immunization of humans against rabies by intradermal inoculation of human diploid cell culture vaccine. *J. Clin. Microbiol.* **3** , 96-101 (1976).

57. Kuwert,E.K., Marcus,I., Werner,J., Iwand,A. & Thraenhart,O. Some experiences with human diploid cell strain-(HDCS) rabies vaccine in pre- and post-exposure vaccinated humans. *Dev. Biol. Stand.* **40**, 79-88 (1978).

58. Ajjan,N. *et al.* [Results of preventive rabies vaccination with a concentrated vaccine of the PM/WI38-1503-3M rabies strain cultured on human diploid cells. Preparation of mixed antirabies-antitetanus hyperimmune immunoglobulin by plasmapheresis of blood taken from vaccinated veterinary students]. *Dev. Biol. Stand.* **40**, 89-100 (1978).

59. Costy-Berger,F. [Preventive rabies vaccination using vaccine prepared from human diploid cells]. *Dev. Biol. Stand.* **40**, 101-104 (1978).

60. Recommendations of the Immunizations Practices Advisory Committee (ACIP). Rabies Prevention United States. 393-408. 1984. Atlalnta, Centers for Disease Control. Morbidity and Mortality Weekly Report.

61. Boe,E. & Nyland,H. Guillain-Barre syndrome after vaccination with human diploid cell rabies vaccine. *Scand. J. Infect. Dis.* **12**, 231-232 (1980).

62. Adverse reactions to human diploid cell rabies vaccine. 29, 609-610. 1980. Atlanta, Centers for Disease Control.

63. Bernard,K.W., Smith,P.W., Kader,F.J. & Moran,M.J. Neuroparalytic illness and human diploid cell rabies vaccine. *JAMA* **248**, 3136-3138 (1982).

64. Systemic allergic reactions following immunization with human diploid cell rabies vaccine. 33, 185-187. 1984. Atlanta, Centers for Disease Control. Morbidity and Mortality Weekly Report.

16 APPENDICES

16.1 Screening and study informed consent

The consent forms generated by the investigator (along with the protocol, and any other necessary documentation) must be approved and signed by the FMPOS Ethical Review Board (FWA #00001769).

Prior to initiating any study in Bandiagara, the senior Malian and U.S. investigators visit the local commandant (representative of the national government), the mayor, the director of the local school system, the chiefs of each of the eight quartiers of Bandiagara, the medical director of the local health center, the director of the Bandiagara Center for Research of Traditional Medicine, and the head of the Bandiagara traditional healers' association. These are courtesy visits in which results of the previous year's studies are summarized and plans for new studies are explained and any questions are answered. In accordance with the tradition in Mali, small quantities of kola nuts are given to the chiefs of the quartiers and the traditional healers as a sign of respect.

These individual meetings are followed by a larger community meeting attended by the above personages as well as numerous other local health care providers, traditional healers and notable citizens (including several respected women from the community). Planned studies are explained in more detail, and ample time is given for carefully and thoroughly addressing all questions and concerns. This question and answer period is frequently prolonged with many detailed and often sophisticated questions being raised. Each presentation, question and response is translated from French into Dogon and Peulh so that all present understand the entire discourse.

Once this group of community leaders has expressed their approval of the planned study, they disseminate information to their various constituencies, so that when potential recruits are approached by study staff they are already generally aware of the nature of the impending study. The investigators do not consider this process to constitute "community consent" in addition to or in lieu of individual informed consent, but rather a community "permission to enter" that is a necessary prerequisite to conducting any study in a tight-knit and highly organized traditional rural community such as Bandiagara.

Prior to initiating screening and informed consent, the study team meets to review the oral translation of the consent forms into the relevant local languages and dialects word by word, until there is consensus that the individuals responsible for giving consent in each language are conveying as accurately as possible the exact content of the IRB-approved French language consent form.

At the times of screening and recruitment, the Consent Forms are read to participants who speak French, and translated orally into the language of choice of each participant. In all cases, the investigator will give the participants ample opportunity to inquire about the details of the study and to ask any questions before dating and signing the consent forms. All illiterate individuals will have the study and consent forms explained to them point by point by the interviewer in the presence of a witness who will sign the consent form. Witnesses will have no association with the conduct of the study and will not be related to the study subject.

Informed consent will be documented by the use of a written consent form approved by the IRBs and signed or thumbprinted and dated by the participant, and by the person who conducted the informed consent discussion. Thumbprinting will be used for illiterate persons, who are expected to constitute the majority of participants. The consent will be orally

translated into native languages from the French written version of the consent form. A witness will assist during the procedure. After the participant clearly states that she/he has understood what was explained and agrees to participate to the study, the consent forms will be filled. The participant will be asked if she/he prefer to thumbprint or to sign. In the case of the thumbprint option, the distal end of her/his left thumb will be applied to a stamp inker and then firmly applied to the space on the consent forms reserved for thumbprints. This procedure has been followed for many years by the BMP team, and thumbprints are uniformly legible.

The signature/thumbprint confirms that the consent is based on information that has been understood. Each participant's signed informed consent form must be kept on file by the investigator for possible inspection by regulatory authorities. The subject will receive a copy of the signed and dated written informed consent forms and any other written information provided by the investigator, and will receive copies of any signed and dated consent form updates and any amendments to the written information.

The consent forms will reflect the realities of the study site and may therefore differ from standard consent forms for U.S. sites in some details. For example, the vast majority of study participants do not use telephones, fax or mail, and so contact information is provided in terms of local physicians who can be visited directly and who can themselves reach the investigators directly or by telephone or fax.

16.2 Screening recruitment radio announcement text

"The Bandiagara Research Project team from the Faculty of Medicine in Bamako has returned to Bandiagara, and sends its greetings to the population of Bandiagara. The team is here to test an experimental malaria vaccine, to see if it is safe to use in adults who live in a place where they get malaria. Adult men and women aged 18-55 years who live in Bandiagara town and are interested in participating in this research study are invited to come to the Bandiagara Health Center at [*time*] on [*date*] to learn more about this study.**"**

16.3 USAMMDA reporting scheme of SAEs

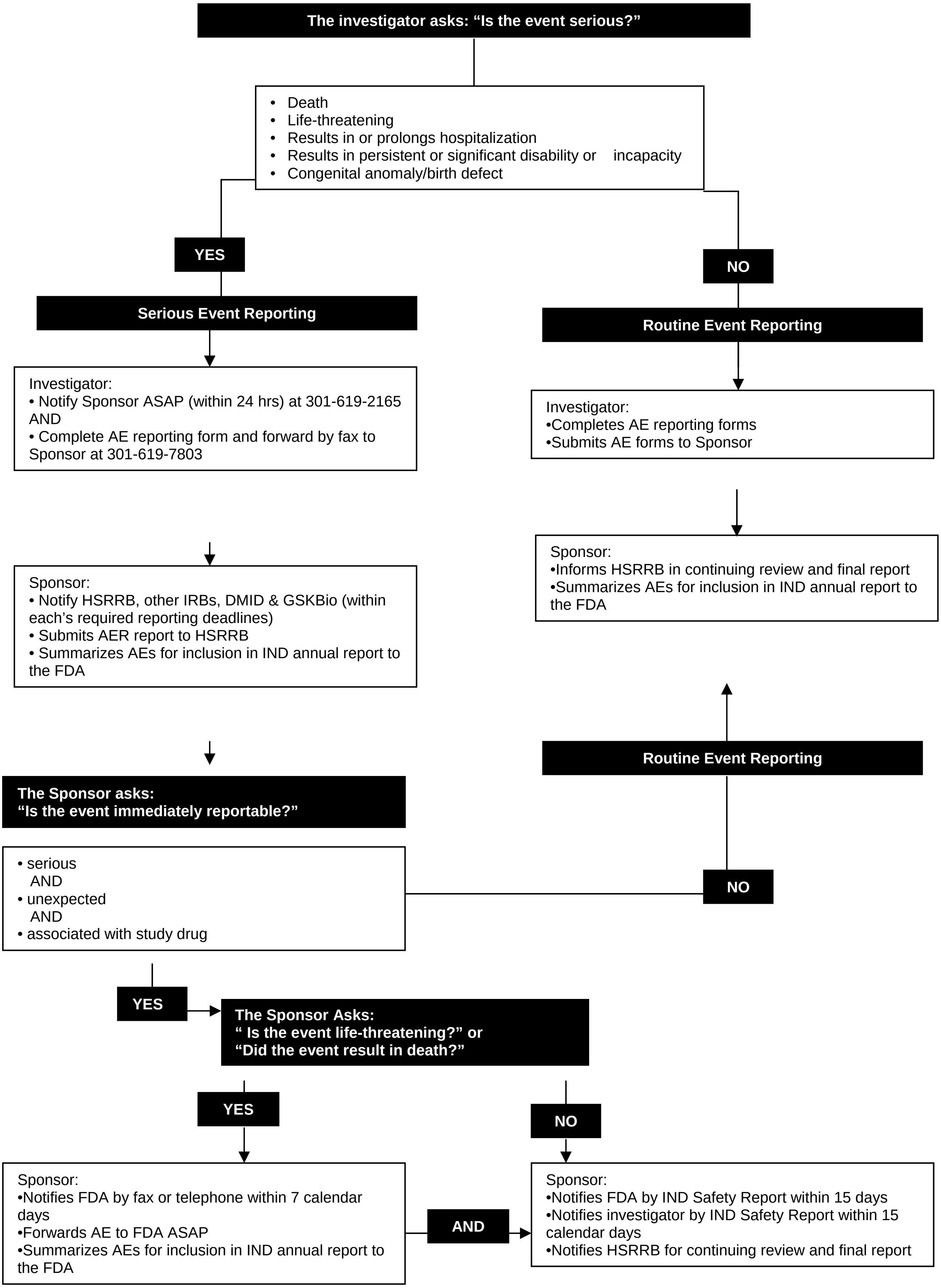

16.4 Administrative Matters

I. RESPONSIBILITIES OF THE INVESTIGATOR

To ensure that he/she has sufficient time to conduct and complete the study and has adequate staff and appropriate facilities which are available for the duration of the study and to ensure that other studies do not divert essential subjects or facilities away from the study at hand.

To submit an up-to-date curriculum vitae and other credentials (e.g. medical license number in the United States) to the sponsor and-where required-to relevant authorities.

To acquire the normal ranges for laboratory tests performed locally and, if required by local regulations, obtain the Laboratory License or Certification.

*To prepare and maintain adequate case histories designed to record observations and other data pertinent to the study.

To conduct the study in compliance with the protocol and appendices.

To cooperate with representatives of the study sponsor and IND sponsor in the monitoring process of the study and in resolution of queries about the data.

II. PROTOCOL AMENDMENTS AND MODIFICATIONS

No changes to the study protocol will be allowed unless discussed in detail with the sponsors and GSK Biologicals and filed as an amendment/modification to this protocol. Any amendment /modification to the protocol will be adhered to by the participating center and will apply to all subjects. Written IRB approval of protocol amendments is required prior to implementation. All amendments will be submitted to the HSRRB through Office of Research Management, to DMID and to the FMPOS, UMB and NIAID IRBs. No amendments will go into effect without written approval from HSRRB and these IRBs, except when the changes are necessary to eliminate immediate hazards to the participants.

III. IND AND STUDY SPONSORS' TERMINATION OF STUDY

The IND and study sponsors reserve the right to discontinue the clinical study at any time for medical or administrative reasons. When feasible, a 30-day written notification will be tendered.

IV. CASE REPORT FORM INSTRUCTIONS

Prior to screening the first potential participant, the investigator will provide a list showing the signature and hand-written initials of all individuals authorized to make or change entries on case report forms. If the authorized individuals should change during the study, the investigator is to inform the sponsor. Statistics Collaborative, Inc., will supply case report forms for recording all data. It is the responsibility of the investigator or co-investigators to ensure that case report forms are legible and completely filled in with a black ink fountain or ballpoint pen. Errors must be corrected by drawing a single line through the incorrect entry and writing in the new value/data positioned as close to the original as possible. The correction must then be initialed, dated and justified, where necessary, by the authorized individual making the change. The original entry must not be obliterated, overwritten or erased when a correction is made. Every effort will be made by the investigators or designated staff to complete the relevant sections of the case report form as soon as feasible following a visit. Similarly, when a subject completes the study, every effort will be made to complete the CRF as soon as the last data become available. As soon as the subject has completed/withdrawn from the study and the case report form is completed the

principal investigator or designated physician(s) under his/her supervision will sign the study conclusion pages of the case report form to confirm that they have reviewed the data and that the data are completed and accurate. An original (top copy) case report form or log sheets must be submitted for all subjects who have undergone protocol specific procedures, whether or not the subject completed the study. While completed case report forms will be reviewed by a professional monitor at the study site, errors detected by subsequent in-house case report form review may necessitate clarification or correction of errors and documentation and approval by the investigator. Whenever possible the investigator will assist in clarification or correction of errors detected after study finalization within 48 hours of them being brought to the attention of the investigator. Any questions or comments related to the case report form will be directed to the assigned Site Monitor.

V. MONITORING BY USAMMDA (i.e. THE IND SPONSORS)

Monitoring visits by a professional representative of the IND sponsors will be scheduled to take place before entry of the first subject, during the study at appropriate intervals and after the last subject has completed. It is anticipated that monitoring visits will occur at regular intervals. These visits are for the purpose of confirming that the studies are being conducted in compliance with the relevant Good Clinical Practice regulations/ guidelines, verifying adherence to the protocol and the completeness and exactness of data entered on the case report form and Vaccine Inventory Forms.

VI. ARCHIVING OF DATA

The investigator/ institution will maintain all study documentation until at least 2 years after the last approval of a marketing application in an ICH region and until there are no pending or contemplated marketing applications in an ICH region or at least 2 years have elapsed since the formal discontinuation of the clinical development of the investigational product. These documents will be retained for a longer period however if required by the applicable regulatory requirements or by an agreement with the study sponsor or IND sponsor. It is the responsibility of both sponsors to inform the investigator/institution as to when these documents no longer need to be retained. The investigator/ institution will take measures to prevent accidental or premature destruction of these documents. Similarly, the sponsor-specific study documentation will be retained until at least 2 years after the last approval of a marketing application in an ICH region and until there are no pending or contemplated marketing applications in an ICH region or at least 2 years have elapsed since the formal discontinuation of clinical development of the investigational product. These documents will be retained for a longer period however if required by the applicable regulatory requirements or if needed by the sponsors. The sponsors will inform the investigator/institution in writing of the need for record retention and will notify the investigator/institution in writing when the study-related records are no longer needed. Following the policy of the U.S. Army Medical Research and Materiel Command, data sheets including name, study number, address (when one is available) and dates will be completed on all volunteers participating in research for entry into the Command's Volunteer Registry Data Base. The intent of the data base is to readily answer questions concerning an individual's participation in research sponsored by USAMRMC and to ensure that the USAMRMC can exercise its obligation to ensure research volunteers are adequately warned (duty to warn) of risks and to provide new information as it becomes available. The information will be stored at USAMRMC for a minimum of 75 years.

VII. AUDITS

For the purpose of compliance with current Good Clinical Practice and Regulatory Agency Guidelines it may be necessary for a Drug Regulatory Agency to conduct a site audit. This may occur at any time from start to after conclusion of the study. When an investigator signs the protocol, he agrees to permit Drug Regulatory Agencies and the sponsor access to source data/ documents. Furthermore, if an investigator refuses an inspection, his data will not be accepted in support of a New Drug Registration and/or Application. The Inspector will be especially interested in the following items:
- Log of visits from the sponsor's representatives
- IRB approval
- Vaccine accountability
- Approved study protocol and amendments
- Informed screening and research consent of the subjects (written or witnessed oral consent)
- Medical records supportive of case report form data
- Reports to the IRB and the sponsors
- Record retention

16.5 USAMRMC Specific Administrative Procedures

I MONITORING/QUALITY ASSURANCE

Monitoring of this protocol will be performed also by representatives of the USAMMDA Office of Quality Assurance, and possibly WHO and GSK. Study monitors will conduct a study initiation visit (study day 0), mid-study visits at regular intervals, and a study close-out visit soon after the conclusion of the study (approximately month 16). The monitor will review case report forms and will compare them against source documents to verify accurate data collection, to evaluate adherence to Good Clinical Practices, and to ensure completeness, accuracy, and integrity of study data. Copies of all regulatory documents will be on file in the MMVDU central data storage facility in Bamako. Source documents, will be kept in Bandiagara BMP clinic during the conduct of the study and then archived for long-term storage in Bamako in dedicated storage facility of MMVDU within MRTC. In addition, documentation of test article storage, inventory, and accountability will be maintained at the clinical site. The Principal Investigator will coordinate the responsibilities and duties of all the Associate Investigators, as well as other collaborating personnel through periodic meetings.

II EVALUATIONS DURING AND FOLLOWING THE PROJECT

The medical evaluations of participants will be recorded by one of the physician investigators on standard forms. Blood samples for antibody tests will be obtained by appropriately trained individuals. Consent forms along with a copy of the final approved protocol will be retained as described above in VII ARCHIVING DATA.

III WITHDRAWAL FROM PROTOCOL FOR INDIVIDUAL SUBJECTS

Participants will be allowed to withdraw from the study at any time.

IV AMENDMENT OF PROTOCOL

If amendments in the protocol are required, they will be submitted in writing to the

Scientific Review Committees and IRBs of the participating facilities and the Office of Regulatory Compliance and Quality, Office of the Surgeon General, US Army.

Protocol Amendments must be reviewed and approved by the FMPOS IRB, the WRAIR Scientific Review Committee, University of Maryland IRB, NIAID IRB and HSRRB prior to implementation.

If required, participants will be provided with a revised informed study consent document for their signature.

V DISPOSITION OF UNUSED MEDICATIONS

Unused investigational vaccine doses will be accounted for and will be returned to the manufacturer for safekeeping or disposed of according to the manufacturer's policy.

VI USE OF INFORMATION AND PUBLICATIONS ARISING FROM THIS STUDY

It is anticipated that the results of this study will be presented to the scientific community via oral presentations at meetings and written publications in scientific journals. The data to be presented and the authorship will be discussed between investigators and sponsors prior to any official communication.

The official final report will be submitted through appropriate channels and upon approval by the WRAIR, Dept. of Immunology to the Human Use Review and Regulatory Affairs Division at Ft. Detrick, MD. This report will contain detailed information about the participants, their tolerance of the vaccines, their side effects and laboratory abnormalities, as well as their overall immune responses to immunization.
